# Supplementary material for: Chiral methionine oxidation reagents reveal stereospecific proteome modifications
Source: bioRxiv. 2026 Mar 26:2026.03.24.713977. Preprint. [Version 1] doi: 10.64898/2026.03.24.713977 (PMC13042024; doi:10.64898/2026.03.24.713977)
Supplement: Supplement 1 [file media-1.pdf]

Supplementary Materials

Chiral methionine oxidation reagents reveal stereospecific proteome modifications

Authors: Angel Gonzalez-Valero,[1,6†] Annika C. S. Page,[1†] Jayde M. Bertoch,[1,6] Fadi Alsarhan,[1] Jaehee Kim,[6] Amr A. Alazali,[3] Ritwik R. Srinivas,[3] Xiao Xie,[1,6] Audrey G. Reeves,[1] Kacper Skakuj,[1] Theodore G. Coffey,[1] Scott C. Virgil,[4] Jordan Nafie,[7] Dan He,[1] Nam Dao,[1] Amanda L. Gunawan,[2] Rina Dukor,[7] Andreas Stahl,[2] F. Dean Toste,[1*] and Christopher J. Chang.[1,5,6*]

[1]Department of Chemistry, University of California, Berkeley, Berkeley, CA 94720, USA.

[2]Department of Nutritional Sciences and Toxicology, University of California, Berkeley, Berkeley, CA 94720, USA.

[3]Department of Electrical Engineering and Computer Sciences, University of California, Berkeley, Berkeley CA 94720, USA.

[4]Division of Chemistry and Chemical Engineering, California Institute of Technology, Pasadena, CA 91125, USA.

[5]Department of Molecular and Cell Biology, University of California, Berkeley, Berkeley, CA 94720, USA.

[6]Department of Chemistry, Princeton University, Princeton, NJ 08544, USA.

[7]BioTools Inc, West Palm Beach, FL 33407, USA

*Corresponding author. Email: chrischang@princeton.edu (C.J.C.); fdtoste@berkeley.edu (F.D.T.)

†These authors contributed equally to this work.

The file includes:

- Materials and Methods
- Tables S1-3
- NMR Spectra

Materials and Methods

General methods: Reactions using moisture- or air-sensitive reagents were carried out in flame-dried glassware under an inert atmosphere of N_2. Solvent was passed over activated alumina and stored under argon before use when dry solvent was required. All other commercially purchased chemicals were used as received. Compound **AT-3** was prepared according to Chen et al.[49] without modification. Merck 60 F254 silica gel pre-

coated sheets (0.25 mm thick) were used for analytical thin layer chromatography and visualized by fluorescence quenching under UV light or by staining with $KMnO_4$ or Ninhydrin. Flash chromatography was performed using a CombiFlash NextGen 100 or Biotage Selekt automated chromatography system with pre-packed silica gel columns. ^{1}H and ^{13}C NMR spectra were collected at 298 K in $CDCl_3$ or CD_3OD (Cambridge Isotope Laboratories, Cambridge, MA) using Bruker AVQ-400, AVB-400, AV-500, AV-600, NEO-500, or NEO-501 instruments at the Pines Magnetic Resonance Center's Core NMR Facility at the University of California, Berkeley. All chemical shifts are reported in the standard notation of δ parts per million relative to the residual solvent peak at 7.26 ($CDCl_3$) or 3.31 (CD_3OD) for ^{1}H and 77.16 ($CDCl_3$) or 49.00 (CD_3OD) for ^{13}C as an internal reference. Splitting patterns are indicated as follows: br, broad; s, singlet; d, doublet; t, triplet; m, multiplet; dd, doublet of doublets. Enantioenrichment was determined by chiral HPLC using a Daicel CHIRALPAK® ADH column (4.6 x 250 mm) located in the Toste group at the University of California, Berkeley or by chiral SFC using a Chiral Technologies AD-H or OJ-H column (4.6 x 250 mm) located at the California Institute of Technology. Absolute configuration of oxaziridine enantiomers and sulfilimine products was determined by VCD. Experimental spectra were collected on a Chiral*IR-2X™* Dual*PEM* FT-VCD spectrometer (BioTools Inc, Jupiter, FL). Theoretical VCD spectra were calculated using Gaussian09 and compared using the CompareVOA™ program (BioTools Inc). Low-resolution electrospray mass spectral analyses were carried out using LC-MS (Agilent Technology 6130, Quadrupole LC/MS and Advion Expression-L Compact Mass Spectrometer). High-resolution mass spectral analyses (ESI-MS) were carried out using an Agilent 6230 LC-TOF (ESI) located in the Toste group at the University of California, Berkeley. All aqueous solutions for biological experiments were prepared using Milli-Q water, and all *in vitro* experiments were carried out in PBS, pH 7.4, unless otherwise noted. All biological experiments were prepared using freshly prepared aliquots.

Chemistry Methods

Synthesis of ChURRO-1:

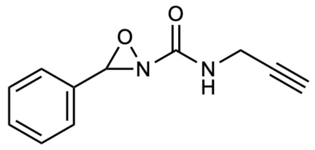

Compound **ChURRO-1** and its precursors were synthesized as a racemic mixture of enantiomers using previously reported procedures[28]. The NMR spectra of **ChURRO-1** were in accordance with previously published spectra.

Synthesis of ChURRO-2:

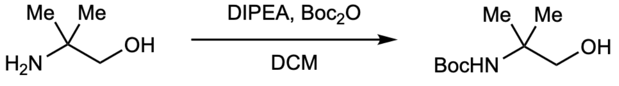

SI-1

***tert*-Butyl (1-hydroxy-2-methylpropan-2-yl)carbamate (SI-1)**

To a solution of 2-amino-2-methyl propanol (9.54 mL, 100 mmol, 1.11 equiv.) in DCM (20 mL) was added DIPEA (15.68 mL, 90 mmol, 1.00 equiv.) and Boc anhydride (19.64 g, 90 mmol, 1.00 equiv.) in DCM (23.75 mL). The reaction was allowed to stir at room temperature overnight. After 16 hours, the reaction was quenched by addition of 10% aqueous citric acid, diluted with DCM, and washed successively with water and brine. The organic layers were dried over $MgSO_4$, filtered, and concentrated under reduced pressure to give **SI-1** as a white powder (14.50 g, 76.6 mmol, 85% yield). ^{1}H NMR (600 MHz, $CDCl_3$) δ 4.65 (s, 1H), 4.00 (s, 1H), 3.65 – 3.50 (m, 2H), 1.43 (s, 9H), 1.25 (s, 6H). ^{13}C NMR (151 MHz, $CDCl_3$) δ 156.30, 79.94, 71.05, 54.48, 28.51, 24.88. Compound did not ionize on ESI.

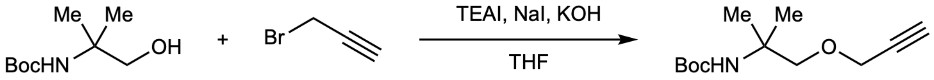

SI-2

***tert*-Butyl (2-methyl-1-(prop-2-yn-1-yloxy)propan-2-yl)carbamate (SI-2)**

A flame-dried round bottomed flask equipped with a magnetic stir bar was charged with THF (20 mL), alcohol **SI-1** (1.50 g, 7.93 mmol, 1.00 equiv.), propargyl bromide (80 wt% in toluene, 1.06 mL, 9.52 mmol, 1.20 equiv.), sodium iodide (0.204 g, 1.36 mmol, 0.17 equiv.), TEAI (0.204 g, 0.79 mmol, 0.10 equiv.), and KOH (0.89 g, 15.86 mmol, 2.00 equiv.). The resulting orange suspension was stirred vigorously overnight and quenched by addition of 20 mL H_2O and washed with EtOAc (3 x 50 mL). The combined organic layers were dried over Na_2SO_4, filtered, and concentrated under reduced pressure. The crude yellow oil was purified using flash column chromatography (15% EtOAc/Hexanes) to give **SI-2** as a yellow oil (1.06 g, 4.67 mmol, 59% yield). ^{1}H NMR (600 MHz, $CDCl_3$) δ 4.70 (s, 1H), 4.16 (d, *J* = 2.4 Hz, 2H), 3.47 (s, 2H), 2.42 (t, *J* = 2.4 Hz, 1H), 1.43 (s, 9H), 1.30 (s, 6H). ^{13}C NMR (151 MHz, $CDCl_3$) δ 154.95, 79.81, 79.01, 76.25, 74.61, 58.67, 52.66, 28.58, 24.38. Compound did not ionize on ESI.

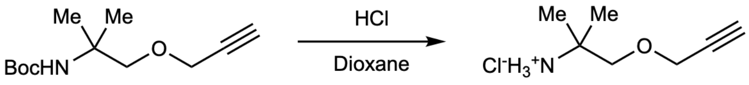

SI-3

2-Methyl-1-(prop-2-yn-1-yloxy)propan-2-aminium chloride (SI-3)

To a flame-dried round bottomed flask equipped with a magnetic stir bar was added a solution of carbamate **SI-2** (2.85 g, 12.52 mmol, 1.00 equiv.) and anhydrous HCl (27.54 mmol, 2.20 equiv., 4M in 1,4-dioxane) in anhydrous MeOH (25 mL). The reaction was allowed to stir overnight, and the resulting pink solution was concentrated under reduced pressure. The crude pink solid was washed with cold Et_2O to afford the HCl salt of the

amine as a white crystalline solid (1.50 g, 9.17 mmol, 73% yield) that was used immediately in the next step without further purification.

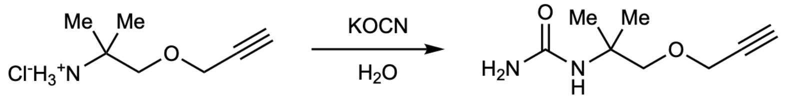

SI-4

1-(2-Methyl-1-(prop-2-yn-1-yloxy)propan-2-yl)urea (SI-4)

To a solution of amine **SI-3** (1.50 g, 9.17 mmol, 1.00 equiv.) in H_2O (9.17 mL) was added potassium cyanate (2.98 g, 36.68 mmol, 4.00 equiv.). The reaction was heated to 60 °C and allowed to stir overnight. After 16 hours, the reaction was cooled to room temperature and extracted with EtOAc (5 x 100 mL). The combined organic layers were dried over $MgSO_4$, filtered, and concentrated under reduced pressure to afford the urea **SI-4** as a white powder (1.05 g, 6.19 mmol, 68% yield). ^1H NMR (500 MHz, $CDCl_3$) δ 4.86 (s, 1H), 4.47 (s, 2H), 4.17 (d, J = 2.4 Hz, 2H), 3.51 (s, 2H), 2.44 (t, J = 2.4 Hz, 1H), 1.34 (s, 6H). ^{13}C NMR (126 MHz, $CDCl_3$) δ 158.41, 79.64, 76.96, 74.80, 58.69, 52.99, 24.73. HRMS (ESI) calculated for $[C_8H_{14}N_2O_2+H]^+$: required m/z 171.1128, found m/z 171.1124.

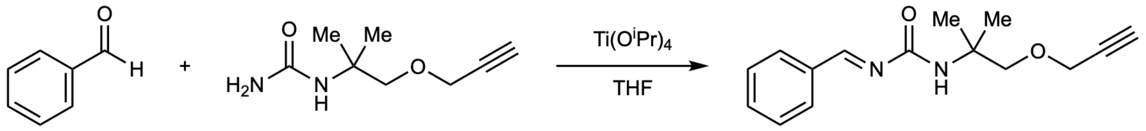

SI-5

N-Benzylidene-3-(2-methyl-1-(prop-2-yn-1-yloxy)propan-2-yl)urea (SI-5)

A flame-dried round bottomed flask equipped with a magnetic stir bar was charged with urea **SI-4** (1.05 g, 6.19 mmol, 1.00 equiv.) and evacuated and backfilled with N_2 twice. The urea was dissolved in THF (18.76 mL), to which benzaldehyde (0.76 mL, 7.43 mmol, 1.20 equiv.) and $Ti(O^iPr)_4$ (2.57 mL, 8.67 mmol, 1.40 equiv.) were added. The resulting mixture was stirred vigorously overnight. After 16 hours, the reaction was concentrated under reduced pressure and used immediately in the next step without further purification.

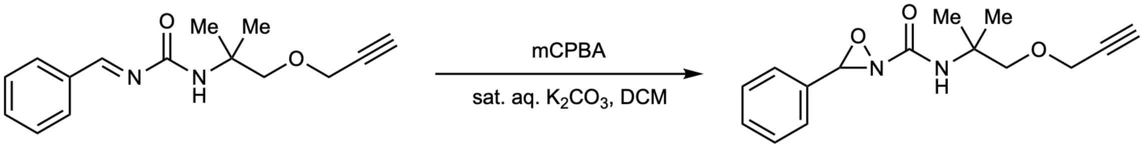

ChURRO_2

***N*-(2-Methyl-1-(prop-2-yn-1-yloxy)propan-2-yl)-3-phenyl-1,2-oxaziridine-2-carboxamide (ChURRO-2)**

To a solution of DCM (20 mL) and sat. aq. K_2CO_3 (20 mL) was added mCPBA (3.16 g, 18.57 mmol, 3.00 equiv.). The resulting slurry was stirred vigorously at room temperature for 10 minutes at which point a suspension of crude imine **SI-5** in DCM (15 mL) was added. The resulting yellow solution was allowed to stir at room temperature. After 8 hours, the reaction was stopped by addition of H_2O (100 mL) and extracted with DCM (3 x 50 mL). The combined organic layers were dried over $MgSO_4$, filtered, and concentrated under reduced pressure. The resulting oil was purified using flash column

chromatography (DCM/MeOH) to afford the racemic **ChURRO-2** as a clear oil. (0.226 g, 0.83 mmol, 13% yield over 2 steps). ^1H NMR (600 MHz, $CDCl_3$) δ 7.50 – 7.38 (m, 5H), 6.20 (s, 1H), 4.98 (s, 1H), 4.24 – 4.16 (m, 2H), 3.59 (d, *J* = 9.0 Hz, 1H), 3.50 (d, *J* = 9.0 Hz, 1H), 2.47 (t, *J* = 2.4 Hz, 1H), 1.41 (s, 3H), 1.39 (s, 3H). ^{13}C NMR (151 MHz, $CDCl_3$) δ 161.12, 132.81, 131.02, 128.73, 128.12, 79.56, 79.33, 75.43, 75.02, 58.68, 53.99, 23.98, 23.73. HRMS (ESI) calculated for $[C_{15}H_{18}N_2O_3+H]^+$: required *m/z* 275.1390, found *m/z* 275.1383.

Synthesis of ChURRO-3:

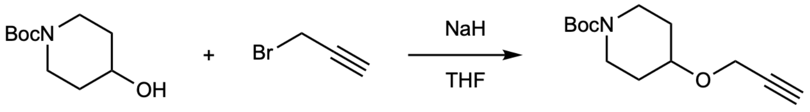

SI-6

***tert*-butyl 4-(prop-2-yn-1-yloxy)piperidine-1-carboxylate (SI-6)**

A flame-dried round bottomed equipped with a magnetic stir bar was charged with *tert*-butyl 4-hydroxypiperidine-1-carboxylate (5.00 g, 24.8 mmol, 1.00 equiv.) and evacuated and backfilled with N_2, after which anhydrous THF (80 mL) was added via syringe. NaH (60% suspension in mineral oil, 1.99 g, 49.70 mmol, 2.00 equiv.) was added in portions under an N_2 blanket over the course of a few minutes (bubbling observed). The cloudy grey reaction mixture was allowed to stir at ambient temperature for 45 minutes, after which propargyl bromide (80% wt in toluene, 1.39 mL, 67.10 mmol, 2.70 equiv.) was added dropwise via syringe. The brown reaction was stirred at ambient temperature for 3 hours and then cooled in an ice bath. Water (50 mL) was slowly added (vigorous bubbling) to quench the reaction and it was extracted with EtOAc (200 mL). The aqueous layer was washed with EtOAc (3 x 50 mL). The combined organic layers were dried over $MgSO_4$, filtered, and concentrated under reduced pressure. The resulting residue was purified via flash chromatography (0% to 20% EtOAc/Hexanes) to afford **SI-6** as a yellow oil that solidified upon storage at –20 °C (4.01 g, 16.62 mmol, 67% yield). ^1H NMR (600 MHz, $CDCl_3$) δ 4.14 (d, J = 2.4 Hz, 2H), 3.78 – 3.61 (m, 3H), 3.12 – 2.97 (m, 2H), 2.38 (t, J = 2.4 Hz, 1H), 1.84 – 1.73 (m, 2H), 1.57 – 1.44 (m, 2H), 1.40 (s, 9H). ^{13}C NMR (151 MHz, $CDCl_3$) δ 154.80, 80.10, 79.48, 74.16, 73.69, 55.16, 41.38, 40.91, 30.79, 28.45. HRMS (ESI) calculated for $[C_{13}H_{21}NO_3+Na]^+$: required *m/z* 262.14136, found *m/z* 262.14140.

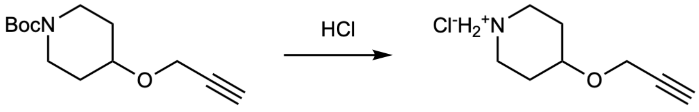

SI-7

4-(prop-2-yn-1-yloxy)piperidin-1-ium chloride (SI-7)

To a flame-dried round bottomed flask equipped with a magnetic stir bar was added a solution of carbamate **SI-6** (2.00 g, 8.36 mmol, 1.00 equiv.) and HCl (20.90 mL, 83.60 mmol, 10 equiv., 4M in 1,4-dioxane). The reaction mixture was allowed to stir for 2 hours and then concentrated under reduced pressure . The crude solid was washed with cold Et_2O to afford **SI-7** as an off-white solid (1.25 g, 7.12 mmol, 85% yield) that was used immediately in the next step without further purification.

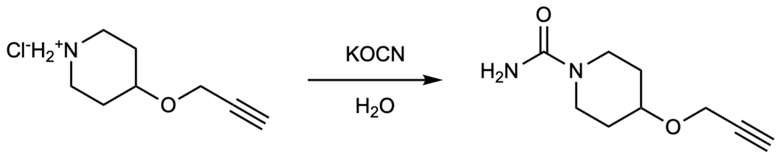

SI-8

4-(prop-2-yn-1-yloxy)piperidine-1-carboxamide (SI-8)

To a solution of amine **SI-7** (1.00 g, 5.70 mmol, 1.00 equiv.) in H_2O (5.70 mL) was added potassium cyanate (0.92 g, 11.40 mmol, 2.00 equiv.). The reaction was heated to 60 °C and allowed to stir for 6 hours, after which the solvent was removed under reduced pressure. The residue was washed with 50 mL of 85/15 $CHCl_3$/IPA, and the suspension was filtered, dried over Na_2SO_4, and concentrated under reduced pressure to afford **SI-8** as a white solid (0.88g, 4.85 mmol, 85% yield). ^{1}H NMR (600 MHz, CD_3OD) δ 4.22 (d, J = 2.4 Hz, 2H), 3.81 – 3.74 (m, 1H), 3.73 – 3.65 (m, 2H), 3.23 – 3.09 (m, 2H), 2.82 (t, J = 2.4 Hz, 1H), 1.98 – 1.74 (m, 2H), 1.68 – 1.38 (m, 2H). ^{13}C NMR (151 MHz, CD_3OD) δ 161.04, 81.09, 75.42, 74.67, 56.04, 42.41, 31.81. HRMS (ESI) calculated for $[C_9H_{14}N_2O_2+H]^+$: required m/z 183.11280, found m/z 183.11271.

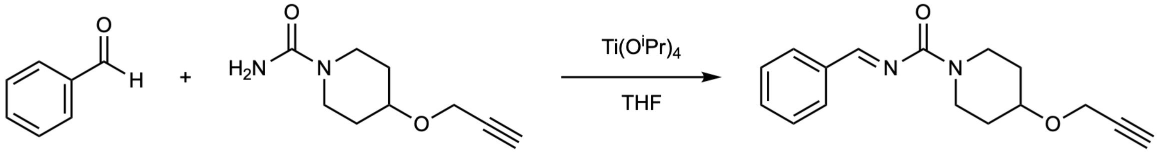

SI-9

N-benzylidene-4-(prop-2-yn-1-yloxy)piperidine-1-carboxamide (SI-9)

A flame-dried round bottomed flask equipped with a magnetic stir bar was charged with urea **SI-8** (0.324 g, 1.78 mmol, 1.00 equiv.) and evacuated and backfilled with N_2 twice. The urea was dissolved in THF (1 mL), to which benzaldehyde (0.22 mL, 2.14 mmol, 1.20 equiv.) and $Ti(O^iPr)_4$ (0.76 mL, 2.49 mmol, 1.40 equiv.) were added. The resulting mixture was stirred vigorously overnight. After 16 hours, the reaction was concentrated under reduced pressure and used immediately in the next step without further purification.

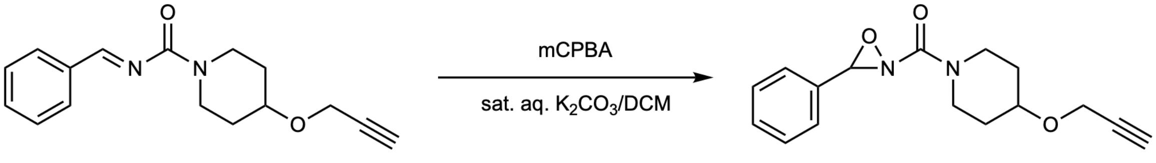

ChURRO_3

(3-phenyl-1,2-oxaziridin-2-yl)(4-(prop-2-yn-1-yloxy)piperidin-1-yl)methanone (ChURRO-3)

To a solution of DCM (10 mL) and sat. aq. K_2CO_3 (7.5 mL) was added mCPBA (0.92 g, 5.34 mmol, 3.00 equiv.). The resulting slurry was stirred vigorously at room temperature for 10 minutes at which point a suspension of crude imine **SI-9** in DCM (5 mL) was added. The resulting yellow solution was allowed to stir at room temperature. After 2 hours, the reaction was stopped by addition of H_2O (25 mL) and extracted with DCM (3 x 50 mL). The combined organic layers were dried over Na_2SO_4, filtered, and concentrated under

reduced pressure. The resulting oil was purified via flash chromatography (50:50:0 to 45:45:10 DCM/Hexanes/Et_2O) to afford racemic **ChURRO-3** as a clear oil. (0.217 g, 0.80 mmol, 45% yield over 2 steps). ^1H NMR (600 MHz, $CDCl_3$) δ 7.57 – 7.36 (m, 5H), 5.22 (s, 0.5H), 5.21 (s, 0.5H), 4.20 (d, J = 2.4 Hz, 1H), 4.19 (d, J = 2.4 Hz, 1H), 4.12 – 4.00 (m, 0.5H), 3.96 – 3.77 (m, 2H), 3.76 – 3.64 (m, 1H), 3.56 – 3.48 (m, 1H), 3.37 (m, 0.5H), 2.42 (t, J = 2.4 Hz, 1H), 2.04 – 1.83 (m, 2H), 1.79 – 1.61 (m, 2H). ^{13}C NMR (151 MHz, $CDCl_3$) δ 160.74, 160.73, 133.06, 133.02, 130.85, 128.77, 128.02, 79.97, 78.08, 78.03, 74.45, 72.82, 72.47, 55.57, 55.45, 42.20, 41.86, 41.24, 40.87, 31.11, 30.98, 30.43, 30.22. HRMS (ESI) calculated for $[C_{16}H_{18}N_2O_3+H]^+$: required m/z 287.13902, found m/z 287.13911.

Synthesis of ChURRO-4:

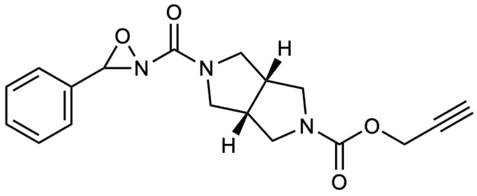

Compound **ChURRO-4** and its precursors were synthesized as a racemic mixture of enantiomers using previously reported procedures[34]. The NMR spectra of **ChURRO-4** were in accordance with previously published spectra.

Synthesis of ChURRO-5:

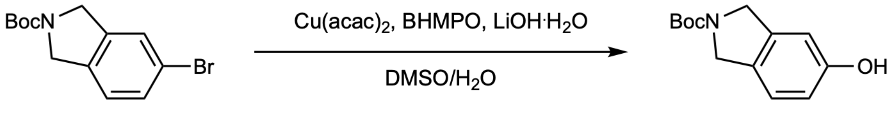

SI-10

***tert*-butyl 5-hydroxyisoindoline-2-carboxylate (SI-10)**

A flame-dried round bottomed flask equipped with a magnetic stir bar was charged with *tert*-butyl 5-bromoisoindoline-2-carboxylate (5.00 g, 16.80 mmol, 1.00 equiv.), copper(II) acetylacetonate (0.088 g, 0.34 mmol, 0.02 equiv.), bis(4-hydroxy-2,6-dimethylphenyl)oxalamide (0.11 g, 0.34 mmol, 0.02 equiv.), and lithium hydroxide monohydrate (1.55 g, 36.90 mmol, 2.20 equiv.). The contents of the flask were evacuated and backfilled with N_2 three times, after which degassed DMSO (13 mL) and degassed H_2O (3.25 mL) were added via syringe. The reaction was heated to 80 ℃ and allowed to stir for 24 hours. The reaction was then allowed to cool down to ambient temperature and acidified with 2M HCl (pH <5) and extracted with EtOAc (3 x 50 mL). The combined organic layers were washed with brine, dried over Na_2SO_4, filtered, and concentrated under reduced pressure. The resulting residue was purified using flash chromatography (10% to 60% EtOAc/Hexanes) to afford **SI-10** as an off-white solid (2.40 g, 10.25 mmol, 61% yield). Note: compound exists as a 1:1 mixture of rotamers in $CDCl_3$ at 298 K. ^1H NMR (600 MHz, $CDCl_3$) δ 7.08 (d, J = 8.2 Hz, 0.5H), 7.05 (d, J = 8.2 Hz, 0.5H), 6.84 – 6.71 (m, 2H), 6.41 (s, 0.5H), 6.36 (s, 0.5H), 4.62 (d, J = 14.6 Hz, 2H), 4.58 (d, J = 14.6 Hz, 2H), 1.52 (s, 9H). ^{13}C NMR (151 MHz, $CDCl_3$) δ 156.05, 155.99, 155.03, 154.98,

138.82, 138.41, 128.81, 128.52, 123.75, 123.50, 115.06, 115.04, 109.79, 109.53, 80.22, 52.54, 52.20, 51.98, 51.62, 28.71. HRMS (ESI) calculated for $[C_{13}H_{17}NO_3+Na]^+$: required m/z 258.11006, found m/z 258.10990.

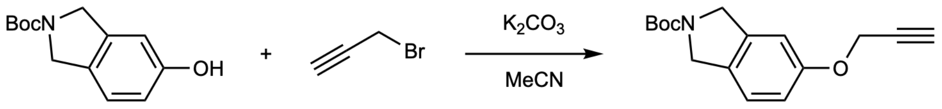

SI-11

tert-butyl 5-(prop-2-yn-1-yloxy)isoindoline-2-carboxylate (SI-11)

A flame-dried round bottomed flask equipped with a magnetic stir bar was charged with **SI-10** (1.50 g, 6.40 mmol, 1.00 equiv.) and potassium carbonate (1.80 g, 13.00 mmol, 2.00 equiv.). The contents of the flask were evacuated and backfilled with N_2 three times, after which anhydrous MeCN (32 mL) and propargyl bromide (80% wt in toluene, 0.76 mL, 7.00 mmol, 1.10 equiv.) were added via syringe. The reaction was heated to 40 °C and allowed to stir overnight. The reaction was then allowed to cool down to ambient temperature and the solids were filtered, washing with EtOAc (2 x 10 mL). The solvent was removed under reduced pressure and the resulting residue was purified via flash chromatography (0% to 30% EtOAc/Hexanes) to afford **SI-11** as an off-white solid (1.34 g, 4.93 mmol, 77% yield). Note: compound exists as a 1:1 mixture of rotamers in $CDCl_3$ at 298 K. ^1H NMR (600 MHz, $CDCl_3$) δ 7.18 (d, J = 8.2 Hz, 0.5H), 7.13 (d, J = 8.2 Hz, 0.5H), 6.93 – 6.77 (m, 2H), 4.68 (apparent t, J = 2.2 Hz, 2H), 4.66 – 4.57 (m, 4H), 2.51 (t, J = 2.5 Hz, 1H), 1.51 (s, 9H). ^{13}C NMR (151 MHz, $CDCl_3$) δ 157.43, 157.39, 154.63, 154.60, 138.94, 138.56, 130.46, 130.15, 123.68, 123.45, 114.92, 114.67, 109.18, 79.77, 78.59, 78.57, 75.74, 56.20, 52.55, 52.26, 51.89, 51.57, 28.67. HRMS (ESI) calculated for $[C_{16}H_{19}NO_3+Na]^+$: required m/z 296.12571, found m/z 296.12568.

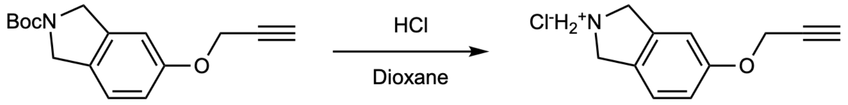

SI-12

5-(prop-2-yn-1-yloxy)isoindolin-2-ium chloride (SI-12)

To a flame-dried vial equipped with a magnetic stir bar was added a solution of carbamate **SI-11** (0.95 g, 3.50 mmol, 1.00 equiv.) and HCl (8.70 mL, 35.00 mmol, 10.00 equiv., 4M in 1,4-dioxane). The orange reaction mixture was allowed to stir for 2 hours, after which a pinkish-white precipitate crashed out. The crude solid was washed with Et_2O to afford **SI-12** as a white solid (0.73 g, 3.50 mmol, quantitative yield) that was used immediately in the next step without further purification.

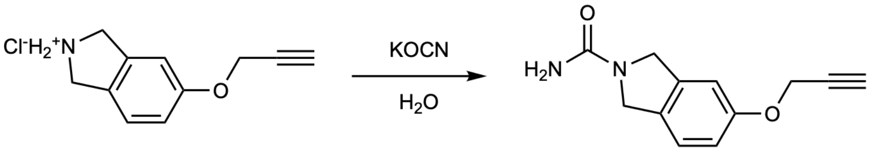

SI-13

5-(prop-2-yn-1-yloxy)isoindoline-2-carboxamide (SI-13)

To a solution of amine **SI-12** (0.73 g, 3.50 mmol, 1.00 equiv.) in H_2O (3.5 mL) was added potassium cyanate (0.85 g, 10.00 mmol, 3.00 equiv.). The reaction was heated to 60 °C and allowed to stir overnight. After 16 hours, the reaction was cooled to room temperature and extracted with EtOAc (5 x 50 mL). The combined organic layers were dried over MgSO4, filtered, and concentrated under reduced pressure to afford the urea **SI-13** as an off-white powder (0.545 g, 2.52 mmol, 72% yield). ^1H NMR (600 MHz, CD_3OD) δ 7.20 (d, J = 8.2 Hz, 1H), 6.97 – 6.88 (m, 2H), 4.71 (d, J = 2.4 Hz, 2H), 4.63 (s, 2H), 4.59 (s, 2H), 2.93 (t, J = 2.4 Hz, 1H). ^{13}C NMR (151 MHz, CD_3OD) δ 160.51, 159.12, 139.36, 130.69, 124.52, 116.15, 110.00, 79.74, 76.79, 56.89, 53.22, 52.56. HRMS (ESI) calculated for $[C_{12}H_{12}N_2O_2+H]^+$: required m/z 217.09715, found m/z 217.09785.

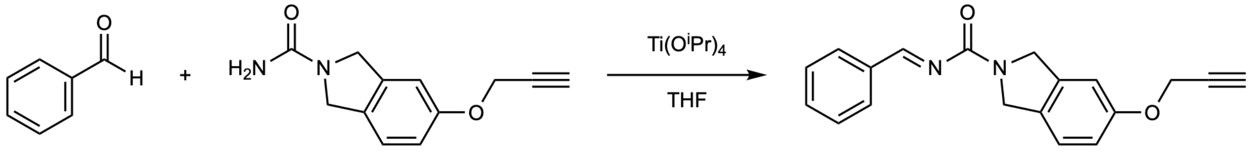

SI-14

N-benzylidene-5-(prop-2-yn-1-yloxy)isoindoline-2-carboxamide (SI-14)

A flame-dried round bottomed flask equipped with a magnetic stir bar was charged with urea **SI-13** (0.231 g, 1.07 mmol, 1.00 equiv.) and evacuated and backfilled with N_2 twice. The urea was dissolved in THF (11 mL), to which benzaldehyde (0.13 mL, 1.28 mmol, 1.20 equiv.) and $Ti(O^iPr)_4$ (0.45 mL, 1.50 mmol, 1.40 equiv.) were added. The resulting mixture was stirred vigorously overnight. After 16 hours, the reaction was concentrated under reduced pressure and used immediately in the next step without further purification.

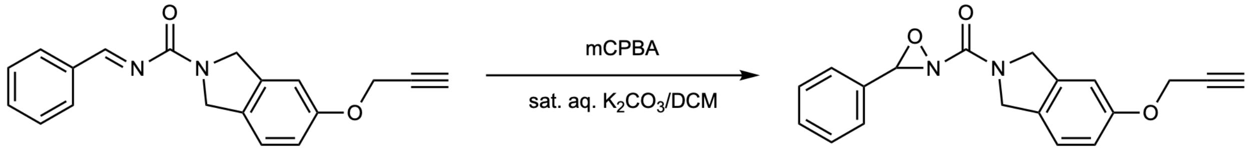

ChURRO_5

(3-phenyl-1,2-oxaziridin-2-yl)(5-(prop-2-yn-1-yloxy)isoindolin-2-yl)methanone (ChURRO-5)

To a solution of DCM (7.5 mL) and sat. aq. K_2CO_3 (5 mL) was added mCPBA (0.553 g, 3.20 mmol, 3.00 equiv.). The resulting slurry was stirred vigorously at room temperature for 10 minutes at which point a suspension of crude imine **SI-14** in DCM (2.5 mL) was added. The resulting yellow solution was allowed to stir at room temperature. After 2 hours, the reaction was stopped by addition of H_2O (50 mL) and extracted with DCM (3 x 50 mL). The combined organic layers were dried over Na_2SO4, filtered, and concentrated under reduced pressure. The resulting residue was purified using flash column chromatography (50:50:0 to 45:45:10 DCM/Hexanes/Et_2O) to afford the racemic **ChURRO-5** as an off-white solid. (0.133 g, 0.42 mmol, 39% yield over 2 steps). Note: compound exists as a 1:1 mixture of rotamers in $CDCl_3$ at 298 K. ^1H NMR (600 MHz, $CDCl_3$) δ 7.57 – 7.52 (m, 2H), 7.50 – 7.39 (m, 3H), 7.22 (d, J = 8.4 Hz, 0.5H), 7.16 (d, J = 8.4 Hz, 0.5H), 7.00 – 6.84 (m, 2H), 5.30 (s, 0.5H), 5.29 (s, 0.5H), 5.13 (d, J = 14.5 Hz, 0.5H), 5.08 (d, J = 14.5 Hz, 0.5H), 4.90 – 4.77 (m, 3H), 4.70 (d, J = 2.4 Hz, 1H), 4.69 (d,

J = 2.4 Hz, 1H), 2.53 (t, J = 2.4 Hz, 0.5H), 2.52 (t, J = 2.4 Hz, 0.5H). ^{13}C NMR (151 MHz, $CDCl_3$) δ 160.57, 160.52, 157.80, 157.75, 137.80, 137.05, 133.07, 130.97, 129.24, 128.83, 128.53, 128.10, 123.83, 123.68, 115.41, 115.37, 109.28, 109.20, 78.42, 78.04, 78.03, 75.91, 56.26, 56.23, 53.41, 52.80, 52.72, 52.12. HRMS (ESI) calculated for $[C_{19}H_{16}N_2O_3+H]^+$: required m/z 321.12337, found m/z 321.12337.

Preparative SFC separation: Preparative oxaziridine separation was conducted at the California Institute of Technology by Dr. Scott Virgil via chiral supercritical fluid chromatography (SFC). The enantiomers were purified on a JASCO System 2000 preparative SFC system with a Chiral Technologies 21.2 x 250 mm AD-H column, a Chiral Technologies 20 x 250 mm OZ-H column, or a Chiral Technologies 10 x 250 mm OJ-H column. The fractions were analyzed on a Thar SFC system with a 4.6 x 250 mm Chiral Technologies AD-3, AD-H or OJ-H column.

ChURRO-1: Enantiomers were purified via preparatory SFC (OJ-H column, 30% IPA/CO_2, 18 mL/min, 210 nm).
(*R*)-ChURRO-1 is the first eluting enantiomer on OJ-H column. Enantiopurity was determined by analytical chiral SFC (OJ-H column, 30% IPA/CO_2, 2.5 mL/min, 210 nm) retention time: 2.2 min (99%)
(*S*)-ChURRO-1 is the second eluting enantiomer on OJ-H column. Enantiopurity was determined by analytical chiral SFC (OJ-H column, 30% IPA/CO_2, 2.5 mL/min, 210 nm) retention time: 2.8 min (99%)

ChURRO-2: Enantiomers were purified via preparatory SFC (OZ-H column, 25% IPA/CO_2, 25 mL/min, 210 nm).
(*R*)-ChURRO-2 is the first eluting enantiomer on AD-H column and the second eluting enantiomer on OZ-H column. Enantiopurity was determined by analytical chiral SFC (AD-H column, 10% IPA/CO_2, 2.5 mL/min, 210 nm) retention time: 4.4 min (99%). ^{1}H NMR (500 MHz, $CDCl_3$) δ 7.50 – 7.37 (m, 5H), 6.20 (s, 1H), 4.98 (s, 1H), 4.20 (t, *J* = 2.3 Hz, 2H), 3.59 (d, *J* = 9.0 Hz, 1H), 3.49 (d, *J* = 9.0 Hz, 1H), 2.47 (t, *J* = 2.4 Hz, 1H), 1.40 (d, *J* = 5.8 Hz, 6H). ^{13}C NMR (126 MHz, $CDCl_3$) δ 161.12, 132.78, 131.03, 128.73, 128.12, 79.55, 79.32, 75.41, 75.03, 58.67, 53.98, 23.96, 23.72. HRMS (ESI) calculated for $[C_{15}H_{18}N_2O_3+Na]^+$: required *m/z* 297.1210, found *m/z* 297.1211.
(*S*)-ChURRO-2 is the second eluting enantiomer on AD-H column and the first eluting enantiomer on OZ-H column. Enantiopurity was determined by analytical chiral SFC (AD-H column, 10% IPA/CO_2, 2.5 mL/min, 210 nm) retention time: 4.8 min (99%). ^{1}H NMR (500 MHz, $CDCl_3$) δ 7.50 – 7.37 (m, 5H), 6.20 (s, 1H), 4.98 (s, 1H), 4.20 (t, *J* = 2.3 Hz, 2H), 3.59 (d, *J* = 9.1 Hz, 1H), 3.49 (d, *J* = 9.1 Hz, 1H), 2.47 (t, *J* = 2.4 Hz, 1H), 1.40 (d, *J* = 5.8 Hz, 6H). ^{13}C NMR (126 MHz, $CDCl_3$) δ 161.11, 132.78, 131.02, 128.73, 128.11, 79.55, 79.32, 75.40, 75.03, 58.66, 53.98, 23.96, 23.71. HRMS (ESI) calculated for $[C_{15}H_{18}N_2O_3+Na]^+$: required *m/z* 297.1210, found *m/z* 297.1210.

ChURRO-3: Enantiomers were purified via preparatory SFC (AD-H column, 35% EtOH/CO_2, 35 mL/min, 210 nm).

(*R*)-ChURRO-3 is the first eluting enantiomer on AD-H column. Enantiopurity was determined by analytical chiral SFC (AD-H column, 35% EtOH/CO_2, 2.5 mL/min, 210 nm) retention time: 3.2 min (99%).
(*S*)-ChURRO-3 is the second eluting enantiomer on AD-H column. Enantiopurity was determined by analytical chiral SFC (AD-H column, 35% EtOH/CO_2, 2.5 mL/min, 210 nm) retention time: 4.3 min (99%).

ChURRO-4: Enantiomers were purified via preparatory SFC (OJ-H column, 35% EtOH/CO_2, 18 mL/min, 210 nm).
(*R*)-ChURRO-4 is the first eluting enantiomer on OJ-H column. Enantiopurity was determined by analytical chiral SFC (AD-H column, 35% EtOH/CO_2, 2.5 mL/min, 210 nm) retention time: 3.3 min (99%).
(*S*)-ChURRO-4 is the second eluting enantiomer on OJ-H column. Enantiopurity was determined by analytical chiral SFC (AD-H column, 35% EtOH/CO_2, 2.5 mL/min, 210 nm) retention time: 4.0 min (99%).

ChURRO-5: Enantiomers were purified via preparatory SFC (AD-H column, 40% IPA/CO_2, 35 mL/min, 210 nm).
(*R*)-ChURRO-5 is the first eluting enantiomer on AD-H column. Enantiopurity was determined by analytical chiral SFC (AD-3 column, 40% IPA/CO_2, 2.5 mL/min, 210 nm) retention time: 3.8 min (99%).
(*S*)-ChURRO-5 is the second eluting enantiomer on AD-H column. Enantiopurity was determined by analytical Chiral SFC (AD-3 column, 40% IPA/CO_2, 2.5 mL/min, 210 nm) retention time: 5.0 min (99%).

VCD analysis: Vibrational circular dichroism (VCD) measurements were conducted at BioTools (Jupiter, FL) by Jordan Nafie as follows:
Probe measurements: Dissolved E1 (first eluting enantiomer of ChURRO-probe) or E2 (second eluting enantiomer of ChURRO-probe) (7-10 mg) in 120-650 μL $CDCl_3$ (previously run through a plug of activated basic alumina) and transferred to a 100 μm BaF_2 IR cell. Simultaneous measurement of IR and VCD were taken for ~18 hours in one-hour blocks on a BioTools (Jupiter, FL) DualPEM FT-VCD spectrometer at 4 cm^{-1} resolution and PEM maximum frequencies both set to 1400 cm^{-1}. The first block of IR was solvent and water vapor corrected, and offset to zero at 2000 cm^{-1}. VCD was block averaged and corrected by half difference ((E1 – E2) / 2). Noise spectra were block averaged and used as is.

To analyze the stability of the enantiomeric pairs of ChURRO-2, the IR and VCD were measured simultaneously of each enantiomer in CD_3OD for 24 hours in 30 min blocks as described above. Various numbers of blocks were averaged to determine the optimal number of blocks required for reasonably good signal to noise ratio VCD data, arriving at 8 hours (16 blocks), resulting in 33 VCD spectra of 8 hour avg each. The raw VCD data was then baseline corrected using the half difference method as described above.

Calculations: The (*R*) enantiomer of each ChURRO-probe was constructed using BioTools (Jupiter, FL) ComputeVOA software. A thorough conformational search was performed at the molecular mechanics level (GMMX) using the MMF94 force field in a 7 kcal / mol energy window. The resulting 121 conformations were optimized at the DFT (Gaussian 09, Wallingford, CT) level (B3LYP / 6-31G(d) with CPCM - Chloroform) and the IR and VCD frequencies were also calculated at the same level. The conformations were separated into cis and trans oxaziridine isomers, and the 20 lowest energy conformers from each group (cis and trans) were re-optimized and frequencies calculated at the B3PW91 / cc-pVTZ level. The spectra from the resulting conformers were Boltzmann averaged using the electronic energy values and plotted using a line width of 5 cm^{-1} HWHH, and an x-axis scaling factor of 0.976 for comparison with the experimental IR and VCD data. Of the geometric isomers, it was determined that the trans was a good match with the data and the cis a poor match.

Methionine adduct:
Measurements: Two samples were received which were derived from (*R*)- and (*S*)-ChURRO-2 starting material. Each sample (~10 mg) was separately dissolved in 125 – 150 μL $CDCl_3$ (previously run through a plug of activated basic alumina) and transferred to a 100 μm BaF_2 IR cell. Simultaneous measurement of IR and VCD were taken for ~18 hours in one-hour blocks on a BioTools (Jupiter, FL) DualPEM FT-VCD spectrometer at 4 cm^{-1} resolution and PEM maximum frequencies both set to 1400 cm^{-1}. The first block of IR was solvent and water vapor corrected, and offset to zero at 2000 cm^{-1}. VCD was block averaged and corrected by subtraction of a solvent baseline which was previously acquired (18 hours averaged). Noise spectra were block averaged and used as is. The two samples had identical IR and nearly identical VCD – based on this, and the fact that the calculated (s*R*) and (s*S*) diastereomers had very different VCD spectra, the samples were determined to be identical.

Calculations: The (s*R*, *S*) and (s*S*, *S*) diastereomers were separately constructed using BioTools (Jupiter, FL) ComputeVOA software. A thorough conformational search was performed on each at the molecular mechanics level (GMMX) using the MMF94 force field in a 7 kcal / mol energy window. The resulting ~150 conformations for each diastereomer were optimized at the DFT (Gaussian 09, Wallingford, CT) level (B3LYP / 6-31G(d) with CPCM - Chloroform) and the IR and VCD frequencies were also calculated at the same level. The 20 lowest energy conformers from each group (s*R* or s*S*) were re-optimized and frequencies calculated at the B3LYP / 6-311G(3df,2pd) level. The spectra from the resulting conformers were Boltzmann averaged using the electronic energy values and plotted using a line width of 5 cm^{-1} HWHH, and an x-axis scaling factor of 0.982 for comparison with the experimental IR and VCD data. The (s*S*, *S*) DFT spectra were a much better match to the experimental than the (s*R*, *S*).

Diastereoselectivity studies:

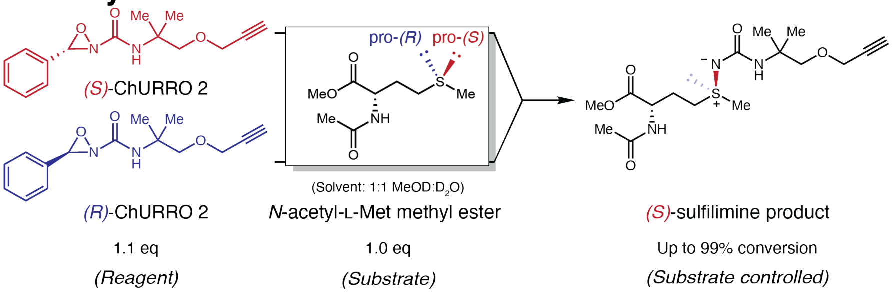

To an NMR tube was added (*R*)- or (*S*)-ChURRO-2 (9.1 mg, 0.033 mmol, 1.1 equiv.) and *N*-acetyl-L-methionine methyl ester (6.2 mg, 0.03 mmol, 1.0 equiv.) in 0.6 mL 1:1 CD_3OD:D_2O. The reaction was incubated and monitored by ^1H NMR. After 30 minutes the reaction was concentrated and purified using flash column chromatography (10% MeOH/DCM eluent) to give sulfilimine product which was analyzed by ^1H NMR, HRMS, VCD, and IR to confirm absolute configuration at sulfur.

(*R*)-ChURRO-2 product: 11.1 mg isolated. ^1H NMR (600 MHz, CD_3OD) δ 4.58 – 4.52 (m, 1H), 4.16 (d, *J* = 2.3 Hz, 2H), 3.74 (d, *J* = 1.4 Hz, 3H), 3.56 – 3.47 (m, 2H), 3.06 – 2.87 (m, 2H), 2.82 (t, *J* = 2.4 Hz, 1H), 2.63 (s, 3H), 2.30 – 2.23 (m, 1H), 2.10 – 2.02 (m, 1H), 2.01 (d, *J* = 3.0 Hz, 3H), 1.29 (d, *J* = 2.0 Hz, 6H, *overlap with grease*). HRMS (ESI): calculated for $[C_{16}H_{27}N_3O_5S+H]^+$: required *m/z* 374.1744, found *m/z* 374.1754.

(*S*)-ChURRO-2 product: 8.7 mg isolated. ^1H NMR (600 MHz, CD_3OD) δ 4.60 – 4.51 (m, 1H *overlap with impurity*), 4.16 (d, *J* = 2.4 Hz, 2H), 3.74 (d, *J* = 1.4 Hz, 3H), 3.56 – 3.47 (m, 2H), 3.07 – 2.87 (m, 2H), 2.82 (t, *J* = 2.4 Hz, 1H), 2.63 (s, 3H), 2.30 – 2.23 (m, 1H), 2.10 – 2.02 (m, 1H), 2.01 (d, *J* = 2.9 Hz, 3H), 1.29 (d, *J* = 2.0 Hz, 6H, *overlap with grease*). HRMS (ESI): calculated for $[C_{16}H_{27}N_3O_5S+H]^+$: required *m/z* 374.1744, found *m/z* 374.1754.

Energies of Optimized Geometries

structure	energy in kcal	Delta G (R)	Delta G (S)
methionine	-622532.5111		
(*R*)-ChURRO-2	-575332.7136		
(*S*)-ChURRO-2	-575332.711		
R SMs	-1197865.225	0.00	
S SMs	-1197865.222		0.00
*R*_s*S* TS1	-1197841.608	23.62	
*R*_s*R* TS1	-1197839.958	25.27	
*S*_s*R* TS1	-1197836.37		28.85
*S*_s*S* TS1	-1197841.263		23.96

*R*_s*R* int1	-1197870.053	-4.83	
*S*_s*R* int1	-1197876.253		-11.03
*R*_s*S* int1	-1197871.261	-6.04	
*S*_s*S*_int1	-1197875.102		-9.88
benzaldehyde	-216710.2069		
s*R* sulfilimine	-981174.3494		
s*S* sulfilimine	-981178.2042		
s*R* pdts	-1197884.556	-19.33	
s*S* pdts	-1197888.411		-23.19

XYZ Coordinates of Optimized Geometries

N-acetyl-L-methionine methyl ester

N	0.65411900	1.28373700	-0.63063100
H	0.67394800	1.50072700	-1.62215700
C	0.20752600	-0.03821200	-0.25997900
H	-0.22647500	-0.01087900	0.74624800
C	-0.86693200	-0.52788700	-1.23471700
C	1.37210700	-1.02085700	-0.20030600
H	-0.44932300	-0.54164600	-2.24861000
H	-1.12348200	-1.55819400	-0.97942900
C	-2.12242000	0.33996300	-1.23939900
O	2.52750200	-0.74691000	-0.44520300
H	-1.90358500	1.34430500	-1.61079500
H	-2.86359700	-0.10785400	-1.90675500
S	-2.88192900	0.58957700	0.39883800
C	-3.08367700	-1.13970500	0.91043700
H	-2.11972500	-1.61318600	1.11260800
H	-3.67292400	-1.13710400	1.82885200
H	-3.62096700	-1.70526300	0.14567900
C	1.39008400	2.11387000	0.15180500
O	1.90607900	3.12942500	-0.32064700
C	1.50547500	1.76242900	1.61243800
H	0.51396600	1.67082700	2.06495300
H	2.02875800	0.81108300	1.74297300
H	2.06385000	2.55160300	2.11320800
O	0.96357100	-2.22908100	0.18730600
C	1.99157200	-3.22648400	0.30399000
H	1.48659400	-4.13253000	0.63139400
H	2.47201100	-3.38397500	-0.66284300
H	2.73335600	-2.91347100	1.04043000

(*R*)-ChURRO-2

O	-0.75286600	-2.01503000	-1.28781200
C	-0.73823000	-1.62652100	-0.12372400
N	-1.49884200	-0.67429500	0.41967100
C	-2.54546200	0.07552400	-0.30826300
C	-3.47507900	-0.88285900	-1.05975100
H	-4.35977100	-0.33097900	-1.38772200
H	-2.99422800	-1.32019100	-1.93542800
H	-3.79658200	-1.68899300	-0.39502900
C	-3.34987100	0.84248800	0.73808100
H	-3.87264800	0.14228700	1.39580600
H	-4.09182300	1.46895400	0.23660600
H	-2.70751300	1.48497300	1.34422400
C	-1.91757400	1.03797400	-1.32715400
O	-1.33396900	2.20605600	-0.77154300
C	-0.04264300	2.04045400	-0.20766500
C	-0.08010900	1.93413100	1.26165500
C	-0.12016800	1.85755000	2.46610100
H	-0.15078100	1.78926800	3.53395200
H	0.54989300	2.92037600	-0.47967100
H	0.45425600	1.15664300	-0.62800000
H	-1.18036600	0.49685600	-1.93604000
H	-2.71414200	1.39861400	-1.98479100
H	-1.28815600	-0.39588600	1.37193500
N	0.07591300	-2.38132600	0.80051000
O	0.50089800	-1.59383500	1.95780400
C	1.43868600	-1.96146700	0.97130200
C	2.06291400	-0.85994800	0.18326000
C	2.59131100	0.23478800	0.86888600
C	3.21671400	1.25913800	0.16276500
C	3.31959000	1.18632000	-1.22581900
C	2.80694800	0.08378700	-1.90794000
C	2.18390000	-0.94560900	-1.20558300
H	1.78240400	-1.80612900	-1.73050600
H	2.89178300	0.02362100	-2.98787700
H	3.80212100	1.98756600	-1.77612200
H	3.61819100	2.11493300	0.69569800
H	2.49824600	0.28743900	1.94934100
H	2.10155500	-2.77202700	1.26786000

(*S*)-ChURRO-2

O	0.75286000	-2.01424800	-1.28854500
C	0.73805400	-1.62625400	-0.12427900

N	1.49877100	-0.67441400	0.41970000
C	2.54560300	0.07554400	-0.30781400
C	3.34968600	0.84243500	0.73880000
H	4.09191500	1.46876500	0.23757200
H	2.70722300	1.48502000	1.34471400
H	3.87211900	0.14215800	1.39670900
C	3.47552200	-0.88278600	-1.05903200
H	2.99499600	-1.32011700	-1.93488500
H	3.79681600	-1.68892600	-0.39422100
H	4.36029700	-0.33086300	-1.38669100
C	1.91814400	1.03800900	-1.32691200
O	1.33401800	2.20602000	-0.77168000
C	0.04275500	2.04003400	-0.20777100
C	0.08027600	1.93346000	1.26151000
C	0.12037100	1.85704500	2.46595900
H	0.15097500	1.78868800	3.53381100
H	-0.45407400	1.15629400	-0.62836800
H	-0.54992600	2.91995600	-0.47948100
H	2.71505300	1.39873300	-1.98406600
H	1.18138800	0.49680500	-1.93626900
H	1.28783000	-0.39620300	1.37198700
N	-0.07620800	-2.38157800	0.79933800
O	-0.50143400	-1.59499400	1.95707200
C	-1.43905800	-1.96217900	0.97020300
C	-2.06323600	-0.86014400	0.18290100
C	-2.18462700	-0.94498100	-1.20592500
C	-2.80743500	0.08505400	-1.90755600
C	-3.31941200	1.18742600	-1.22469100
C	-3.21605500	1.25945900	0.16389800
C	-2.59093100	0.23445300	0.86927800
H	-2.49741700	0.28652700	1.94973000
H	-3.61701500	2.11513600	0.69740200
H	-3.80177200	1.98920400	-1.77436100
H	-2.89251500	0.02548200	-2.98749800
H	-1.78350700	-1.80529000	-1.73148500
H	-2.10182000	-2.77309500	1.26593900

R_s***R***_TS1

O	-1.70246400	-1.34129800	-1.84486900
C	-1.89360900	-1.63528100	-0.66984000
N	-3.03841200	-1.47421900	0.00094000
C	-4.27074100	-0.87188400	-0.54215000
C	-4.80240600	-1.70201400	-1.71137300
H	-5.73470600	-1.26428700	-2.07853100

604	H	-4.08399100	-1.73588300	-2.53254200
605	H	-5.00659900	-2.72252800	-1.37784200
606	C	-5.28185300	-0.85772300	0.60173000
607	H	-5.49261200	-1.87873500	0.93245800
608	H	-6.21497200	-0.40444200	0.25911300
609	H	-4.90477500	-0.28217200	1.45129000
610	C	-4.00501600	0.55495400	-1.02986600
611	O	-3.49652300	1.33206400	0.04112800
612	C	-3.22192700	2.66307700	-0.36039700
613	C	-2.50902100	3.35391500	0.71931800
614	C	-1.88337900	3.91326000	1.58648300
615	H	-1.34032300	4.42176000	2.35685600
616	H	-4.15358900	3.19375500	-0.59885800
617	H	-2.59442600	2.66755100	-1.26393200
618	H	-3.28836700	0.55010500	-1.86186600
619	H	-4.95292700	0.97863900	-1.39075700
620	H	-3.04373300	-1.76543300	0.97115700
621	N	-0.84912900	-2.19047900	0.20721400
622	O	-0.75097000	-3.84696000	-0.71014700
623	C	0.17593900	-2.84956300	-0.58988100
624	C	1.43062200	-3.13191100	0.19571800
625	C	2.60174100	-2.43606300	-0.10385900
626	C	3.75876800	-2.65209500	0.64220100
627	C	3.74837300	-3.57892600	1.68305400
628	C	2.58133800	-4.28638400	1.97562500
629	C	1.42407300	-4.06229700	1.23481000
630	H	0.51085300	-4.60904900	1.45033900
631	H	2.57604000	-5.01223700	2.78274100
632	H	4.64826300	-3.75401200	2.26414000
633	H	4.66439100	-2.09952000	0.40924200
634	H	2.60673500	-1.72191600	-0.92428700
635	H	0.41735500	-2.31268900	-1.51907900
636	S	-0.03728600	-0.25535500	0.96641300
637	C	-0.26408200	1.03672900	-0.29837000
638	C	-1.31197000	0.17231200	2.17178600
639	C	0.97750600	1.11844100	-1.17422100
640	H	-0.45581500	1.97976100	0.22312900
641	H	-1.14584900	0.78830500	-0.89270900
642	H	-2.24640700	0.40408800	1.65968400
643	H	-1.43052900	-0.69139800	2.82836400
644	H	-0.97717200	1.03239900	2.75569500
645	C	2.14189400	1.83723300	-0.46442100
646	H	0.73033500	1.68140400	-2.08041200
647	H	1.29097100	0.11712100	-1.48966700

N	1.85849400	3.24208100	-0.27942400
H	2.33344900	1.36865700	0.50492000
C	3.40289100	1.66954000	-1.29664600
H	1.77886000	3.79944700	-1.12435800
C	1.52341100	3.87926700	0.86957800
O	3.90858100	2.53871900	-1.97203900
O	3.85767900	0.42146800	-1.21971400
O	1.17939900	5.06421400	0.84712400
C	1.61171100	3.10622400	2.16168200
C	5.00192400	0.11478100	-2.03296800
H	1.36887900	3.78253100	2.97972600
H	0.90798800	2.26828100	2.17061300
H	2.61756000	2.70305900	2.30610200
H	5.19444800	-0.94545600	-1.88112400
H	4.78007900	0.31737900	-3.08177700
H	5.85774100	0.71031700	-1.71136800

***R*_s*S*_TS1**

O	-0.00549200	-2.75687300	1.30268000
C	0.01748300	-1.53644600	1.22309200
N	-0.95069700	-0.69154300	1.59284200
C	-2.29850900	-1.07080500	2.06364600
C	-2.24428400	-2.14741800	3.14729700
C	-2.93477100	0.19571600	2.63215600
C	-3.11111700	-1.58501700	0.87367300
O	-3.12306500	-0.58367300	-0.13401400
C	-4.10440700	-0.78937300	-1.13352600
C	-3.92449800	-2.05059300	-1.87300100
C	-3.76742200	-3.08855200	-2.46803800
N	1.16853400	-0.81553200	0.63308500
O	2.02458200	-0.47652000	2.27484300
C	2.41040200	-1.27490200	1.22906300
C	3.62786800	-0.87590100	0.43336900
C	3.94999800	0.47094600	0.25832500
C	5.05620600	0.83305600	-0.50469100
C	5.85119000	-0.15138600	-1.09258000
C	5.53719400	-1.49701400	-0.91479700
C	4.42748900	-1.85723300	-0.15223200
S	1.08786100	-1.64285900	-1.45269400
C	0.84886000	0.03054300	-2.12625000
C	-0.52233100	-2.39791000	-1.78092200
C	-0.45756400	0.66999200	-1.64483800
C	-0.31085300	2.10623300	-1.12522100
N	0.69276400	2.18412900	-0.09023900

C	-1.67055700	2.51195300	-0.55523300
C	0.99462800	3.37492100	0.47587400
O	-1.89890100	2.70795200	0.61930400
O	-2.59903300	2.57654300	-1.50734600
O	0.54311000	4.43296200	0.02721900
C	1.92972400	3.33820000	1.65623900
C	-3.93096700	2.86969600	-1.05210000
H	-0.78818400	0.29537600	1.42077400
H	-3.24518500	-2.27075700	3.56930900
H	-1.90753100	-3.10783800	2.75776500
H	-1.56926300	-1.83457000	3.94833600
H	-3.95258800	-0.02837900	2.96051700
H	-2.36191300	0.55295500	3.49249200
H	-2.97573100	0.98767000	1.88120900
H	-2.66415900	-2.51001700	0.48210700
H	-4.13594000	-1.80338900	1.20517100
H	-5.10837800	-0.77346600	-0.68838200
H	-4.02121600	0.05472000	-1.82437500
H	-3.63227000	-4.00718900	-3.00090400
H	2.41667700	-2.35503900	1.43591900
H	3.33572100	1.23149600	0.72893000
H	5.30212900	1.88194900	-0.63732600
H	6.71564700	0.13100300	-1.68496200
H	6.15600200	-2.26539700	-1.36689100
H	4.17674300	-2.90547600	-0.00986800
H	1.73499300	0.57574300	-1.78784800
H	0.88991000	-0.04049100	-3.21483000
H	-0.70368700	-2.38468300	-2.85837900
H	-0.47510100	-3.42688600	-1.42317500
H	-1.32040200	-1.86336700	-1.26413300
H	-1.19269300	0.66905200	-2.45214700
H	-0.89473100	0.08808600	-0.83099900
H	-0.06482300	2.79559300	-1.93905400
H	1.00257200	1.31906200	0.35108700
H	1.59185400	4.07127600	2.39008200
H	1.97802300	2.34879600	2.11524700
H	2.93116100	3.62751800	1.32364200
H	-4.55725300	2.84962100	-1.94134300
H	-4.25528900	2.11185700	-0.33539900
H	-3.95725500	3.85617700	-0.58658400

S_sS_TS1

O	-1.38036100	-0.93107900	0.10181300
C	-1.79542800	0.15945600	0.48749400

736	N	-3.01279100	0.39437700	0.97815500
737	C	-4.13783900	-0.55292100	0.86457800
738	C	-3.93580700	-1.73413400	1.81140500
739	C	-5.39687100	0.22751600	1.23375800
740	C	-4.23896000	-1.06520000	-0.57881800
741	O	-4.18014400	0.04632300	-1.45868800
742	C	-3.92918200	-0.32058400	-2.80220800
743	C	-2.54380900	-0.78071100	-3.01613000
744	C	-1.40839900	-1.15401100	-3.18866900
745	N	-0.96248600	1.36751300	0.50527400
746	O	-1.50880700	1.87292300	-1.23957300
747	C	-0.27634700	1.50769100	-0.77533900
748	C	0.79778400	2.56483700	-0.76243800
749	C	2.06828500	2.28416000	-1.26410000
750	C	3.05692300	3.26851500	-1.26356800
751	C	2.77270200	4.53695600	-0.76565500
752	C	1.49712700	4.82489100	-0.27523700
753	C	0.51163500	3.84416600	-0.27895800
754	S	0.36641600	0.85241300	2.20526900
755	C	0.81353500	-0.90793900	2.44582800
756	C	-0.93687800	0.98745200	3.44935900
757	C	2.19778500	-1.21672400	1.88840500
758	C	2.27945300	-0.97452400	0.38114400
759	N	1.33972400	-1.78464100	-0.36925400
760	C	3.66718800	-1.13640300	-0.22781100
761	C	1.57762200	-3.09844000	-0.57199000
762	O	3.87769600	-1.04485200	-1.41984600
763	O	4.62121700	-1.32006500	0.67804400
764	O	2.66180200	-3.61340500	-0.27375500
765	C	0.46918500	-3.87560000	-1.23120100
766	C	5.95271500	-1.44886000	0.15449000
767	H	-3.23483400	1.36793200	1.14909700
768	H	-4.74474200	-2.45818300	1.67699400
769	H	-2.98433200	-2.23429000	1.61602500
770	H	-3.94996900	-1.38842700	2.84852900
771	H	-6.26020700	-0.44146200	1.21149500
772	H	-5.30564100	0.64379400	2.24109200
773	H	-5.56795700	1.04438900	0.52717700
774	H	-3.42182500	-1.76161700	-0.79470300
775	H	-5.19259800	-1.59829700	-0.69767700
776	H	-4.62638800	-1.10254800	-3.13038700
777	H	-4.11108300	0.57054200	-3.40818700
778	H	-0.40057300	-1.47967700	-3.34758500
779	H	0.11316500	0.56346700	-1.18673700

H	2.28461400	1.29412200	-1.66134900
H	4.04418600	3.04349400	-1.65409900
H	3.54039200	5.30422200	-0.76291000
H	1.27385500	5.81641900	0.10566900
H	-0.48650900	4.06266200	0.08902000
H	0.79307700	-1.07337100	3.52623600
H	0.03200700	-1.51842200	1.98289900
H	-0.47932700	0.98970100	4.43951600
H	-1.45764200	1.93004100	3.27731500
H	-1.63215900	0.15075500	3.35437500
H	2.94765500	-0.60353300	2.39448800
H	2.42447000	-2.26633900	2.09944000
H	2.02928100	0.07447500	0.17694000
H	0.38630100	-1.43693900	-0.45521800
H	-0.48996900	-3.35678300	-1.17744100
H	0.39173000	-4.85309000	-0.75306300
H	0.73082300	-4.03085400	-2.28181700
H	6.00711300	-2.31436600	-0.50808600
H	6.23073000	-0.54540600	-0.39033300
H	6.59683400	-1.58805100	1.01974700

***S*_s*R*_TS1**

O	1.25219900	-1.84412600	-1.23755600
C	1.94150300	-1.66875100	-0.23523600
N	3.17798300	-1.16452300	-0.24505900
C	3.83689300	-0.59737500	-1.43333000
C	5.17202300	-0.04084700	-0.94190300
C	4.07186800	-1.67001000	-2.49773700
C	2.99347500	0.52816900	-2.03582900
O	2.78295900	1.53128700	-1.05458700
C	2.17333200	2.69068700	-1.58510600
C	0.75745900	2.48012900	-1.93308100
C	-0.40109100	2.31033900	-2.22744100
N	1.42620800	-2.12986900	1.06459400
O	2.87937300	-1.85947700	2.25524500
C	1.65969000	-1.25468100	2.21801800
C	1.66984200	0.25010000	2.03564300
C	2.84993200	0.95903800	2.26281500
C	2.86990100	2.34779000	2.14804300
C	1.70976500	3.03573400	1.79749900
C	0.52766700	2.33152000	1.56922700
C	0.50615300	0.94520100	1.70349600
S	-0.81439000	-2.40840000	0.79408100
C	-1.68580400	-1.87101100	-0.71624200

824	C	-0.51950600	-4.12414900	0.32718500
825	C	-2.05385100	-0.39769400	-0.67347500
826	C	-3.07257200	-0.06004500	0.42790300
827	N	-4.26910700	-0.87167700	0.36222200
828	C	-3.45601300	1.41845700	0.45400900
829	C	-5.15497200	-0.73168800	-0.64730900
830	O	-4.49990700	1.84945800	0.89314000
831	O	-2.47734600	2.19021000	-0.00753300
832	O	-4.99571400	0.11660000	-1.53185700
833	C	-6.34498600	-1.65292300	-0.63394100
834	C	-2.72293400	3.60467300	0.02053900
835	H	3.64474500	-1.10940400	0.65442500
836	H	5.70848300	0.41965700	-1.77515300
837	H	5.01607200	0.71339800	-0.16572900
838	H	5.78968900	-0.84585400	-0.53334200
839	H	4.60966800	-1.23626500	-3.34548000
840	H	4.67872000	-2.47796400	-2.08089000
841	H	3.12940100	-2.08667700	-2.85708600
842	H	3.54293800	0.94993800	-2.88980900
843	H	2.03297800	0.14275900	-2.39735900
844	H	2.24634200	3.46492500	-0.81678300
845	H	2.71618600	3.03809900	-2.47472600
846	H	-1.42987300	2.16813700	-2.48732600
847	H	0.95593000	-1.54151400	3.01463000
848	H	3.75042700	0.41639500	2.53347000
849	H	3.79142000	2.89133200	2.33130800
850	H	1.72355200	4.11733100	1.70558000
851	H	-0.38118400	2.86203100	1.30394400
852	H	-0.42712200	0.40620500	1.56900700
853	H	-1.02077600	-2.07499200	-1.55510000
854	H	-2.57491400	-2.50461400	-0.79805600
855	H	-1.47475100	-4.61406800	0.13186600
856	H	0.11518500	-4.14454500	-0.56060500
857	H	-0.01789300	-4.61401400	1.16091700
858	H	-1.15152800	0.20676300	-0.54182500
859	H	-2.48217200	-0.13088100	-1.64369800
860	H	-2.62796200	-0.24710100	1.41326900
861	H	-4.42549100	-1.57549200	1.07013400
862	H	-6.33748100	-2.23955600	-1.55538200
863	H	-6.35389100	-2.32465200	0.22475300
864	H	-7.25201100	-1.04454300	-0.63178600
865	H	-1.83086800	4.06473400	-0.40130200
866	H	-3.59922500	3.84206000	-0.58487300
867	H	-2.87908600	3.93766300	1.04809400

R_sR_int1

O	2.06926000	0.23751300	1.64108500
C	1.97518900	-0.55538100	0.70616000
N	2.95900900	-1.25204100	0.13612100
C	4.37455200	-1.22545400	0.53389100
C	4.59918200	-2.13411400	1.74266300
H	5.63879600	-2.06827400	2.07740300
H	3.94638300	-1.83561700	2.56814800
H	4.38364600	-3.17292900	1.47965400
C	5.16501100	-1.71652500	-0.67768100
H	4.81508900	-2.70615300	-0.98683400
H	6.22498900	-1.78847000	-0.42246200
H	5.05070200	-1.02516400	-1.51761500
C	4.81855300	0.19372900	0.91106300
O	4.34697200	1.11577100	-0.05742300
C	4.31073300	2.43874200	0.44958500
C	3.64322600	3.30485200	-0.52661700
C	3.06898200	4.00747800	-1.32107200
H	2.56882200	4.63501800	-2.02974600
H	5.32560700	2.80889900	0.64834500
H	3.75182500	2.46432600	1.39567400
H	4.43145600	0.46093600	1.89860300
H	5.91674700	0.21092800	0.95199700
H	2.67179700	-1.81289000	-0.67873800
N	0.67267800	-0.79406100	0.10641100
O	1.12596800	-2.40526300	-1.61464900
C	0.45395700	-2.22519600	-0.48387000
C	-1.06542400	-2.40726300	-0.55911300
C	-1.86112300	-2.23496800	0.57862700
C	-3.23748700	-2.43625000	0.51793700
C	-3.83119100	-2.83079700	-0.68317900
C	-3.04321800	-3.00791500	-1.81802800
C	-1.66524900	-2.79322100	-1.75548000
H	-1.03820900	-2.93239300	-2.62990300
H	-3.50086400	-3.31264100	-2.75424900
H	-4.90319200	-2.99485300	-0.73228200
H	-3.84534800	-2.29084700	1.40719600
H	-1.39737900	-1.93137400	1.51437800
H	0.80541000	-2.82446200	0.38463700
S	0.23694900	0.35775100	-1.03805300
C	0.07796000	1.87614600	-0.05002200
C	1.67975900	0.75380600	-2.04037200

C	-0.96335700	1.69896900	1.04562300
H	-0.20088800	2.64291500	-0.77875100
H	1.06190800	2.10971500	0.36268100
H	2.53137300	0.99820200	-1.40241800
H	1.87363500	-0.12766600	-2.65075400
H	1.39841300	1.60702400	-2.66291000
C	-2.32809800	1.25665400	0.50614400
H	-1.06658200	2.66130800	1.55774700
H	-0.60754800	0.96814100	1.77359500
N	-2.82644600	2.20231800	-0.46212700
H	-2.22017900	0.27490900	0.02726900
C	-3.33958900	1.01371300	1.62485100
H	-2.70972000	3.18661900	-0.23997100
C	-3.79011000	1.93717800	-1.38756100
O	-4.47422200	1.43949300	1.62440700
O	-2.84085800	0.22615500	2.57282000
O	-4.32916100	2.85447100	-2.00721500
C	-4.12281200	0.48987300	-1.63822200
C	-3.75344200	-0.14399200	3.62061200
H	-4.56821800	0.04007400	-0.74572900
H	-4.83190300	0.43449600	-2.46280500
H	-3.22322600	-0.08158800	-1.88915000
H	-3.20084500	-0.82510100	4.26379300
H	-4.06209100	0.74314900	4.17571800
H	-4.62798100	-0.63928100	3.19565800

S_s***R***_int1

O	0.94485200	-1.48844500	-1.60894300
C	1.32904600	-1.39063900	-0.43992800
N	2.59595000	-1.36779100	-0.01783700
C	3.76559700	-1.13362100	-0.87598000
C	4.94164600	-0.88167300	0.06528700
C	4.03787100	-2.34558000	-1.76774600
C	3.53241200	0.09023200	-1.76212100
O	3.20563900	1.20888600	-0.94998500
C	2.72405300	2.29412200	-1.72107600
C	1.34575600	2.06226000	-2.18981400
C	0.21622900	1.84897200	-2.56041900
N	0.37889000	-1.29282300	0.62143400
O	1.72287300	-1.69768700	2.58267500
C	0.85274200	-0.85319100	2.06178200
C	1.33726300	0.59590100	1.88557400
C	2.53058900	0.98748500	2.48886400
C	2.97894500	2.30412000	2.38285200

956	C	2.22691400	3.24559600	1.68282600
957	C	1.02841400	2.85987300	1.08070300
958	C	0.59124500	1.54195700	1.17969900
959	S	-0.85193700	-2.40535100	0.63460100
960	C	-1.90468700	-2.03411000	-0.81043600
961	C	-0.13160200	-3.98110900	0.15354500
962	C	-2.16356800	-0.54347200	-0.92830800
963	C	-2.88548500	0.05861400	0.28661100
964	N	-4.10407000	-0.63427900	0.63864400
965	C	-3.15904400	1.55435800	0.11815500
966	C	-5.19184100	-0.57502100	-0.16275500
967	O	-4.06022400	2.14994200	0.66552500
968	O	-2.24348100	2.14028200	-0.64774100
969	O	-5.16841900	0.04166300	-1.23232800
970	C	-6.42466700	-1.28817700	0.32160300
971	C	-2.34560300	3.56767300	-0.77304400
972	H	2.71644700	-1.44843700	0.99981000
973	H	5.85274200	-0.72092700	-0.51642400
974	H	4.75810600	0.00073100	0.68490000
975	H	5.09579300	-1.74507800	0.71957300
976	H	4.90559400	-2.15466500	-2.40587900
977	H	4.24906700	-3.22224000	-1.14976300
978	H	3.17609100	-2.56246700	-2.40317100
979	H	4.45120200	0.29382800	-2.33019600
980	H	2.72215000	-0.10748000	-2.47306800
981	H	2.74723500	3.17779700	-1.07873700
982	H	3.37833800	2.47992900	-2.58303200
983	H	-0.78502600	1.67055000	-2.89570400
984	H	-0.13169500	-0.80386400	2.57808400
985	H	3.10532200	0.24716200	3.03568200
986	H	3.91356400	2.59574900	2.85244100
987	H	2.57291500	4.27144800	1.60219200
988	H	0.44057600	3.58122100	0.51860700
989	H	-0.33020700	1.24580400	0.68872400
990	H	-1.38934100	-2.41534000	-1.69211400
991	H	-2.81820800	-2.60921200	-0.62991700
992	H	-0.94717900	-4.70604400	0.10724000
993	H	0.35665600	-3.87855300	-0.81661500
994	H	0.57771200	-4.25056800	0.93721600
995	H	-1.21138000	-0.02838600	-1.07507800
996	H	-2.77012100	-0.39292800	-1.82474300
997	H	-2.22632500	0.00353400	1.16444700
998	H	-4.17161300	-1.09148900	1.53727600
999	H	-7.21071600	-0.54561600	0.47870900

H	-6.76046700	-1.97167400	-0.46077700
H	-6.25777900	-1.84072800	1.24627700
H	-1.51084300	3.86571200	-1.40488900
H	-3.29532100	3.83350900	-1.23994800
H	-2.27093900	4.03404800	0.21071600

R_sS_int1

O	0.37677200	-2.71268700	-0.43469100
C	0.70061100	-1.74166500	0.25063600
N	0.09065800	-1.29178100	1.35183600
C	-0.96108100	-1.99208000	2.10654000
C	-0.32274600	-3.03159700	3.03030300
H	-1.09513500	-3.59879900	3.55837300
H	0.28914200	-3.72844600	2.44967200
H	0.31269000	-2.53751900	3.77018900
C	-1.70625000	-0.93130700	2.91735200
H	-1.00512500	-0.34175100	3.51742500
H	-2.41161200	-1.42176500	3.59315900
H	-2.26249800	-0.25366500	2.26182800
C	-1.94020500	-2.71723800	1.17916100
O	-2.34685900	-1.85031600	0.13274800
C	-2.96150000	-2.56965100	-0.92327300
C	-3.21577200	-1.65946800	-2.04218400
C	-3.41371600	-0.91454700	-2.97012500
H	-3.59911300	-0.24397600	-3.78387500
H	-3.90392600	-3.02286300	-0.58687800
H	-2.29930600	-3.38011100	-1.25888800
H	-1.47270200	-3.61144200	0.75707300
H	-2.81058500	-3.02712900	1.77492400
H	0.48553700	-0.41675000	1.73467200
N	1.83458600	-0.94533700	-0.13446300
O	1.86629700	0.78163300	1.58400400
C	2.60088000	-0.15415400	1.00856100
C	3.90396400	0.31229900	0.36176600
C	4.81311800	-0.60815300	-0.16827300
C	5.99406500	-0.16947400	-0.75980000
C	6.27761100	1.19583000	-0.82329500
C	5.37741200	2.11506100	-0.28999500
C	4.19443700	1.67239400	0.30128900
H	3.48280500	2.37344900	0.72470200
H	5.59603900	3.17755600	-0.33388000
H	7.19873100	1.53879100	-1.28370600

H	6.69526300	-0.88993400	-1.16919500
H	4.59187700	-1.67164200	-0.11547800
H	2.83670700	-1.02734100	1.65307900
S	1.73600500	-0.22443900	-1.63164400
C	0.00099900	0.30494000	-1.79433200
C	1.83417700	-1.55315400	-2.84112400
C	-0.23405500	1.45150800	-0.81051200
H	-0.62329000	-0.57233500	-1.58944500
H	-0.12540700	0.58887200	-2.84273800
H	1.85084100	-1.07615700	-3.82324700
H	2.77671100	-2.07142800	-2.65774300
H	0.98215800	-2.22131700	-2.72902600
C	-1.67245200	1.46216300	-0.29115100
H	-0.00122400	2.41057900	-1.28382600
H	0.42908200	1.33984100	0.05538200
N	-2.59392700	1.85227200	-1.32947100
H	-1.90550500	0.44540400	0.05410400
C	-1.79562100	2.35666900	0.94166700
H	-2.23619900	2.41309100	-2.09494400
C	-3.93236100	1.64000000	-1.33210200
O	-2.60786700	3.24469500	1.08633400
O	-0.91270800	1.99879600	1.86800800
O	-4.62927300	2.05893000	-2.26163000
C	-4.52592200	0.89265200	-0.16585000
C	-0.98675500	2.68394600	3.12587600
H	-5.55905800	0.64721000	-0.40845200
H	-3.97138100	-0.02364300	0.05265700
H	-4.50566400	1.52611800	0.72609100
H	-0.16398600	2.29065100	3.71978000
H	-0.87091100	3.75848800	2.97683400
H	-1.94417700	2.47466400	3.60734900

***S*_s*S*_int1**

O	1.48000700	1.38719000	0.84636900
C	1.76368400	0.20597500	0.62647100
N	2.97967800	-0.29138100	0.39009300
C	4.16014500	0.50324000	0.01226600
C	4.55440500	1.47874200	1.12047700
C	5.28929700	-0.49354800	-0.23923500
C	3.83435700	1.27734200	-1.26537400
O	3.47830100	0.34860300	-2.27927300
C	2.69945000	0.92588900	-3.30917900
C	1.32762500	1.23116200	-2.86454900
C	0.20357100	1.48470300	-2.50112700

1088	N	0.73285000	-0.79344100	0.62899900
1089	O	1.88438500	-2.88276800	0.35032700
1090	C	1.00273300	-2.08754800	-0.22937200
1091	C	-0.39135800	-2.65854300	-0.48828700
1092	C	-1.26918300	-2.00157100	-1.35596900
1093	C	-2.56053200	-2.48336600	-1.55244000
1094	C	-2.98183700	-3.63508900	-0.88488400
1095	C	-2.10303600	-4.30533500	-0.03631300
1096	C	-0.81015100	-3.81907100	0.15790800
1097	S	0.11100800	-1.17092500	2.13651000
1098	C	-1.00165700	0.19982000	2.55641500
1099	C	1.44516500	-0.91973500	3.31685600
1100	C	-2.18791800	0.26897900	1.59989300
1101	C	-1.84606000	0.96318400	0.27676400
1102	N	-1.32580700	2.30173000	0.45868000
1103	C	-3.00080100	0.99925400	-0.71639400
1104	C	-2.14327700	3.31724200	0.80497000
1105	O	-2.96215000	1.62912000	-1.75542800
1106	O	-4.01851400	0.22224400	-0.36873000
1107	O	-3.36693000	3.15322400	0.88996400
1108	C	-1.49544500	4.64979000	1.06329800
1109	C	-5.11042100	0.16978200	-1.30088700
1110	H	3.01877400	-1.31624300	0.32018500
1111	H	5.47303000	2.00038800	0.83783700
1112	H	3.77219400	2.21784100	1.30003800
1113	H	4.74037300	0.92985200	2.04775600
1114	H	6.18609400	0.04151700	-0.56095100
1115	H	5.52144500	-1.04088200	0.67884300
1116	H	5.01150200	-1.21090100	-1.01566700
1117	H	3.00305800	1.96728300	-1.07345300
1118	H	4.70995900	1.86296300	-1.57712200
1119	H	3.17026600	1.84098500	-3.69132000
1120	H	2.66438400	0.19838100	-4.12394500
1121	H	-0.80689600	1.72717700	-2.22708900
1122	H	1.33453000	-1.58717000	-1.16726000
1123	H	-0.93155800	-1.11039100	-1.88472800
1124	H	-3.23490100	-1.96839900	-2.23065400
1125	H	-3.98847600	-4.01239100	-1.03504500
1126	H	-2.42467500	-5.20731900	0.47513200
1127	H	-0.11126400	-4.32965000	0.81347000
1128	H	-1.33231300	-0.04514500	3.57032600
1129	H	-0.41177900	1.11947000	2.58278500
1130	H	1.02787000	-1.09516400	4.31053100
1131	H	2.20713500	-1.66517400	3.08701400

H	1.83447600	0.09734100	3.23587700
H	-2.57520400	-0.73507700	1.39690000
H	-2.98156100	0.82869200	2.10208900
H	-1.05916600	0.39625500	-0.22892000
H	-0.31631200	2.40857600	0.46623600
H	-0.41608300	4.62864500	0.91145600
H	-1.71434600	4.94748900	2.09136200
H	-1.94528800	5.38931200	0.39728300
H	-5.58400800	1.15076800	-1.36817800
H	-4.75281400	-0.13704200	-2.28479100
H	-5.80494400	-0.56423300	-0.89823300

s*R*_sulfilimine

O	-3.81013000	0.41887100	0.83918100
C	-2.81168400	0.15727500	0.12299800
N	-2.87272400	-0.93261700	-0.70422800
C	-1.77637600	-1.63198900	-1.38794800
C	-2.35953600	-2.95321600	-1.89179300
H	-1.59964100	-3.50193300	-2.45438900
H	-2.69566800	-3.57648100	-1.05943700
H	-3.20842400	-2.76125300	-2.55417700
C	-1.27756400	-0.81499400	-2.57948000
H	-1.04770700	0.21073700	-2.29310800
H	-2.05207900	-0.78470600	-3.35137400
H	-0.37833000	-1.26666400	-3.01000600
C	-0.60726400	-1.92913300	-0.44247100
O	-1.08719900	-2.68674800	0.65904400
C	-0.05138100	-3.25925600	1.43067800
C	0.66151700	-2.27883600	2.26878500
C	1.25135300	-1.50165600	2.98017300
H	1.77362300	-0.81371400	3.61282400
H	0.67372800	-3.77390100	0.78424200
H	-0.51723400	-4.00693400	2.07829100
H	0.14828600	-2.50975500	-0.99310400
H	-0.14047600	-1.00225700	-0.08895500
H	-3.69696400	-1.48906400	-0.51917000
N	-1.68083900	0.91982600	0.07200400
S	-1.74611100	2.05211200	1.28583100
C	-0.04089000	2.66384400	1.23386000
C	-2.59558000	3.48075100	0.59219000
C	0.93906900	1.50903800	1.47281300
H	0.01625300	3.42259200	2.01714300

H	0.10255800	3.14787000	0.26468900
H	-2.18106200	3.68855000	-0.39579000
H	-3.65069100	3.21468400	0.52541200
H	-2.45900200	4.32916900	1.26607800
C	1.92905200	1.33489500	0.30816700
H	0.39904500	0.56558800	1.60468100
H	1.51599000	1.68023600	2.38282000
N	1.22296000	1.25802700	-0.94954800
H	2.61253800	2.19050900	0.30296000
C	2.79531200	0.11527000	0.61722000
H	0.22707900	1.03049500	-0.90893500
C	1.78437400	1.43245200	-2.16961700
O	3.59896500	0.10988400	1.52791800
O	2.57491900	-0.91831500	-0.18514000
O	1.11013600	1.35245900	-3.20107200
C	3.25868600	1.74754400	-2.21384900
C	3.36615000	-2.09033600	0.07272300
H	3.84805500	0.97733200	-1.70817500
H	3.56487500	1.80852600	-3.25684000
H	3.45841600	2.70392800	-1.72185500
H	3.03872800	-2.83038100	-0.65468500
H	4.42394300	-1.85968600	-0.06633200
H	3.19119700	-2.44306500	1.09015800

s*S*_sulfilimine

O	1.75718200	1.92189900	1.26556400
C	1.44110200	1.67706000	0.08423000
N	2.22861600	0.93186200	-0.74803100
C	3.43851000	0.21413300	-0.32452600
C	3.99910600	-0.46290500	-1.57578400
H	4.86358900	-1.07728400	-1.31039700
H	3.24777900	-1.10360700	-2.04466000
H	4.31744600	0.29239300	-2.29987400
C	4.48465300	1.18007700	0.23734700
H	4.16838600	1.60961100	1.18842900
H	4.65407400	1.99231100	-0.47470200
H	5.42987300	0.65064900	0.38732300
C	3.12222100	-0.83661900	0.74184400
O	2.32630900	-1.86798100	0.17323500
C	1.97023600	-2.85445200	1.12012900
C	0.94429500	-2.37753200	2.06491500
C	0.10488700	-1.97661900	2.83496900
H	-0.63599300	-1.62823400	3.52536100
H	2.85285100	-3.18864500	1.68215300

H	1.58239500	-3.71007800	0.56071400
H	4.06163100	-1.26773000	1.11837700
H	2.59222700	-0.36718700	1.57984500
H	1.76589100	0.56549400	-1.57466800
N	0.29886700	2.11201600	-0.55876700
S	-0.71583000	2.93247000	0.44515700
C	-2.14214300	1.80749300	0.64118300
C	-1.45824400	4.10891200	-0.68822800
C	-1.71614100	0.46513900	1.25945800
H	-2.58509200	1.69512300	-0.35499300
H	-2.85291000	2.34093400	1.27691600
H	-2.32837300	4.55074600	-0.19920800
H	-0.71417700	4.87739800	-0.89825100
H	-1.74290700	3.58407800	-1.60133300
C	-1.76977600	-0.70883800	0.26925600
H	-2.37490500	0.23578200	2.09769900
H	-0.69568100	0.50987300	1.65796700
N	-0.76465000	-0.55288300	-0.75167400
H	-1.59260100	-1.62568400	0.84212600
C	-3.16079800	-0.85121000	-0.34605100
H	-0.45104900	0.40596300	-0.94901400
C	-0.21789500	-1.51877800	-1.51509800
O	-3.38831600	-0.94807900	-1.53268500
O	-4.10575600	-0.87439700	0.59287500
O	0.60838000	-1.22700900	-2.39286900
C	-0.65241900	-2.94097400	-1.27586100
C	-5.45219500	-1.03260300	0.11530500
H	-0.00606600	-3.60496100	-1.84827200
H	-0.60412400	-3.20303100	-0.21546000
H	-1.68812200	-3.06898400	-1.60512400
H	-6.08094000	-1.03396500	1.00282500
H	-5.71568700	-0.20083800	-0.53982100
H	-5.54885200	-1.97577100	-0.42464900

Benzaldehyde

C	2.20777400	-0.24645000	0.00000200
C	1.72658000	1.06171400	0.00000200
C	0.35390400	1.29068900	-0.00000100
C	-0.53047800	0.20962300	-0.00000300
C	-0.04595800	-1.10329700	-0.00000300
C	1.32366900	-1.32888600	0.00000000
H	3.27808200	-0.42668100	0.00000400
H	2.41862600	1.89699900	0.00000400
H	-0.03922800	2.30397500	-0.00000100

H -0.75056700 -1.92919300 -0.00000500
H 1.70853500 -2.34316800 0.00000000
C -1.98149200 0.47348100 -0.00000600
O -2.83386900 -0.39800400 0.00000600
H -2.26850100 1.54086200 0.00000100

Table S1.

Proteomic data containing shotgun proteomics data of proteins with evidence for stereospecific MetO reduction treated with (*R*)- or (*S*)-ChURRO-2, identified prochiral Met oxidation sites in oxidatively-stressed HEK-293T cells with MsrA or MsrB2 knocked down, quantitative isoDTB proteomic data in HEK-293T cell lysates for (*R*)- or (*S*)-ChURRO-2, identified prochiral Met oxidation sites in purified mitochondria derived from mouse liver, isoTOP-ABPP hyper-reactivity data of Met sites in purified mitochondria derived from mouse liver, BPHL shotgun proteomics with ChURRO-2, and ^{18}O-H_2O_2 proteomics shotgun proteomics with BPHL, and isoTOP-ABPP hyper-reactivity of *N*-Hcylated sites in HEK-293T cells.

Table S2.

Quantification data containing protein labeling data for all 5 ChURRO-probes determined by densitomtry, cell viability data for live cell labeling of ChURRO-2 in HEK-293T cells and densitometry quantification of dot blot to assess probe internalization, Michaelis-Menten kinetic data for all purified hBPHL variants, relative activity of WT hBPHL oxidative stress treated with (*R*)- or (*S*)-ChURRO-2 and (*R*)- or (*S*)-OT-Ox used to compute dose-response curves and IC_{50} values, peptide spectrum matches for shotgun proteomics analysis of WT hBPHL treated with racemic (*R*)- or (*S*)-ChURRO-2, densitometry quantification of BPHL expression in a panel of renal and hepatic cell lines, SOD1 cellular activity assays, densitometry quantification of protein *N*-homocysteinylation with MsrA or MsrB2 KO, and densitometry quantification of BPHL Co-IP with MsrA or MsrB2.

Table S3.

Bioinformatic data containing druggability analysis identified stereoselective Met oxidation sites for all ChURRO-2 probe pairs according to ChEMBL database, extracted phi and psi angles and Ramachandran analysis of identified Met oxidation sites for ChURRO-2 probe pairs, crowdedness analysis of identified Met oxidation sites for ChURRO-2 probe pairs by predicted partial sphere exposure analysis, subcellular distribution of identified prochiral Met oxidation sites in purified mitochondria derived from mouse liver, AlphaMissense analysis of identified prochiral Met oxidation sites in purified mitochondria derived from mouse liver, and gene ontology analysis of identified prochiral Met oxidation sites in purified mitochondria derived from mouse liver.

1308

NMR Spectra

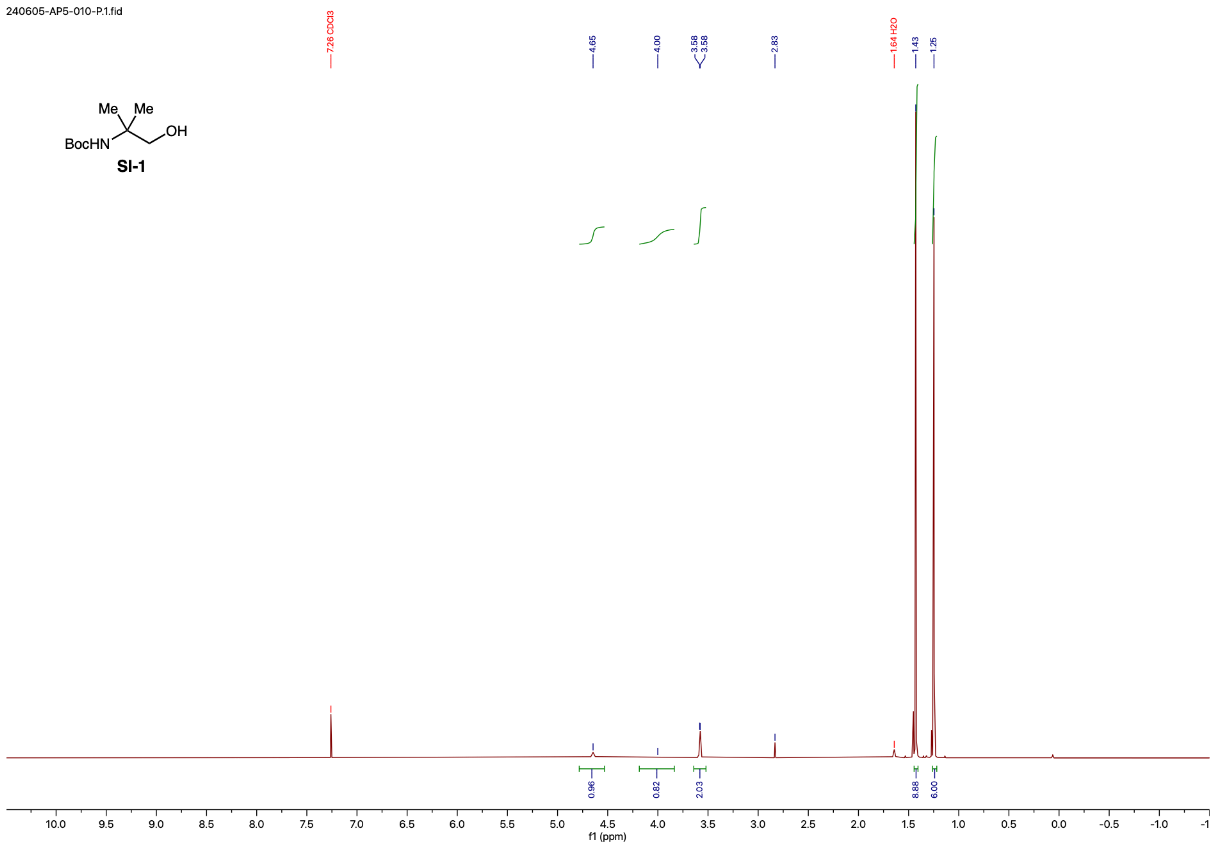
240605-AP5-010-P.1.fid
7.26 CDCl3
4.65
4.00
3.58
3.58
2.83
1.64 H2O
1.43
1.25
Me
Me
BocHN
OH
SI-1
0.96
0.82
2.03
8.88
6.00
f1 (ppm)

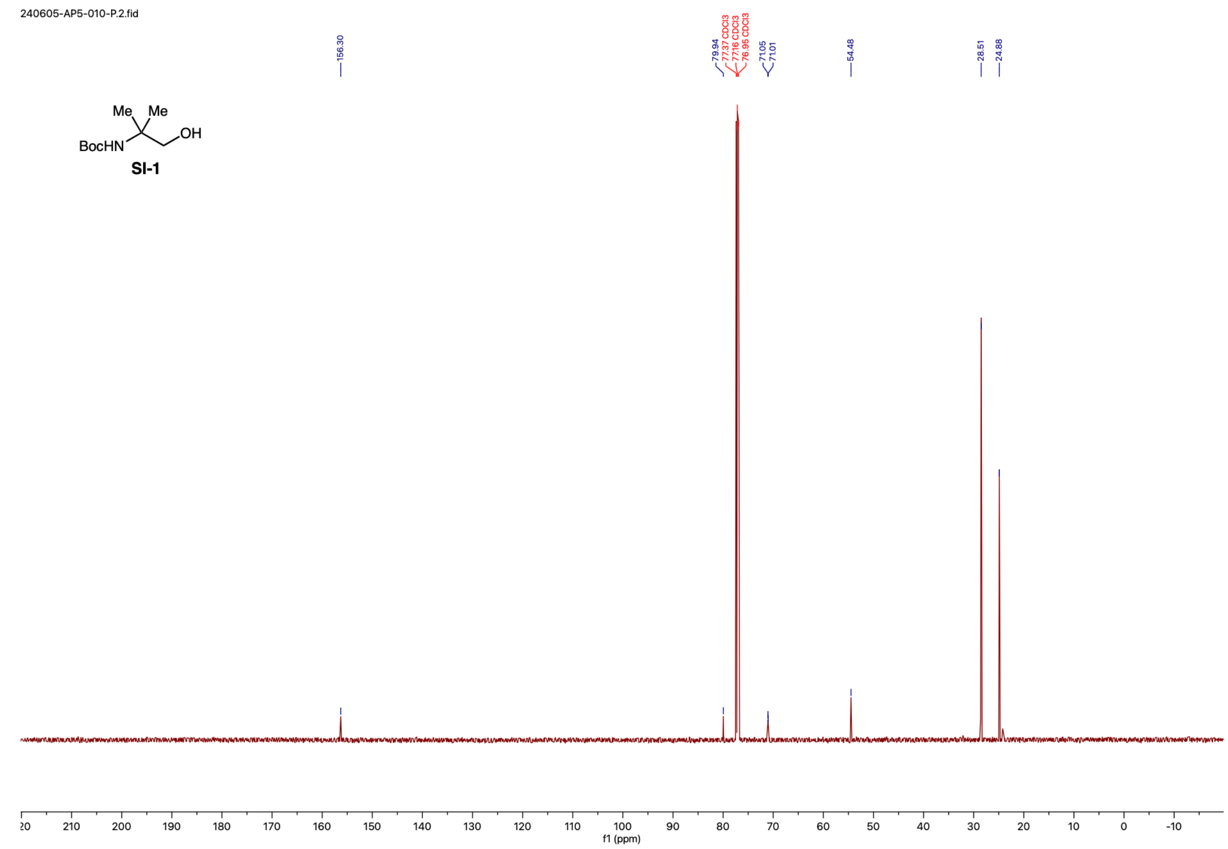
240605-AP5-010-P.2.fid
156.30
79.94
77.37 CDCl3
77.16 CDCl3
76.95 CDCl3
71.05
71.01
54.48
28.51
24.88
Me
Me
BocHN
OH
SI-1
f1 (ppm)

1312

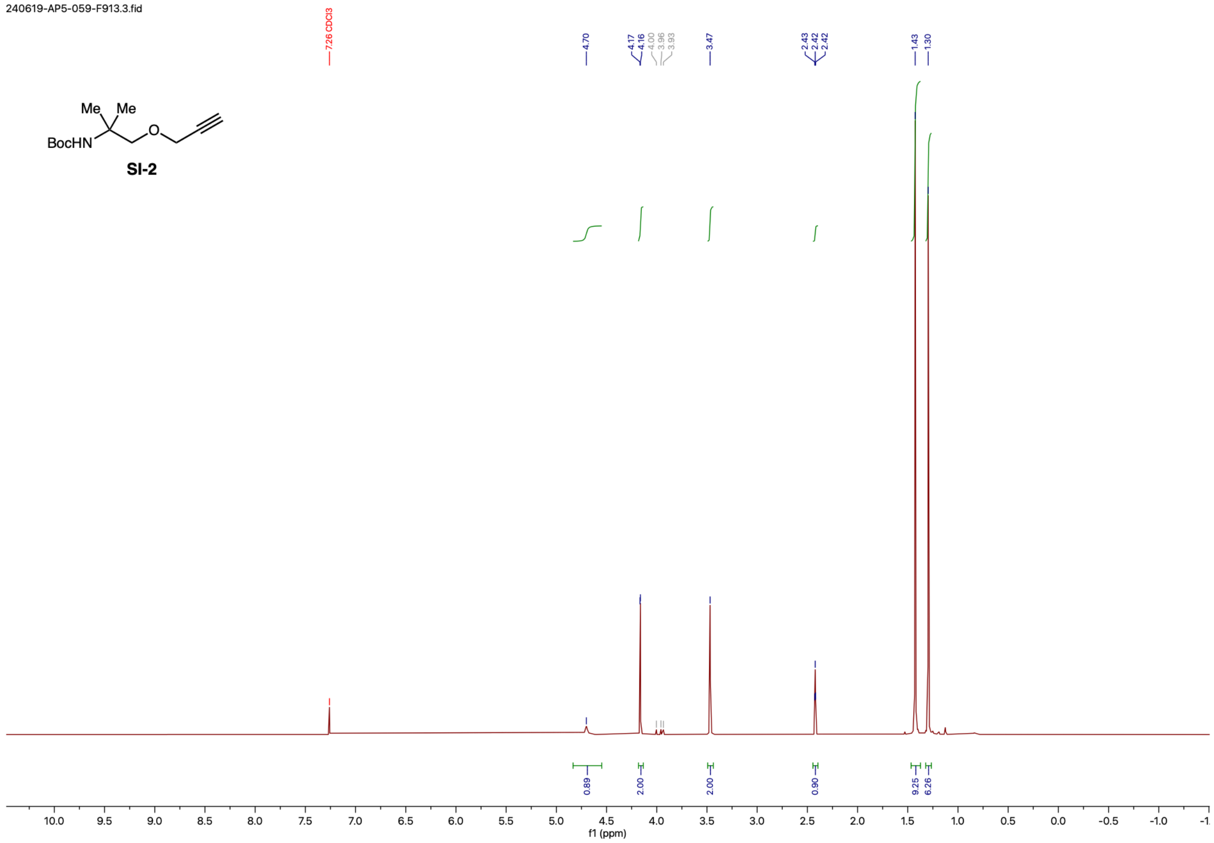
240619-AP5-059-F913.3.fid
7.26 CDCl3
4.70
4.17
4.16
4.00
3.96
3.93
3.47
2.43
2.42
2.42
1.43
1.30
Me
Me
BocHN
O
SI-2
0.89
2.00
2.00
0.90
9.25
6.26
10.0 9.5 9.0 8.5 8.0 7.5 7.0 6.5 6.0 5.5 5.0 4.5 4.0 3.5 3.0 2.5 2.0 1.5 1.0 0.5 0.0 -0.5 -1.0 -1
f1 (ppm)

1313

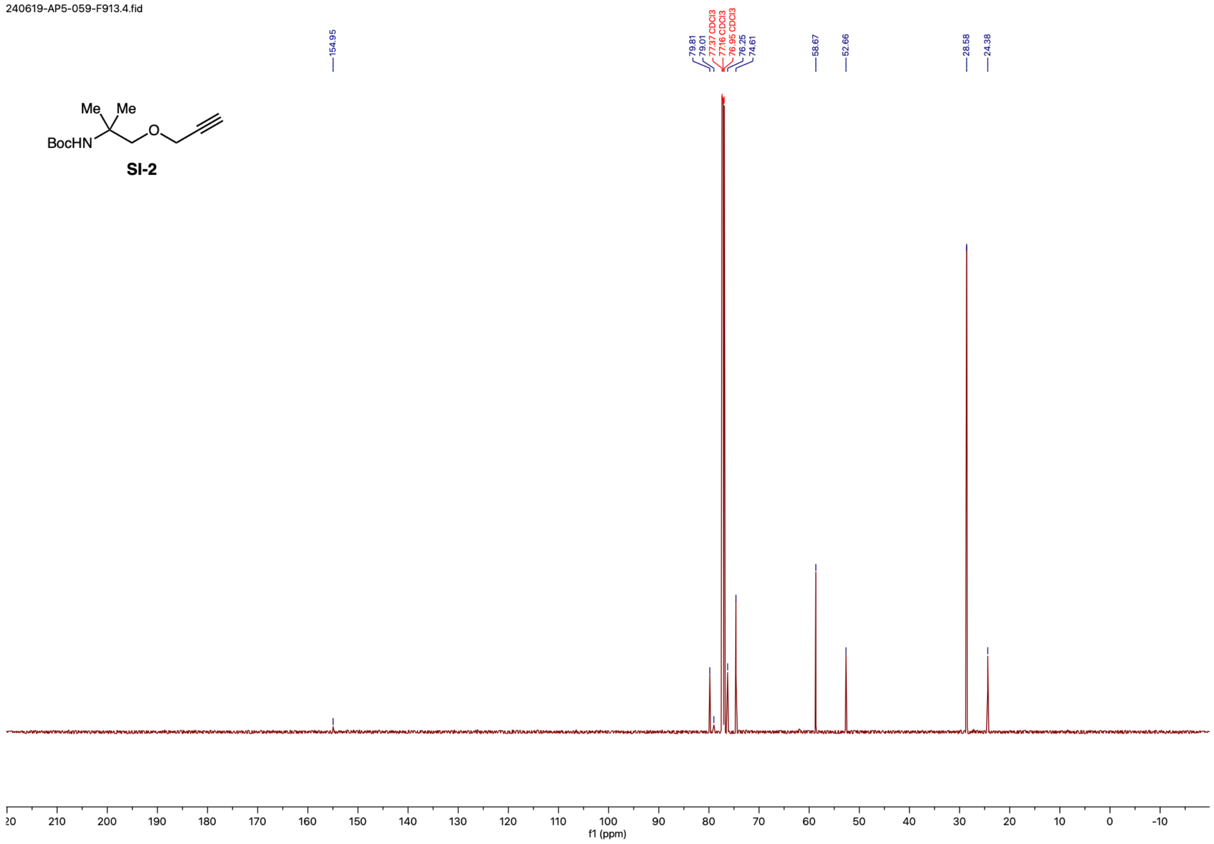
240619-AP5-059-F913.4.fid
154.95
79.81
79.01
77.37 CDCl3
77.16 CDCl3
76.95 CDCl3
76.25
74.61
58.67
52.66
28.58
24.38
Me
Me
BocHN
O
SI-2
210
200
190
180
170
160
150
140
130
120
110
100
90
80
70
60
50
40
30
20
10
0
-10
f1 (ppm)

1314

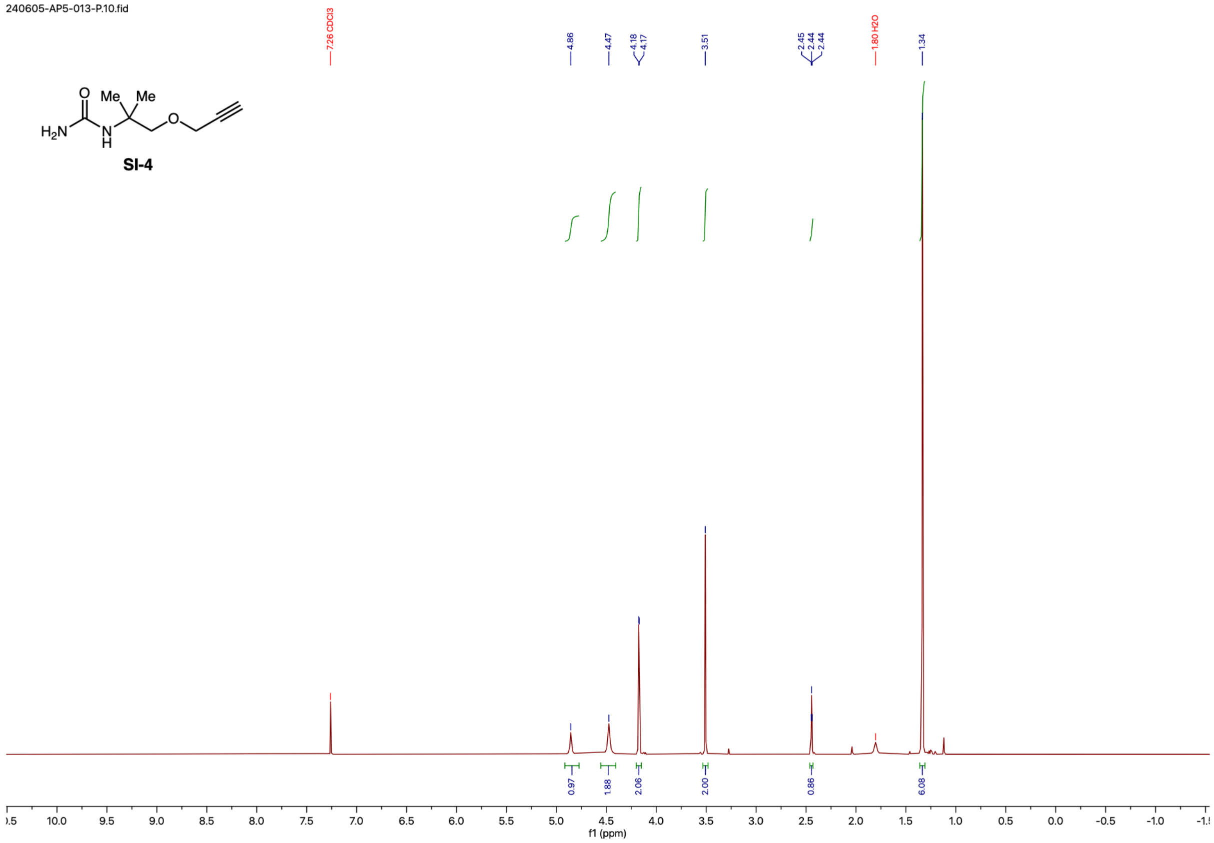

240605-AP5-013-P.10.fid
SI-4
7.26 CDCl3
4.86
4.47
4.18
4.17
3.51
2.45
2.44
2.44
1.80 H2O
1.34
0.97
1.88
2.06
2.00
0.86
6.08
f1 (ppm)

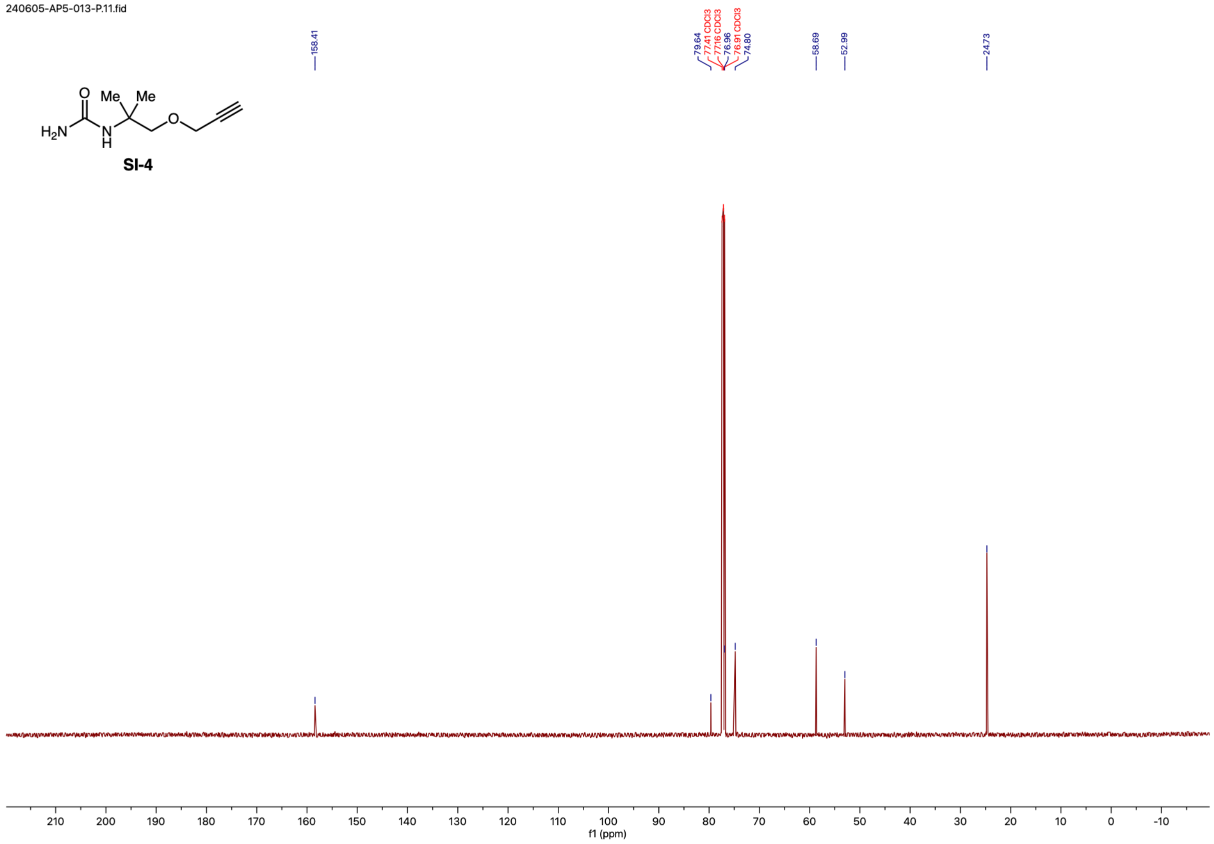
240605-AP5-013-P.11.fid
158.41
79.64
77.41 CDCl3
77.16 CDCl3
76.96
76.91 CDCl3
74.80
58.69
52.99
24.73
SI-4
f1 (ppm)

1316

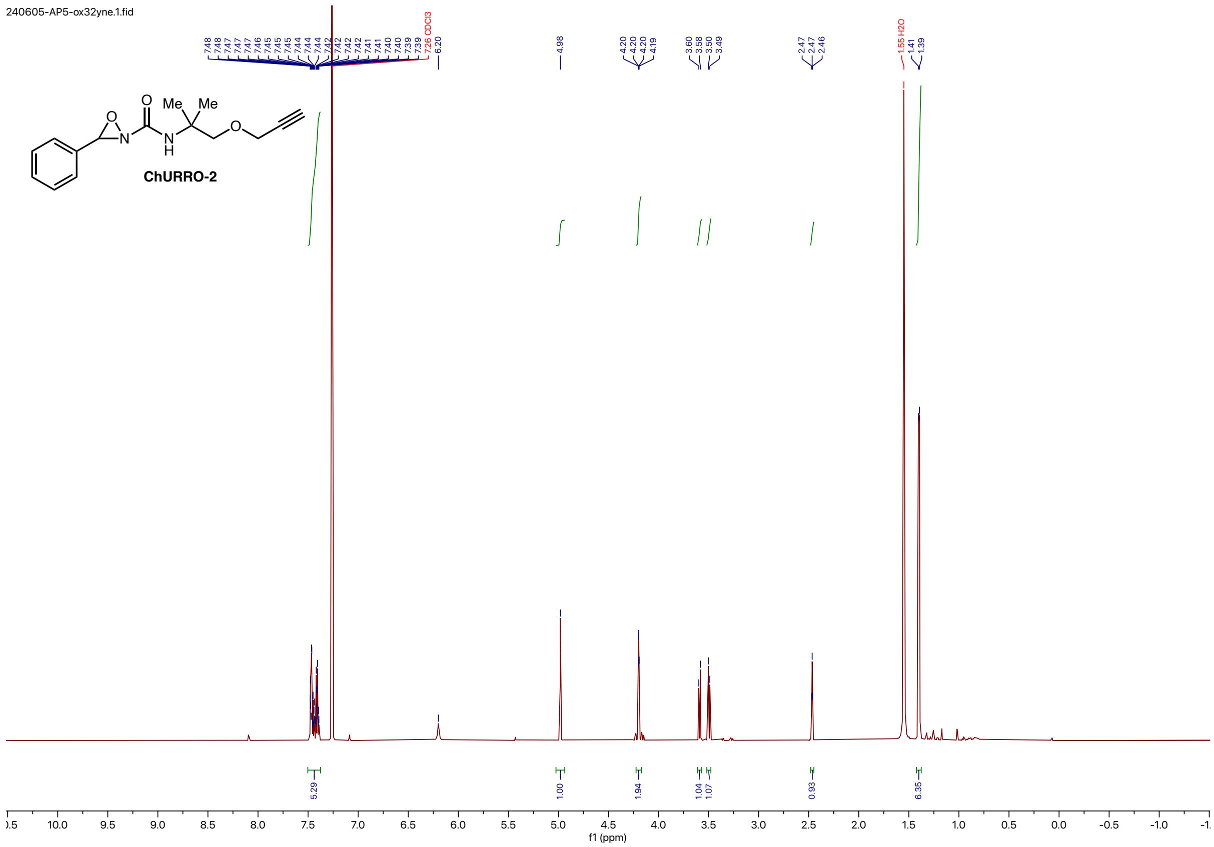

1317

240605-AP5-rac-ox32yne-carbon.1.fid

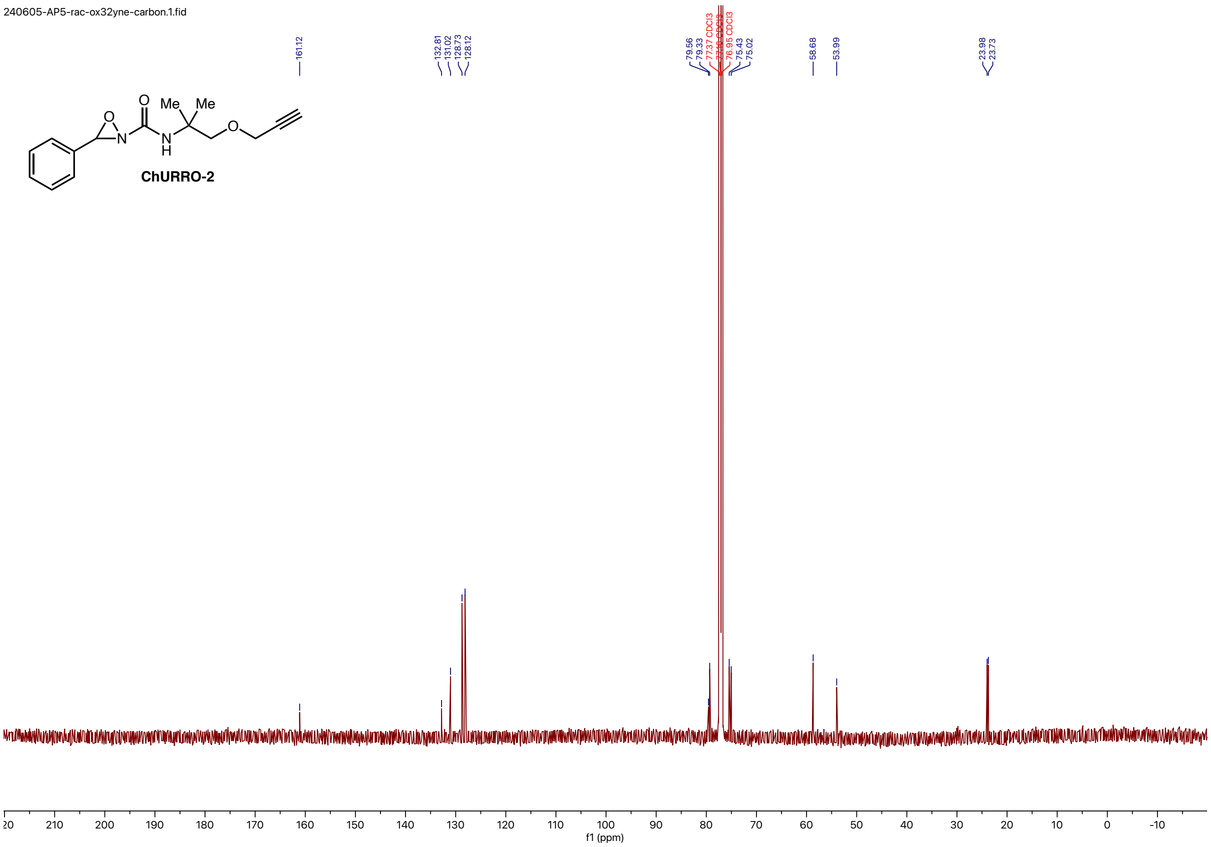

1318

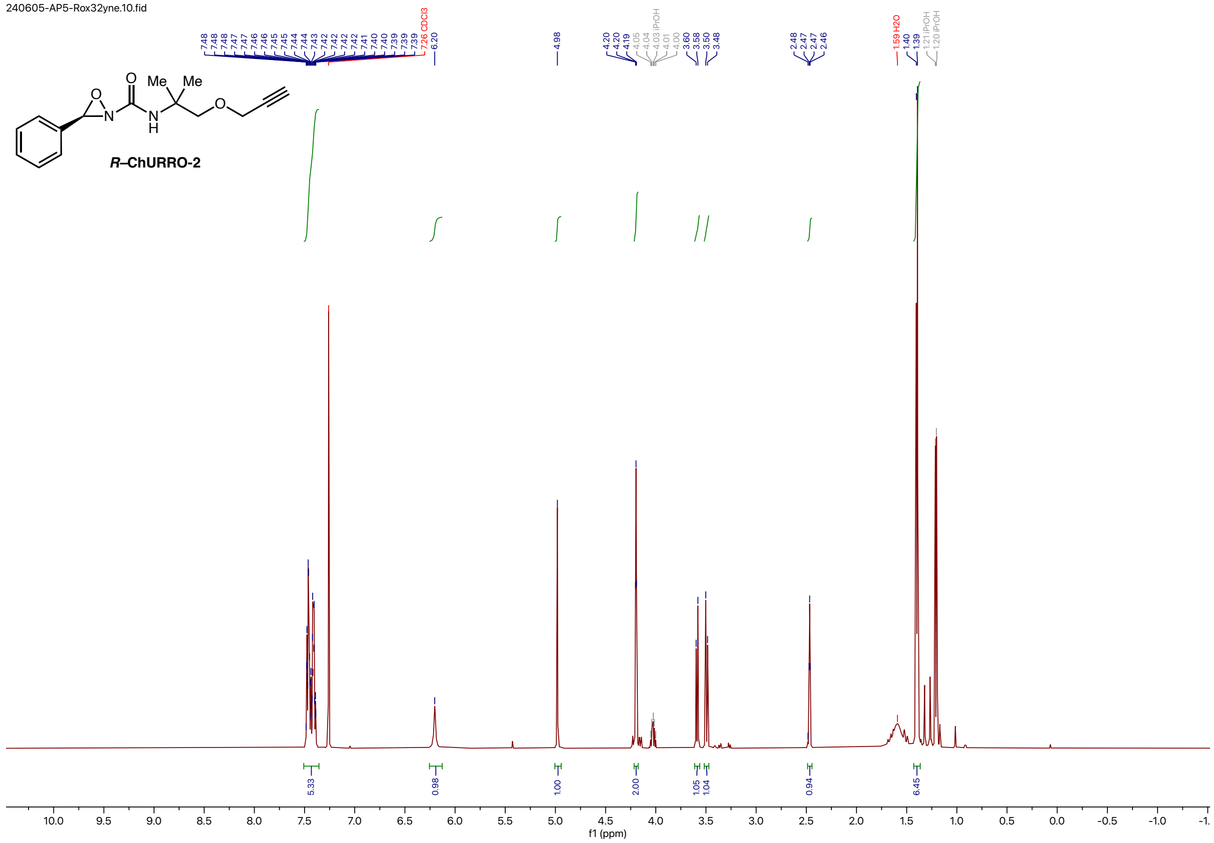

1319

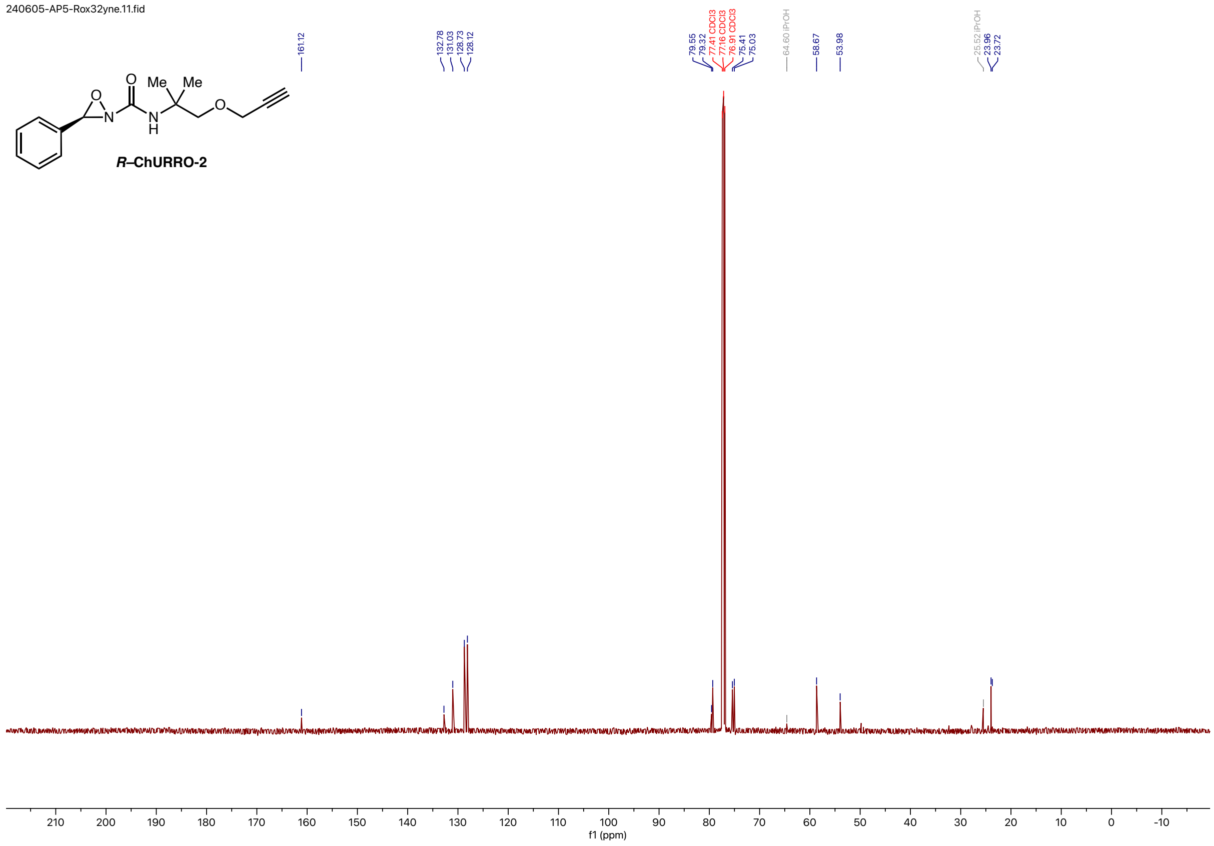
240605-AP5-Rox32yne.11.fid
161.12
132.78
131.03
128.73
128.12
79.55
79.32
77.41 CDCl3
77.16 CDCl3
76.91 CDCl3
75.41
75.03
64.60 iPrOH
58.67
53.98
25.52 iPrOH
23.96
23.72
O
N
O
Me
Me
N
H
O
R–ChURRO-2
210 200 190 180 170 160 150 140 130 120 110 100 90 80 70 60 50 40 30 20 10 0 -10
f1 (ppm)

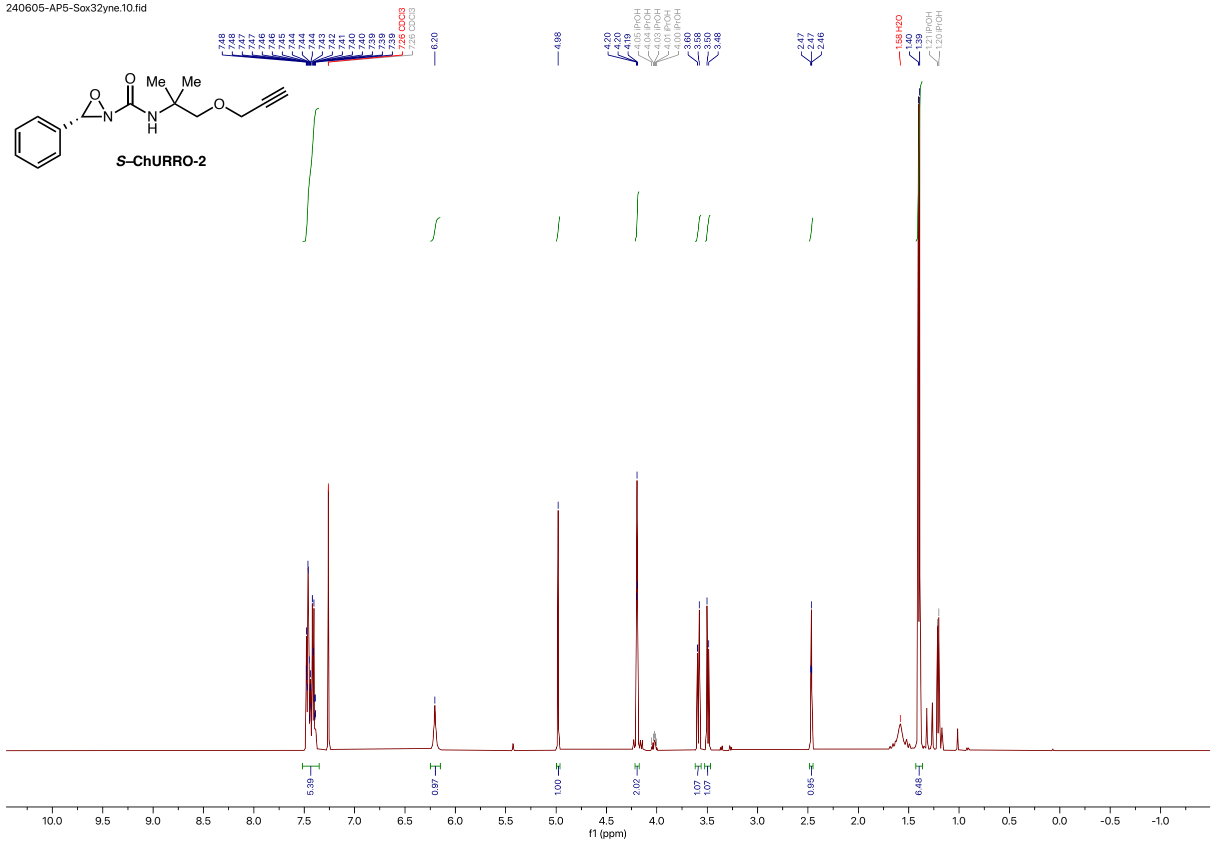
240605-AP5-Sox32yne.10.fid
S–ChURRO-2
f1 (ppm)

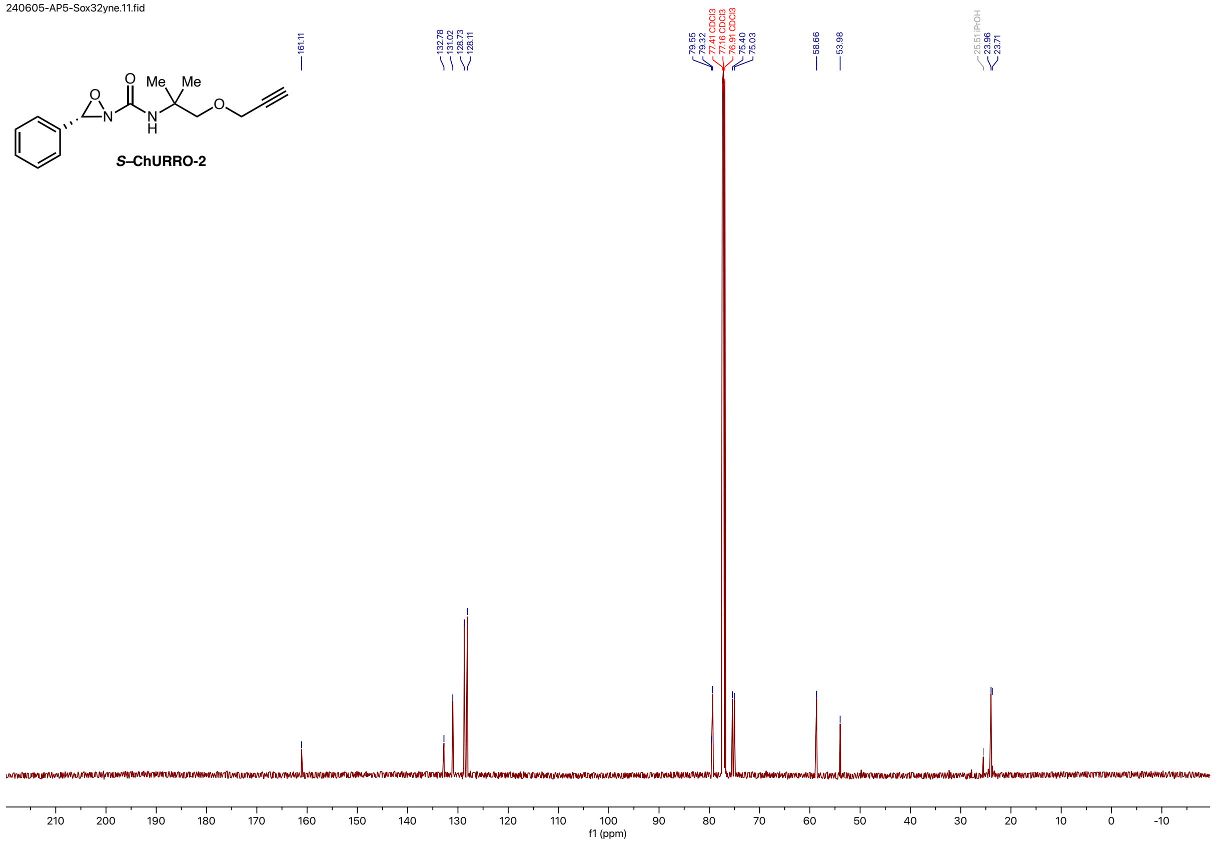

240605-AP5-Sox32yne.11.fid
161.11
132.78
131.02
128.73
128.11
79.55
79.32
77.41 CDCl3
77.16 CDCl3
76.91 CDCl3
75.40
75.03
58.66
53.98
25.51 iPrOH
23.96
23.71
O
N
O
Me
Me
N
H
O
S–ChURRO-2
210
200
190
180
170
160
150
140
130
120
110
100
90
80
70
60
50
40
30
20
10
0
-10
f1 (ppm)

1324
1325

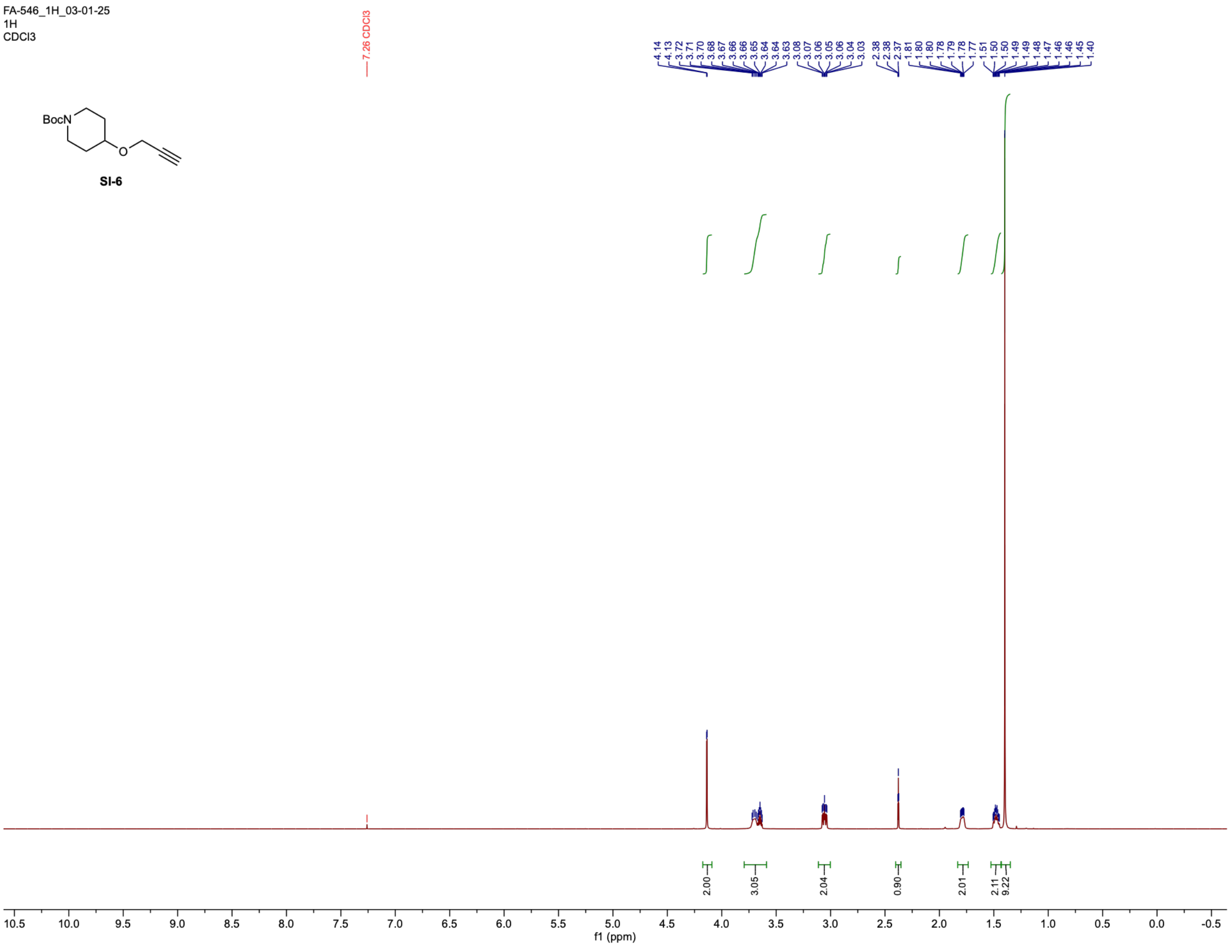
FA-546_1H_03-01-25
1H
CDCl3
BocN
O
SI-6
7.26 CDCl3
4.14
4.13
3.72
3.71
3.70
3.68
3.67
3.66
3.66
3.65
3.64
3.64
3.63
3.08
3.07
3.06
3.05
3.06
3.04
3.03
2.38
2.38
2.37
1.81
1.80
1.80
1.78
1.79
1.78
1.77
1.51
1.50
1.50
1.49
1.49
1.48
1.47
1.46
1.46
1.45
1.40
2.00
3.05
2.04
0.90
2.01
2.11
9.22
10.5
10.0
9.5
9.0
8.5
8.0
7.5
7.0
6.5
6.0
5.5
5.0
4.5
4.0
3.5
3.0
2.5
2.0
1.5
1.0
0.5
0.0
-0.5
f1 (ppm)

1328

FA-546_13C_03-01-25
13C
CDCl3

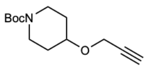

SI-6

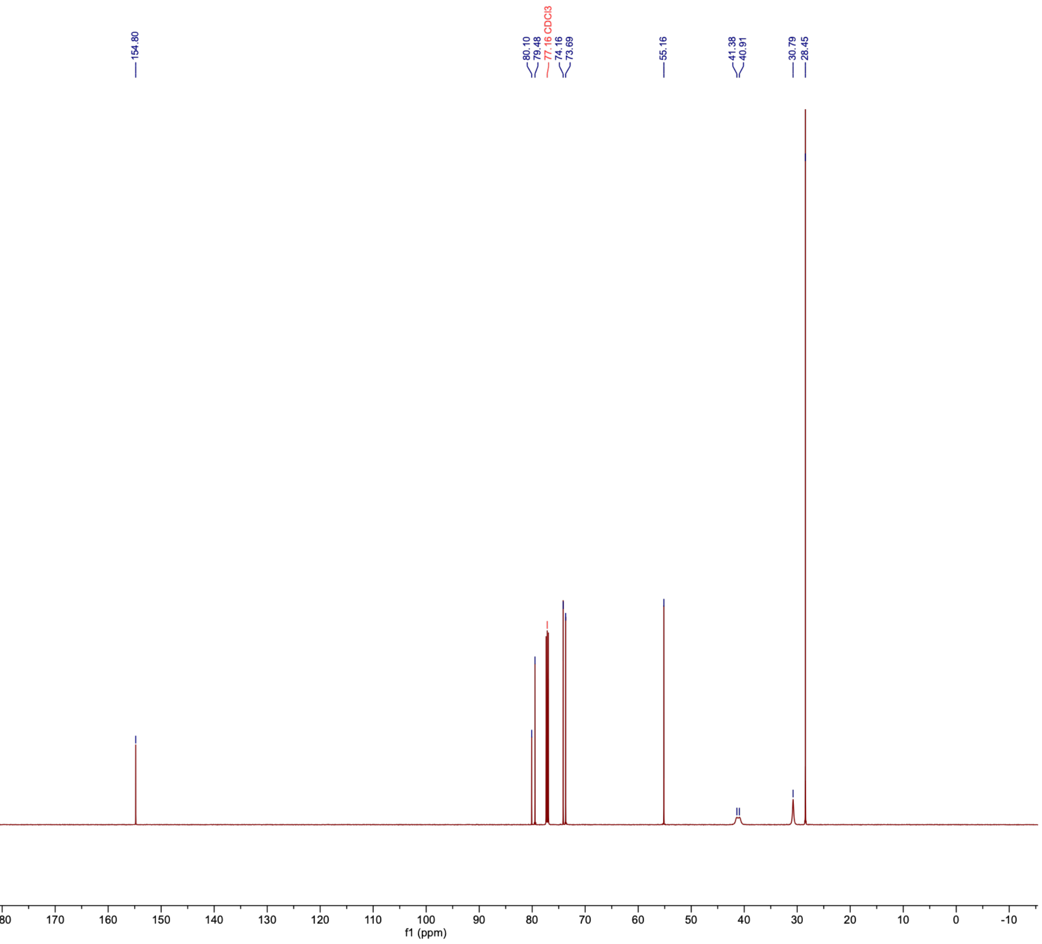

1329

FA-547_E446_03-01-25
1H
MeOD

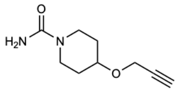

SI-8

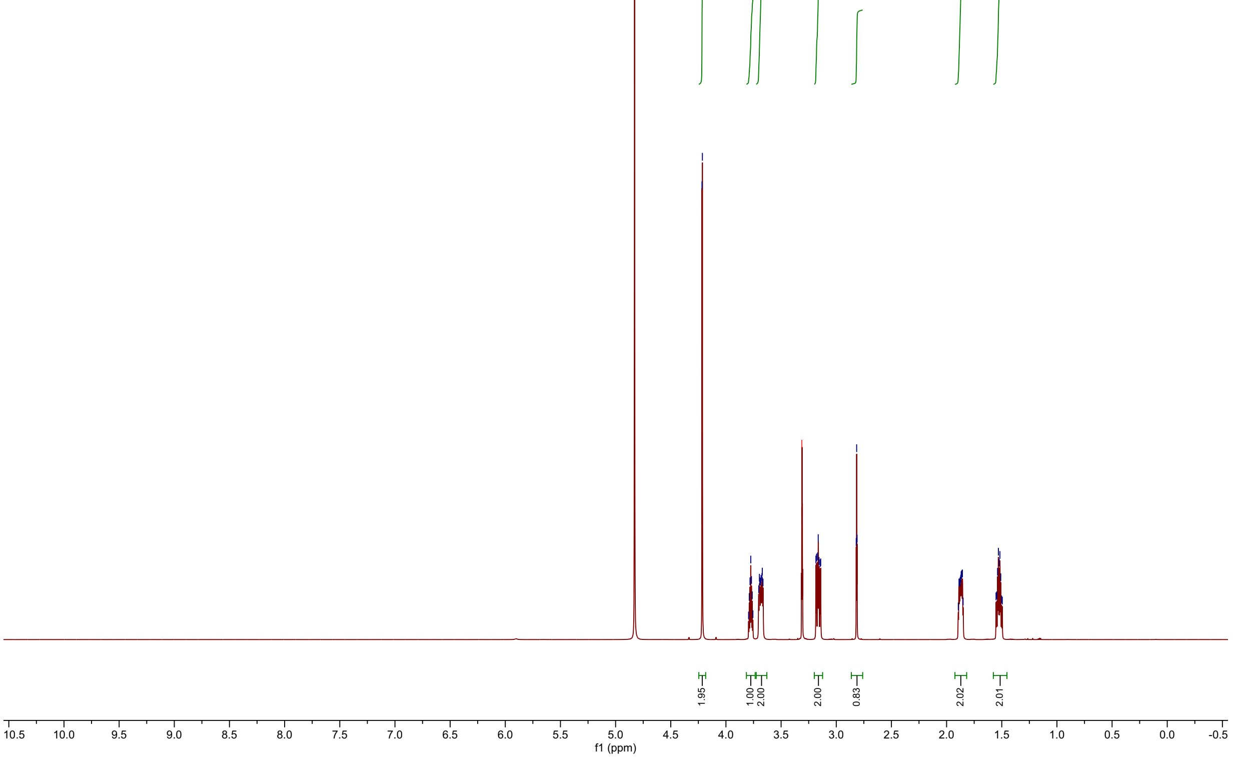

1330

FA-547_E446_13C_03-01-25
13C
MeOD

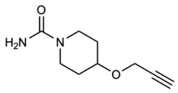

SI-8

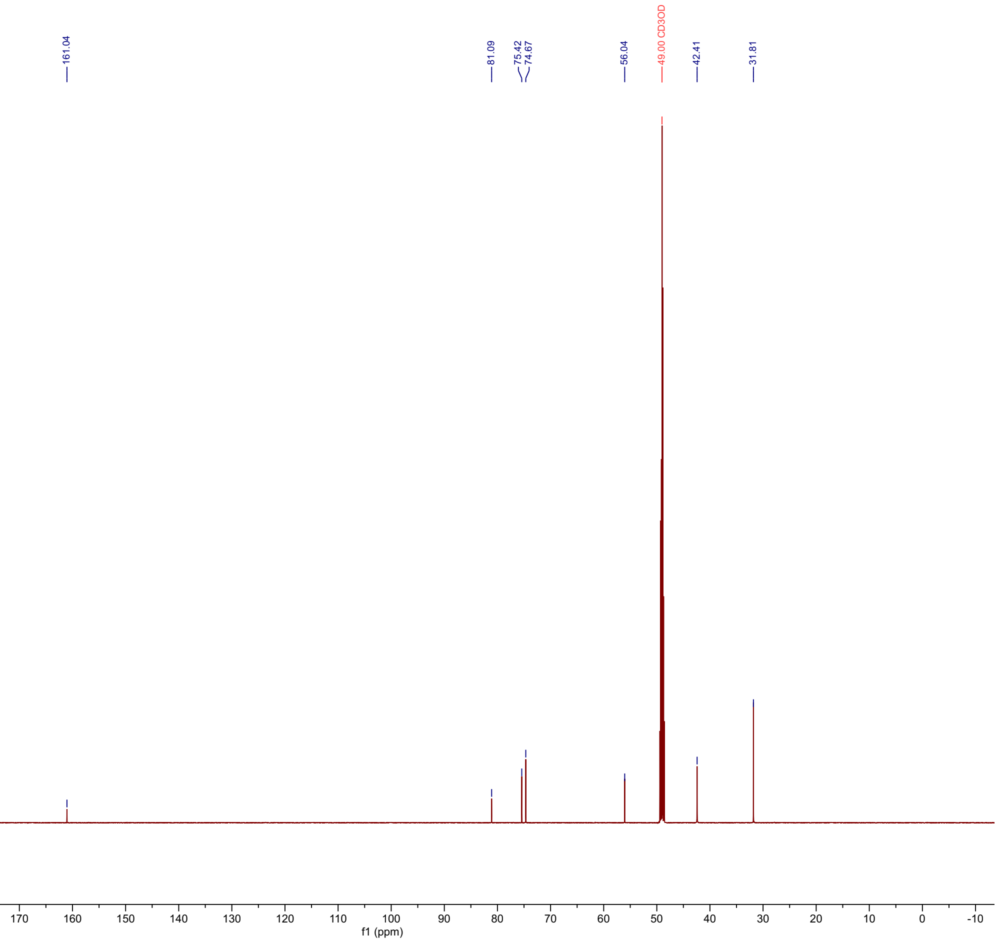

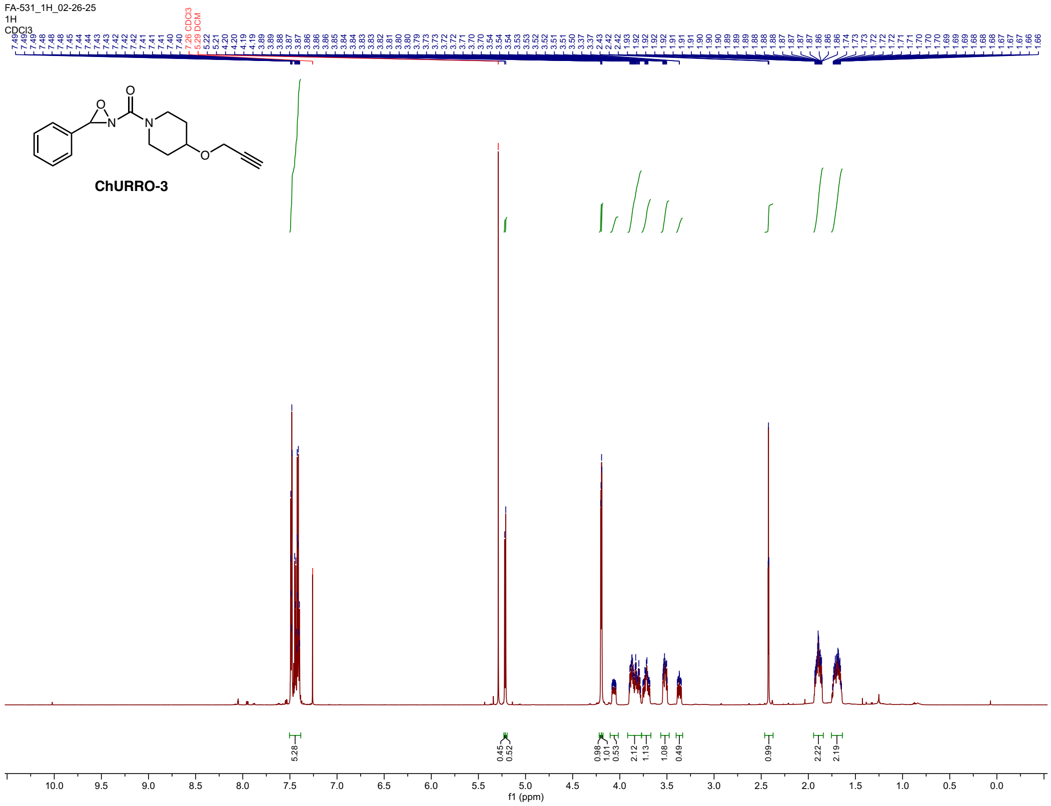

1331

FA-531_13C_02-26-25
13C
CDCl3

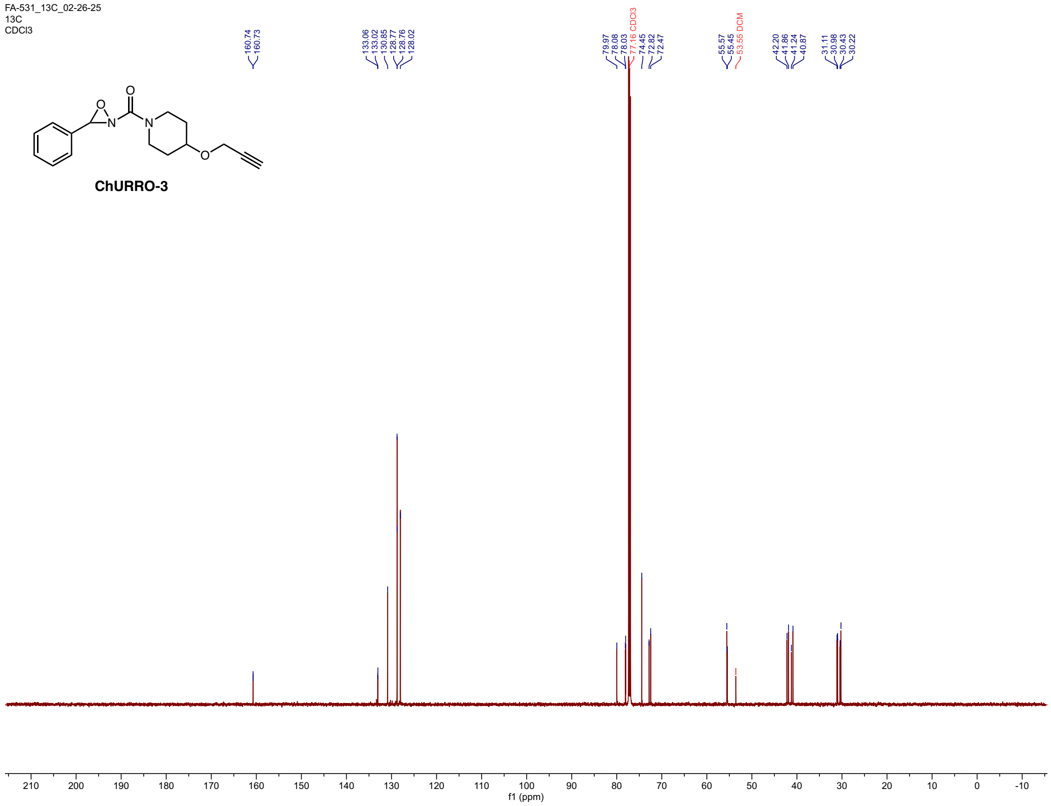

1332

1333

FA-536_1H_02-26-25
1H
CDCl3

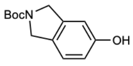

SI-10

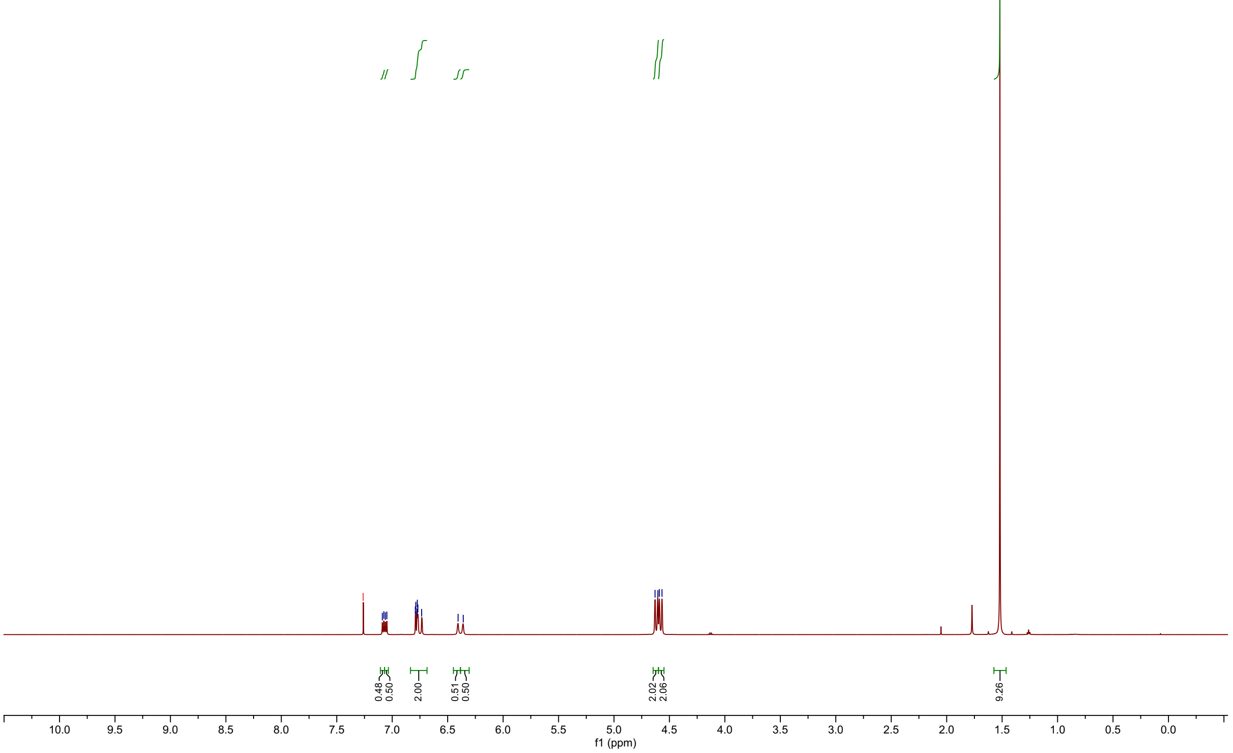

1334

FA-536_13C_02-26-25
13C
CDCl3

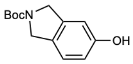

SI-10

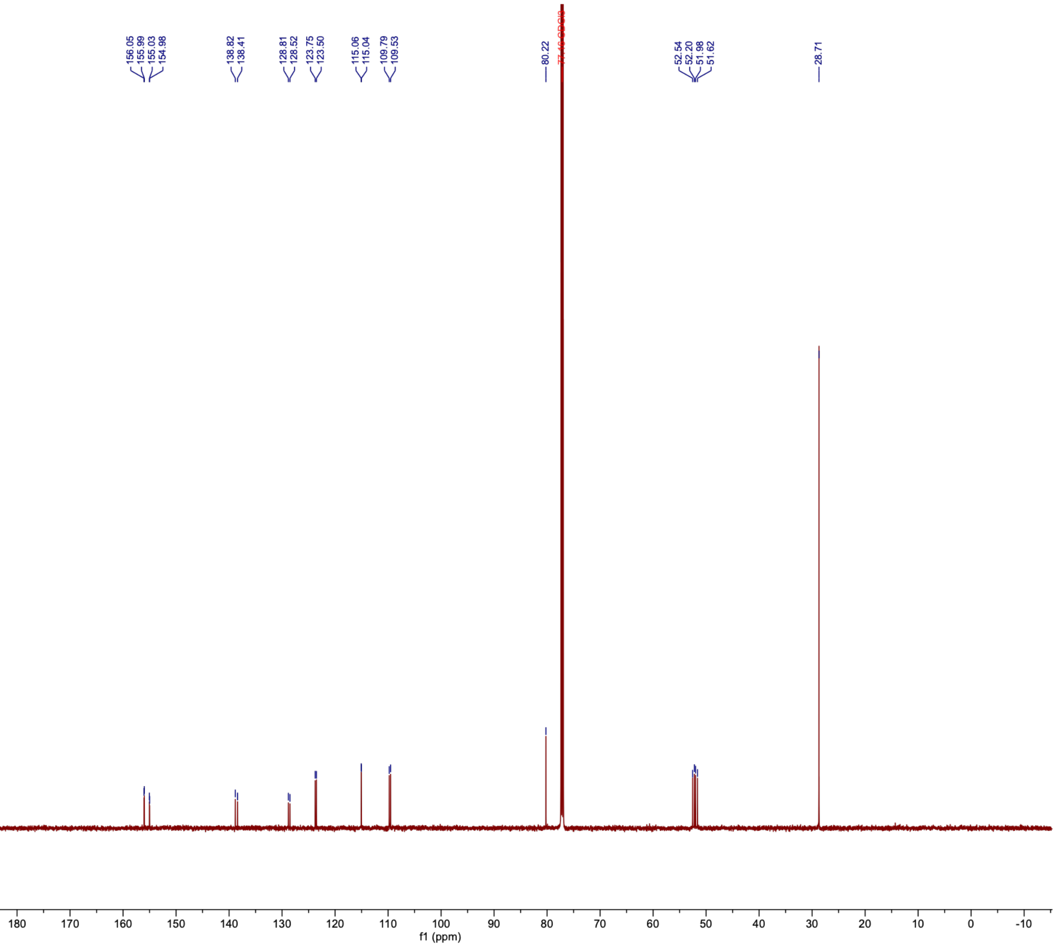

1335

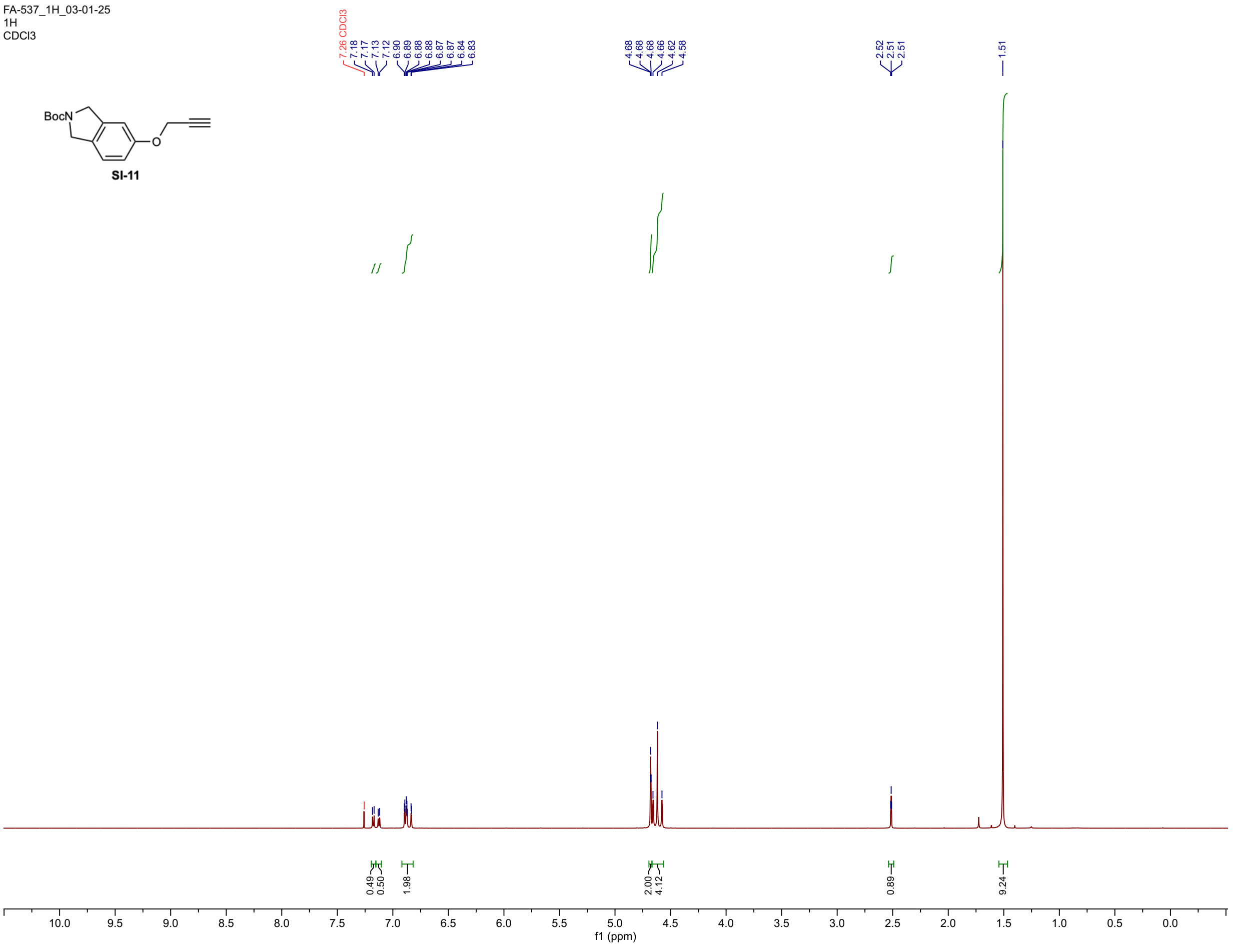
FA-537_1H_03-01-25
1H
CDCl3
BocN
O
SI-11
7.26 CDCl3
7.18
7.17
7.13
7.12
6.90
6.89
6.88
6.88
6.87
6.87
6.84
6.83
4.68
4.68
4.68
4.66
4.62
4.58
2.52
2.51
2.51
1.51
0.49
0.50
1.98
2.00
4.12
0.89
9.24
f1 (ppm)

1336

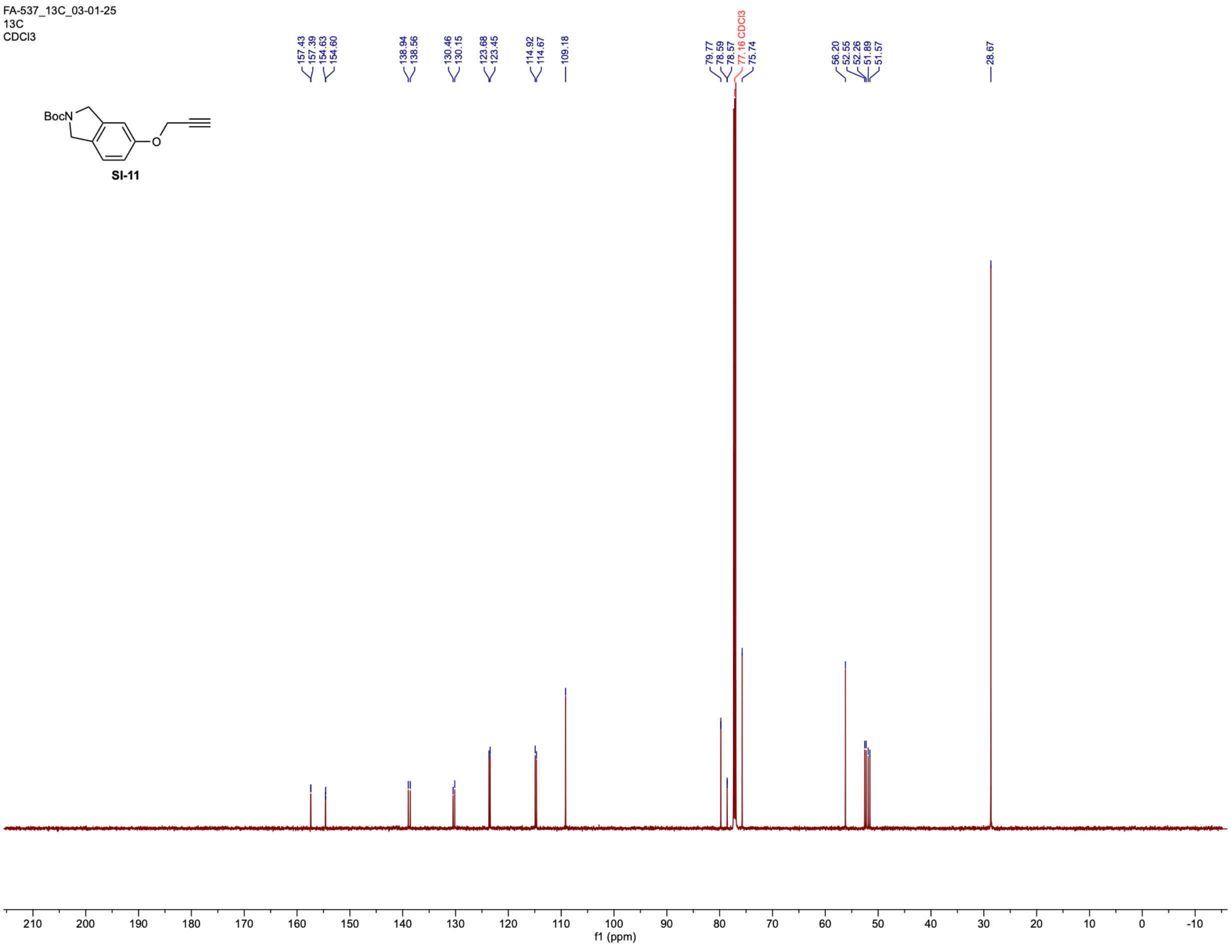

FA-537_13C_03-01-25
13C
CDCl3
BocN
O
SI-11
157.43
157.39
154.63
154.60
138.94
138.56
130.46
130.15
123.68
123.45
114.92
114.67
109.18
79.77
78.59
78.57
77.16 CDCl3
75.74
56.20
52.55
52.26
51.89
51.57
28.67
210
200
190
180
170
160
150
140
130
120
110
100
90
80
70
60
50
40
30
20
10
0
-10
f1 (ppm)

1337

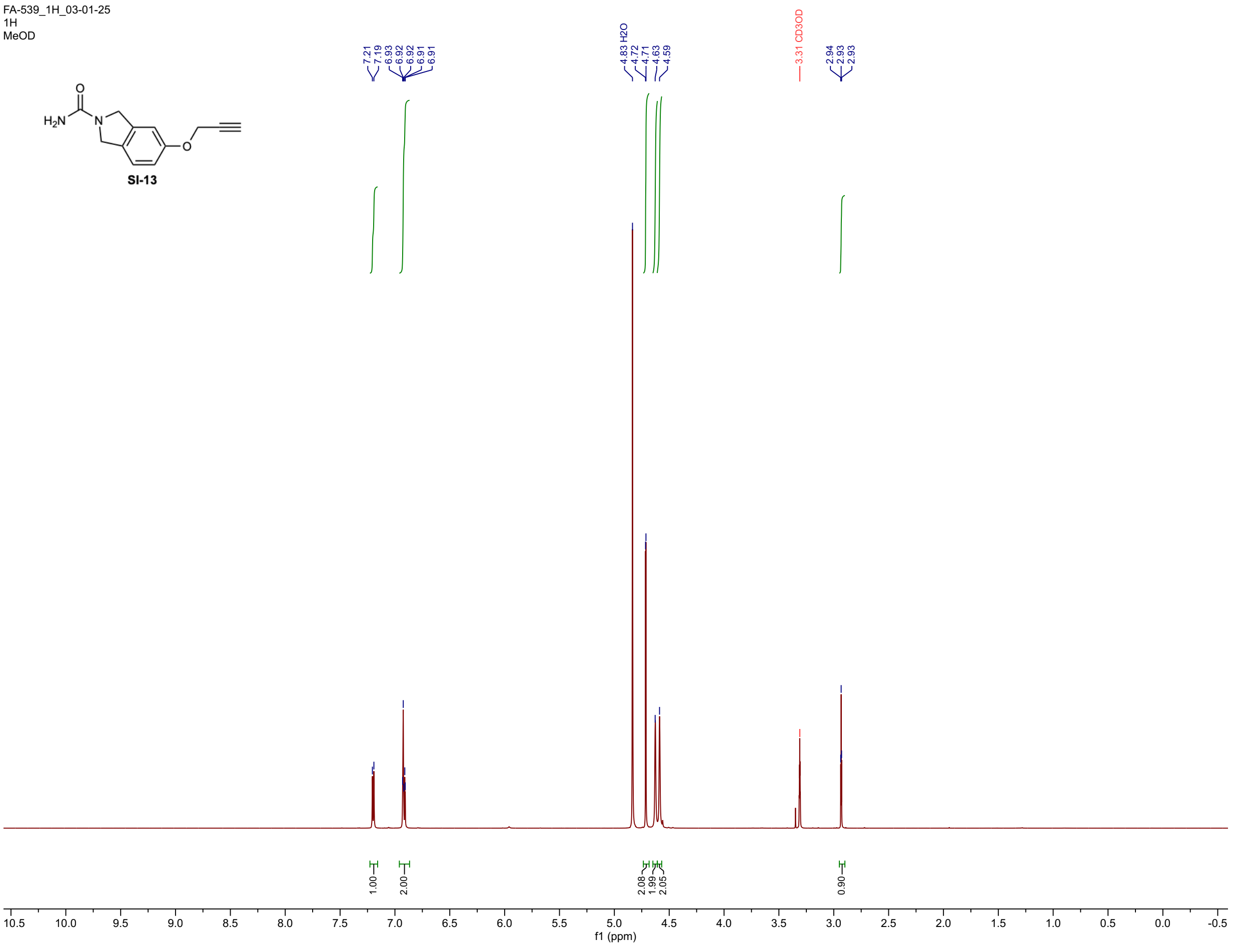
FA-539_1H_03-01-25
1H
MeOD
7.21
7.19
6.93
6.92
6.92
6.91
6.91
4.83 H2O
4.72
4.71
4.63
4.59
3.31 CD3OD
2.94
2.93
2.93
SI-13
1.00
2.00
2.08
1.99
2.05
0.90
f1 (ppm)

1338

FA-539_13C_03-01-25
13C
MeOD

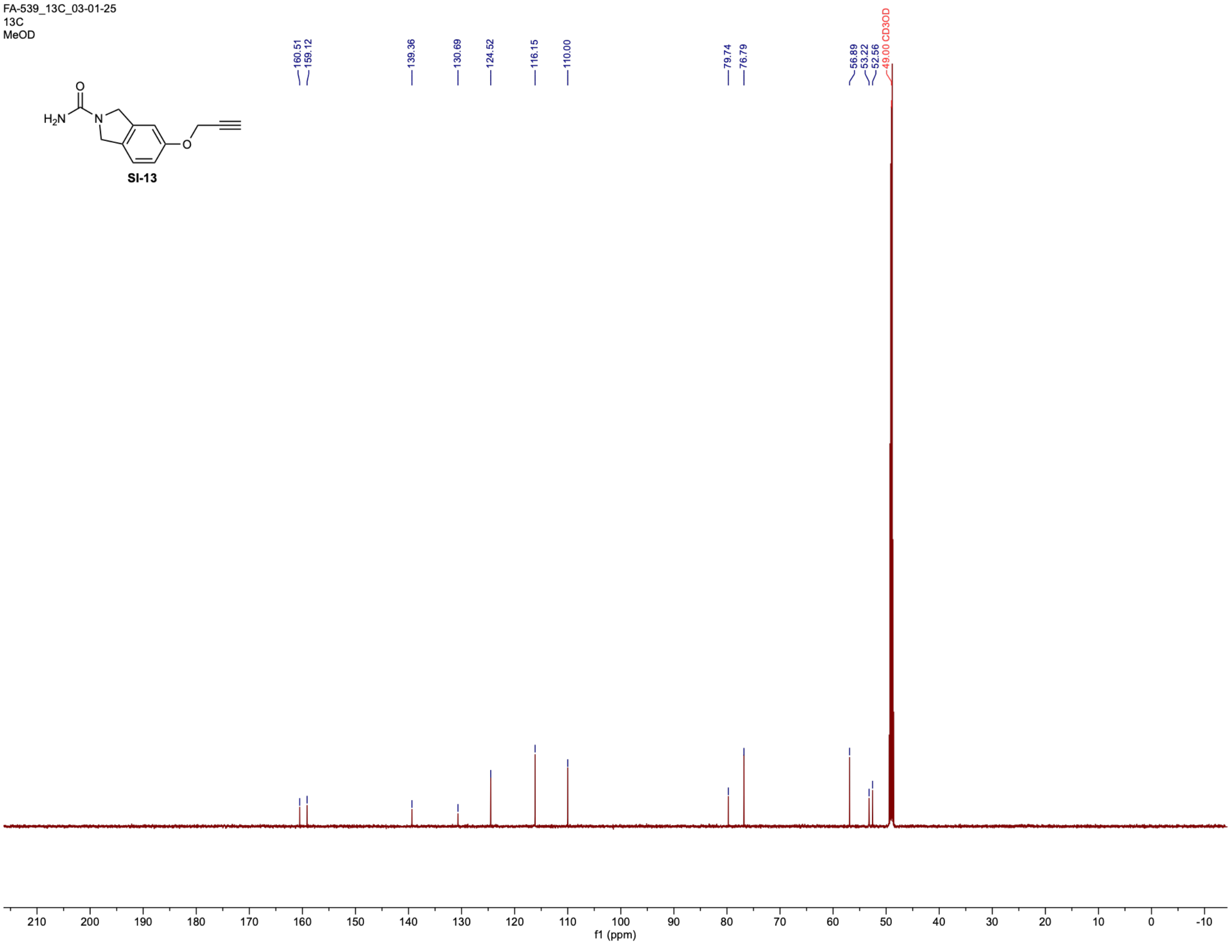

SI-13

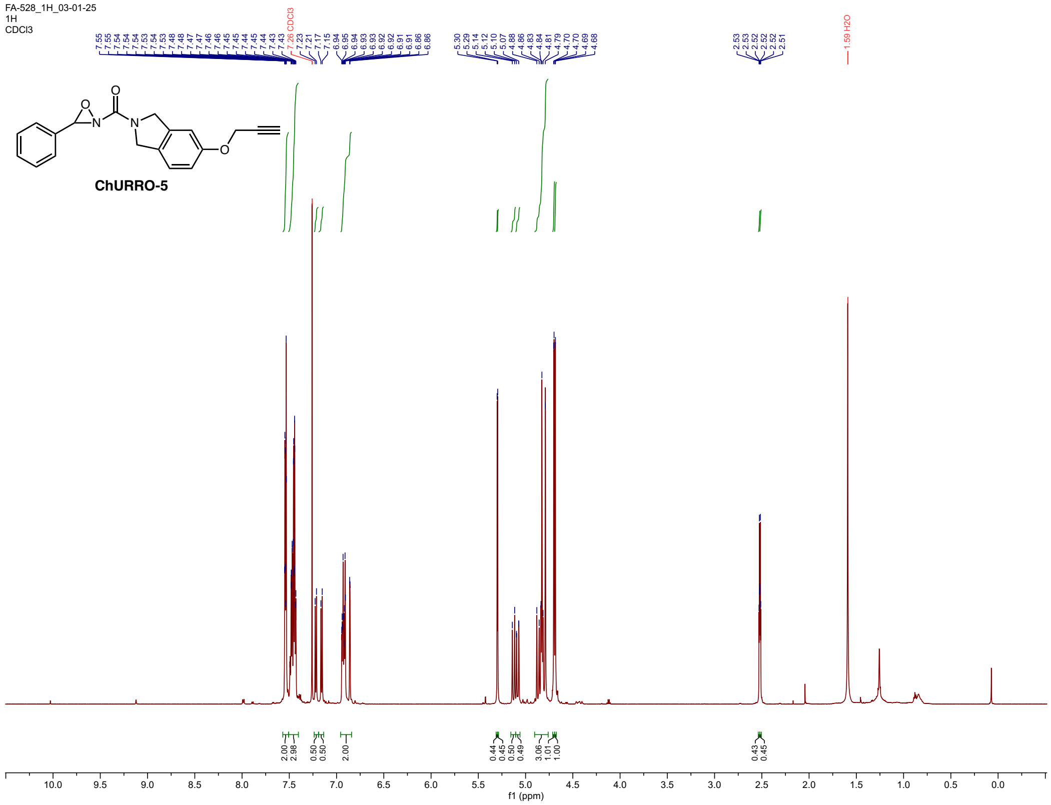

1339

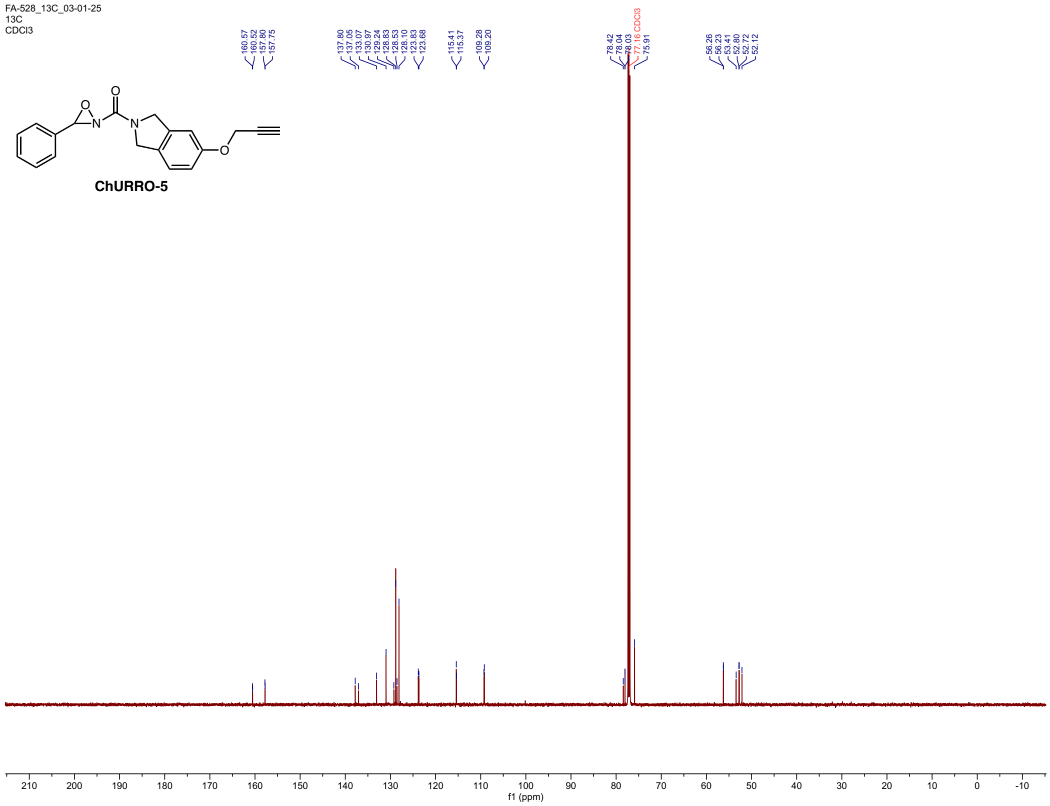
FA-528_13C_03-01-25
13C
CDCl3
160.57
160.52
157.80
157.75
137.80
137.05
133.07
130.97
129.24
128.83
128.53
128.10
123.83
123.68
115.41
115.37
109.28
109.20
78.42
78.04
78.03
77.16 CDCl3
75.91
56.26
56.23
53.41
52.80
52.72
52.12
O
N
ChURRO-5
210 200 190 180 170 160 150 140 130 120 110 100 90 80 70 60 50 40 30 20 10 0 -10
f1 (ppm)

1340

1341

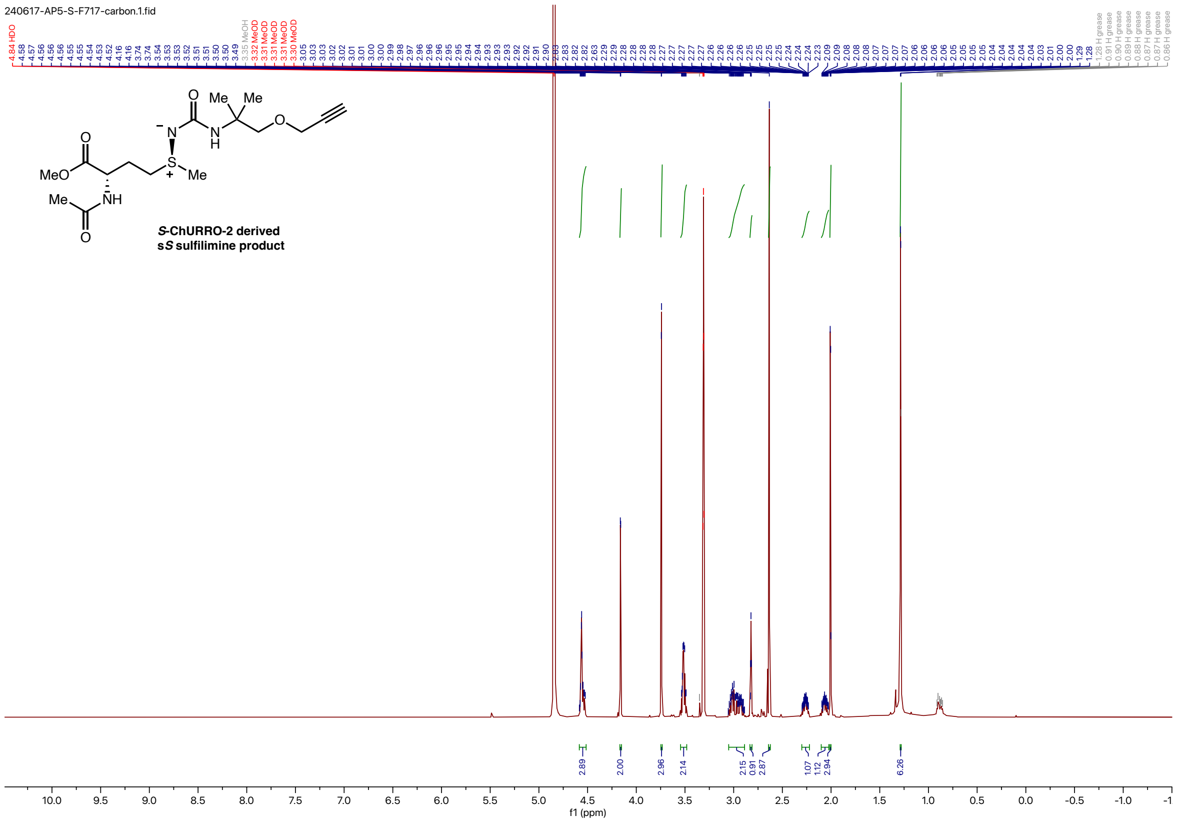

1342
1343

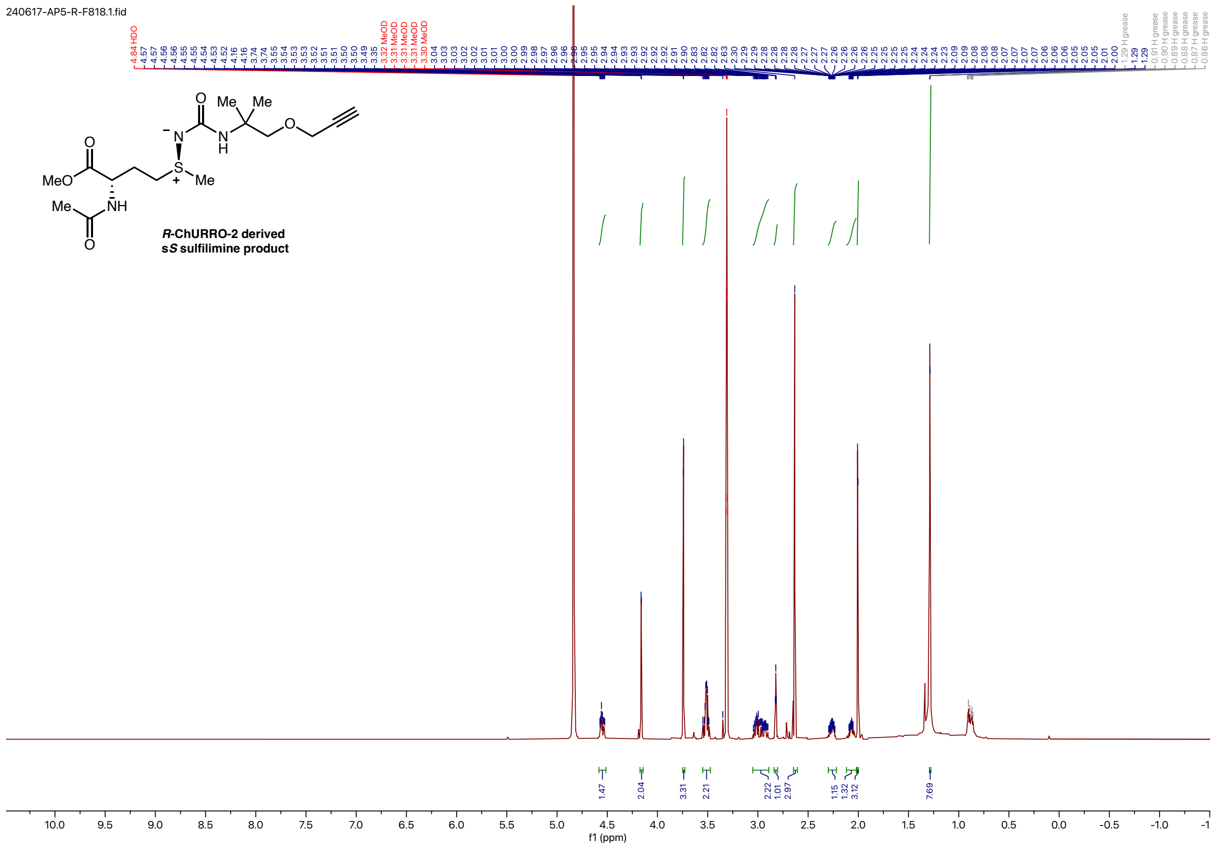
240617-AP5-R-F818.1.fid
R-ChURRO-2 derived
sS sulfilimine product
f1 (ppm)

1344
