# Supplementary material for: Chiral methionine oxidation reagents reveal stereospecific proteome modifications
Source: bioRxiv. 2026 Mar 26:2026.03.24.713977. Preprint. [Version 1] doi: 10.64898/2026.03.24.713977 (PMC13042024; doi:10.64898/2026.03.24.713977)
Supplement: Supplement 2 [file NIHPP2026.03.24.713977v1-supplement-2.pdf]

bioRxiv preprint doi: https://doi.org/10.64898/2026.03.24.713977; this version posted March 26, 2026. The copyright holder for this preprint (which was not certified by peer review) is the author/funder, who has granted bioRxiv a license to display the preprint in perpetuity. It is made available under aCC-BY 4.0 International license.

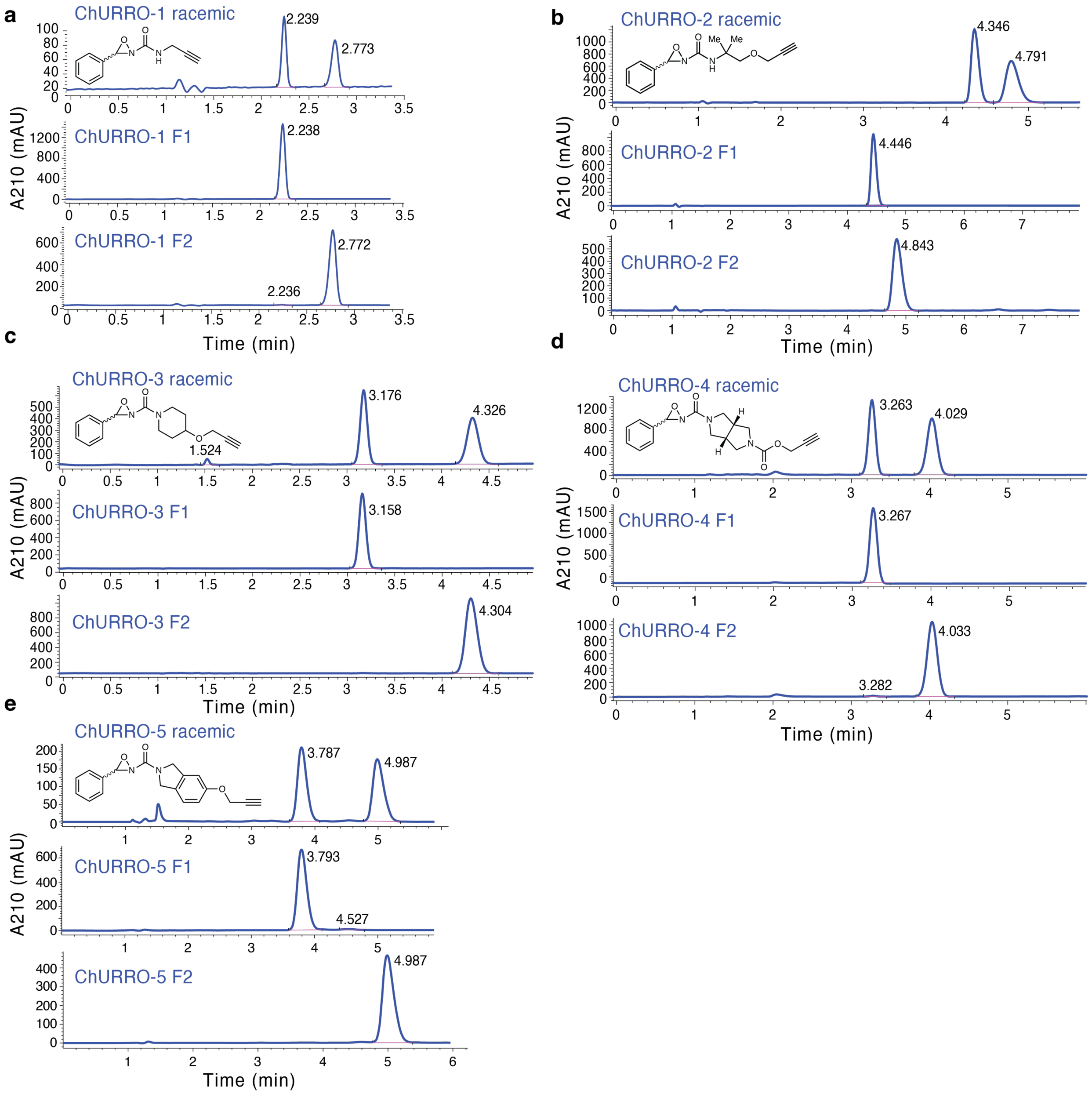

Supplementary Fig. 1: Chiral SFC separation of ChURRO probe enantiomers. a, ChURRO-1 preparative SFC. (*R*)-ChURRO-1 is the first eluting enantiomer and enantiopurity was determined by analytical chiral SFC retention time: 2.2 min (99%). (*S*)-ChURRO-1 is the second eluting enantiomer and enantiopurity was determined by analytical chiral SFC retention time: 2.8 min (99%). **b**, ChURRO-2 preparative SFC. (*R*)-ChURRO-2 is the first eluting enantiomer and enantiopurity was determined by analytical chiral SFC retention time: 4.4 min (99%). (*S*)-ChURRO-2 is the second eluting enantiomer and enantiopurity was determined by analytical chiral SFC retention time: 4.8 min (99%). **c**, ChURRO-3 preparative SFC. (*R*)-ChURRO-3 is the first eluting enantiomer and enantiopurity was determined by analytical chiral SFC retention time: 3.2 min (99%). (*S*)-ChURRO-3 is the second eluting enantiomer and enantiopurity was determined by analytical chiral SFC retention time: 4.3 min (99%). **d**, ChURRO-4 preparative SFC. (*R*)-ChURRO-4 is the first eluting enantiomer and enantiopurity was determined by analytical

bioRxiv preprint doi: https://doi.org/10.64898/2026.03.24.713977; this version posted March 26, 2026. The copyright holder for this preprint (which was not certified by peer review) is the author/funder, who has granted bioRxiv a license to display the preprint in perpetuity. It is made available under aCC-BY 4.0 International license.

chiral SFC retention time: 3.2 min (99%). (*S*)-ChURRO-4 is the second eluting enantiomer and enantiopurity was determined by analytical chiral SFC retention time: 4.3 min (99%). **e**, ChURRO-5 preparative SFC. (*R*)-ChURRO-5 is the first eluting enantiomer and enantiopurity was determined by analytical chiral SFC retention time: 3.8 min (99%). (*S*)-ChURRO-5 is the second eluting enantiomer and enantiopurity was determined by analytical Chiral SFC retention time: 5.0 min (99%).

bioRxiv preprint doi: https://doi.org/10.64898/2026.03.24.713977; this version posted March 26, 2026. The copyright holder for this preprint (which was not certified by peer review) is the author/funder, who has granted bioRxiv a license to display the preprint in perpetuity. It is made available under aCC-BY 4.0 International license.

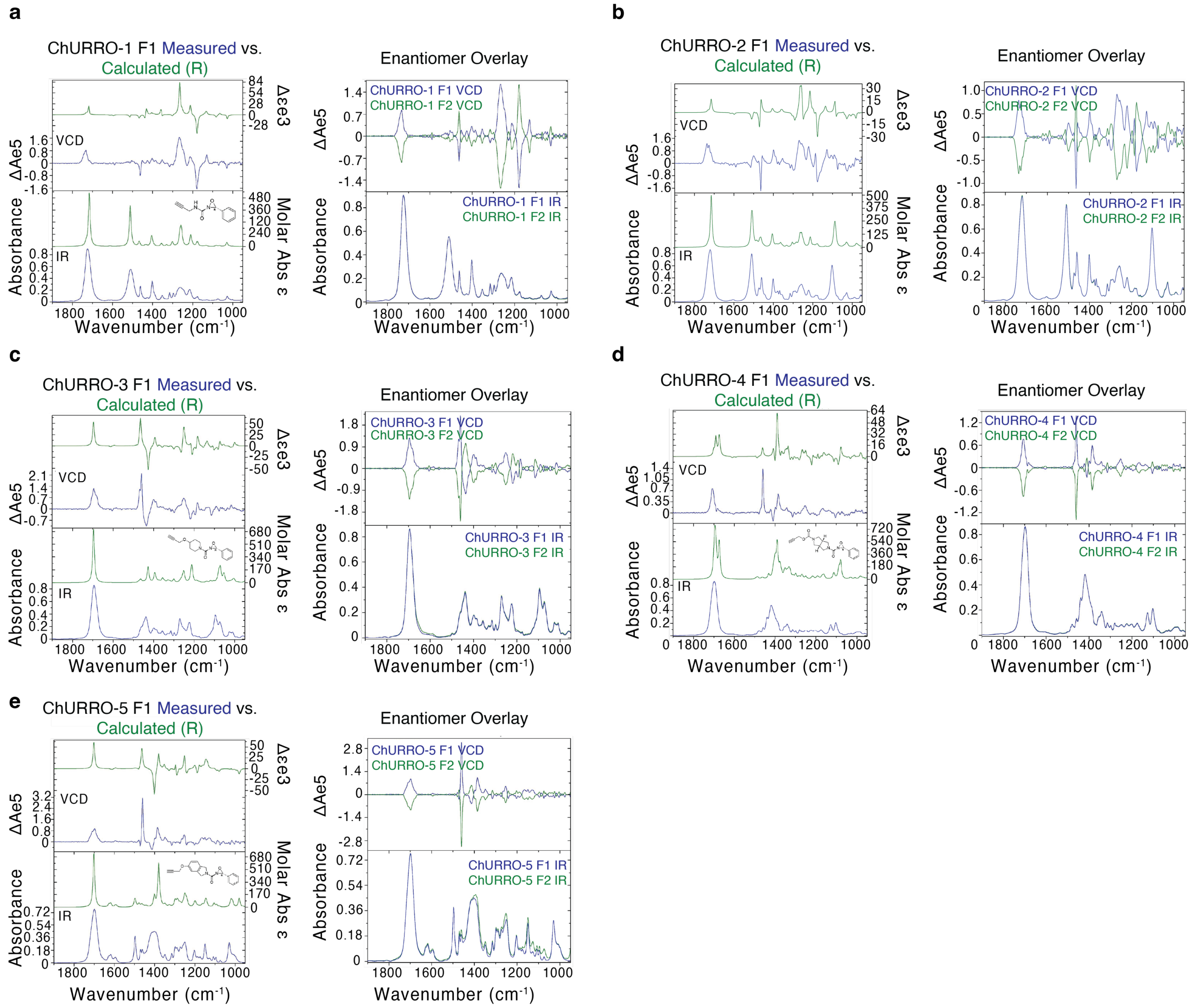

Supplementary Fig. 2: VCD and IR analysis of ChURRO enantiomers. a-e, (Left) measured and calculated IR and VCD spectra of first eluting enantiomer of ChURRO probe, assigned as (*R*)-ChURRO. (Right) overlay of measured VCD and IR spectra of both ChURRO enantiomers assigned as (*R*) and (*S*), respectively, in order of elution on the analytical SFC column.

bioRxiv preprint doi: https://doi.org/10.64898/2026.03.24.713977; this version posted March 26, 2026. The copyright holder for this preprint (which was not certified by peer review) is the author/funder, who has granted bioRxiv a license to display the preprint in perpetuity. It is made available under aCC-BY 4.0 International license.

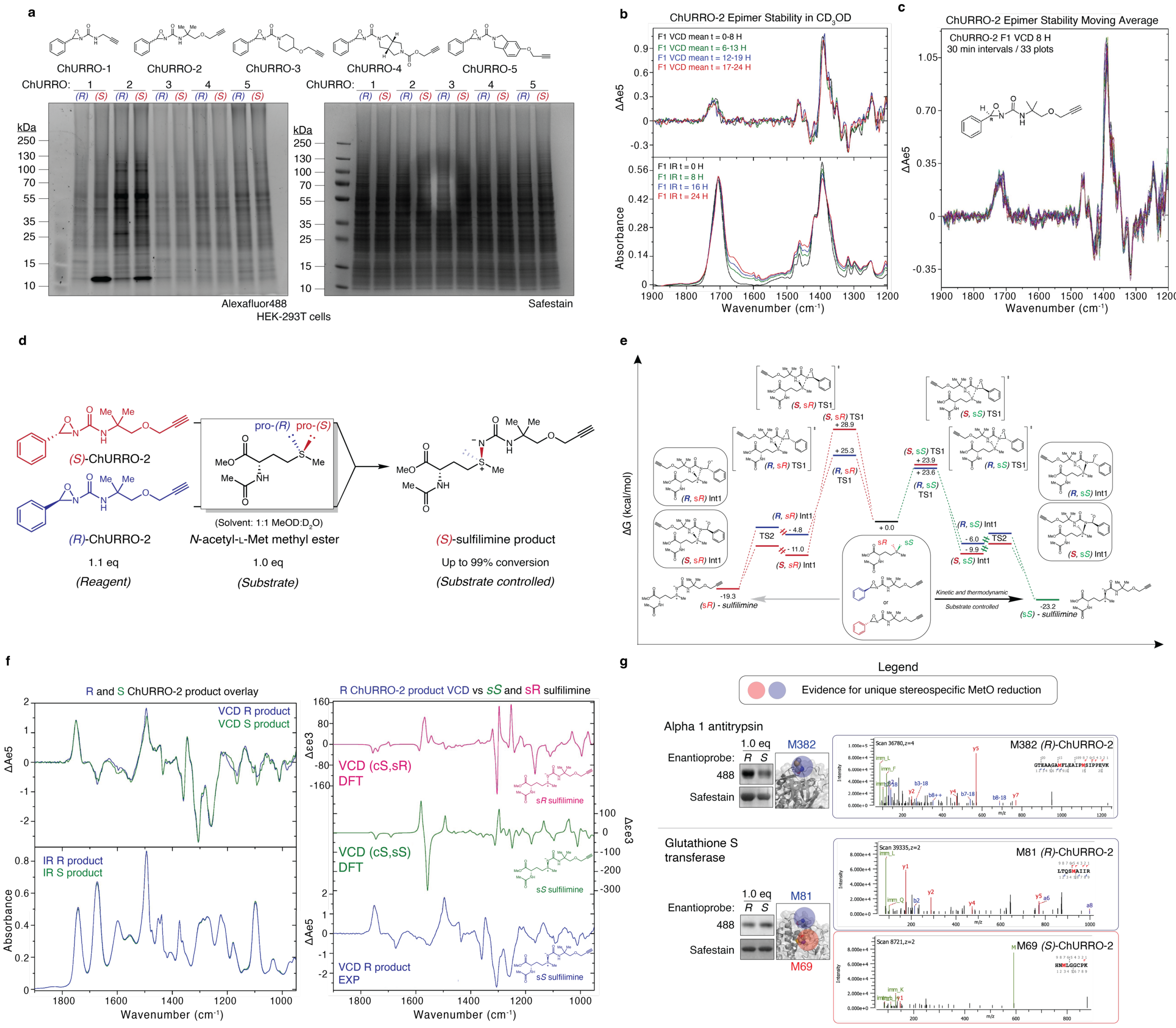

Supplementary Fig. 3: Assessing ChURRO reactivity on monomers, proteins, and proteomes. a, In-gel fluorescence labeling comparison of all 5 ChURRO probes with equimolar doses (100 μM) in HEK-293T lysates (4 mg/mL). **b**, (Top) 8 hour averaged VCD spectra of ChURRO-2 F1 in CD_3OD taken over 24 hours at 37 °C. (Bottom) 8 hour averaged IR spectra of ChURRO-2 F1 in CD_3OD taken over 24 hours. **c**, Half-difference baseline corrected VCD spectra representative of ChURRO-2 F1 and F2 (referred to in text as (*R*) and (*S*)) for the 24 hour time course. **d**, Model diastereoselectivity studies of (*R*)- or (*S*)-ChURRO-2 (50 mM) incubated with *N*-acetyl-L-methionine methyl ester (55 mM) in cosolvent (1:1 CD_3OD:D_2O) at room temperature for 30 minutes. Product was confirmed by ^{1}H NMR and VCD measurement. **e**, Reaction coordinate diagram of (*R*)- or (*S*)-ChURRO-2 reacting with a model methionine substrate. **f**, (Left) overlay of measured VCD and IR spectra of products isolated from reaction of (*R*)-ChURRO-2 and (*S*)-ChURRO-2 with *N*-acetyl methionine methyl ester. (Right) measured VCD spectrum of isolated sulfilimine product from (*R*)-ChURRO-2 reaction vs calculated VCD spectra of (s*R*) and (s*S*) sulfilimine diastereomers. **g**, ChURRO labeling of previously characterized proteins that have evidence for stereospecific MetO reduction assessed by in-gel fluorescence and shotgun proteomics.

bioRxiv preprint doi: https://doi.org/10.64898/2026.03.24.713977; this version posted March 26, 2026. The copyright holder for this preprint (which was not certified by peer review) is the author/funder, who has granted bioRxiv a license to display the preprint in perpetuity. It is made available under aCC-BY 4.0 International license.

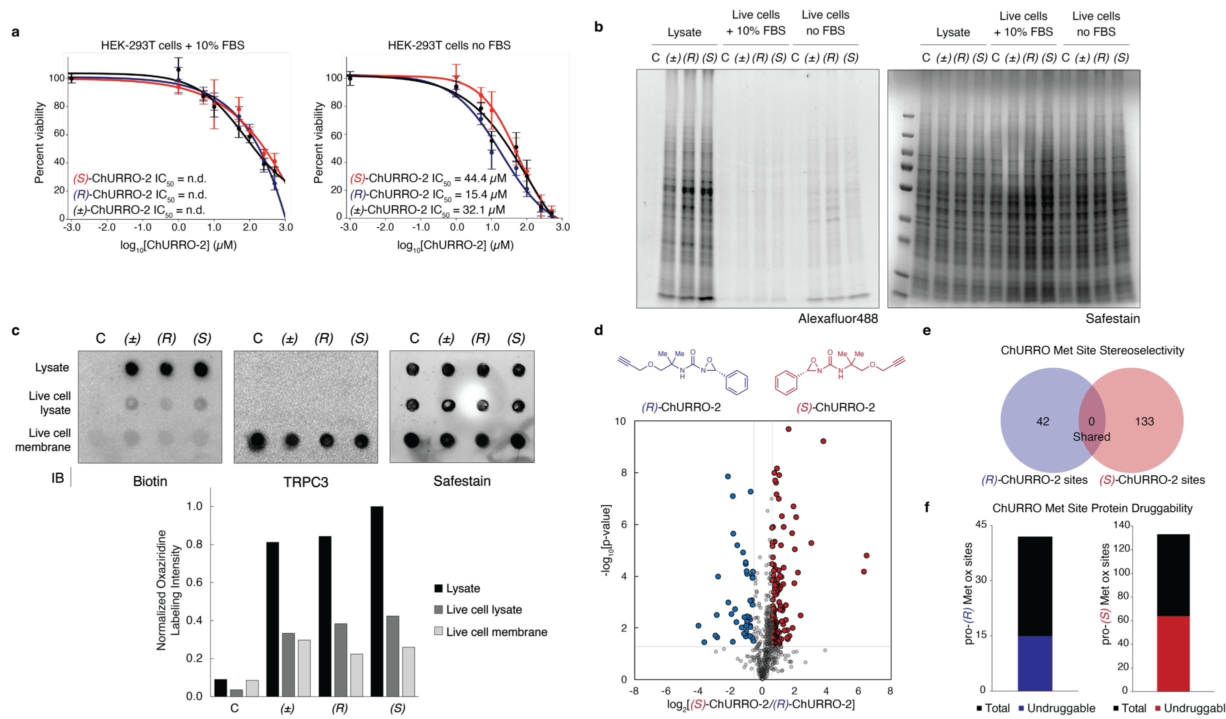

Supplementary Fig. 4: Live cell and lysate labeling experiments of ChURRO-2. a, Dose response curves of cell viability with or without FBS in the media (n = 3 bioreplicates, error bars represent standard deviation). **b**, In-gel fluorescence labeling comparison of equimolar doses of ChURRO-2 enantiomers (50 μM). **c**, Dot blot assessment to determine probe localization to the plasma membrane by TRPC3 staining (membrane marker). **d**, Volcano plots of quantitative proteomic experiments conducted in HEK-293T lysates with equimolar 200 μM ChURRO-2 enantiomer doses in 4 mg/mL lysate (n = 3 biological replicates, n = 3 technical MS replicates). **e**, proteomic identification of ChURRO-2 methionine sites showcases high stereoselectivity for (*R*)- and (*S*)-Met oxidation sites. **f**, ChURRO-2 identifies therapeutically relevant Met oxidation sites with high stereoselectivity in HEK-293T lysates.

bioRxiv preprint doi: https://doi.org/10.64898/2026.03.24.713977; this version posted March 26, 2026. The copyright holder for this preprint (which was not certified by peer review) is the author/funder, who has granted bioRxiv a license to display the preprint in perpetuity. It is made available under aCC-BY 4.0 International license.

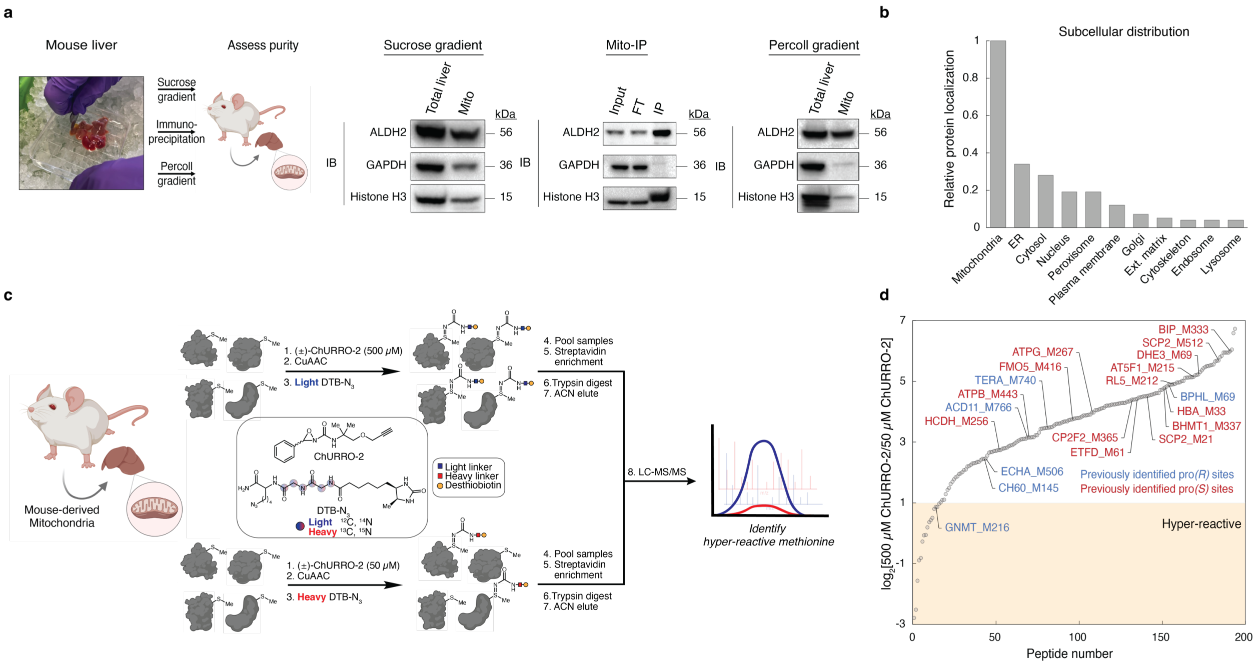

Supplementary Fig. 5: Subcellular ChURRO profiling of the mitochondria. **a**, Comparison of mitochondrial enrichment methods assessed by immunoblotting of ALDH2 (mitochondrial), GAPDH (cytosolic), or Histone H3 (nuclear). Sucrose gradient protocol adapted from Dias et. al[69] and Mito-IP protocol adapted from Chen et. al.[49] **b**, Normalized subcellular distribution of proteins identified from mitochondrial prochiral Met oxidation site dataset. **c**, isoTOP-ABPP workflow to identify hyper-reactive methionine sites in the mitochondria. **d**, Reactivity profiles of Met sites in mitochondria with identified prochiral Met oxidation sites labeled (n = 3 technical MS replicates).

bioRxiv preprint doi: https://doi.org/10.64898/2026.03.24.713977; this version posted March 26, 2026. The copyright holder for this preprint (which was not certified by peer review) is the author/funder, who has granted bioRxiv a license to display the preprint in perpetuity. It is made available under aCC-BY 4.0 International license.

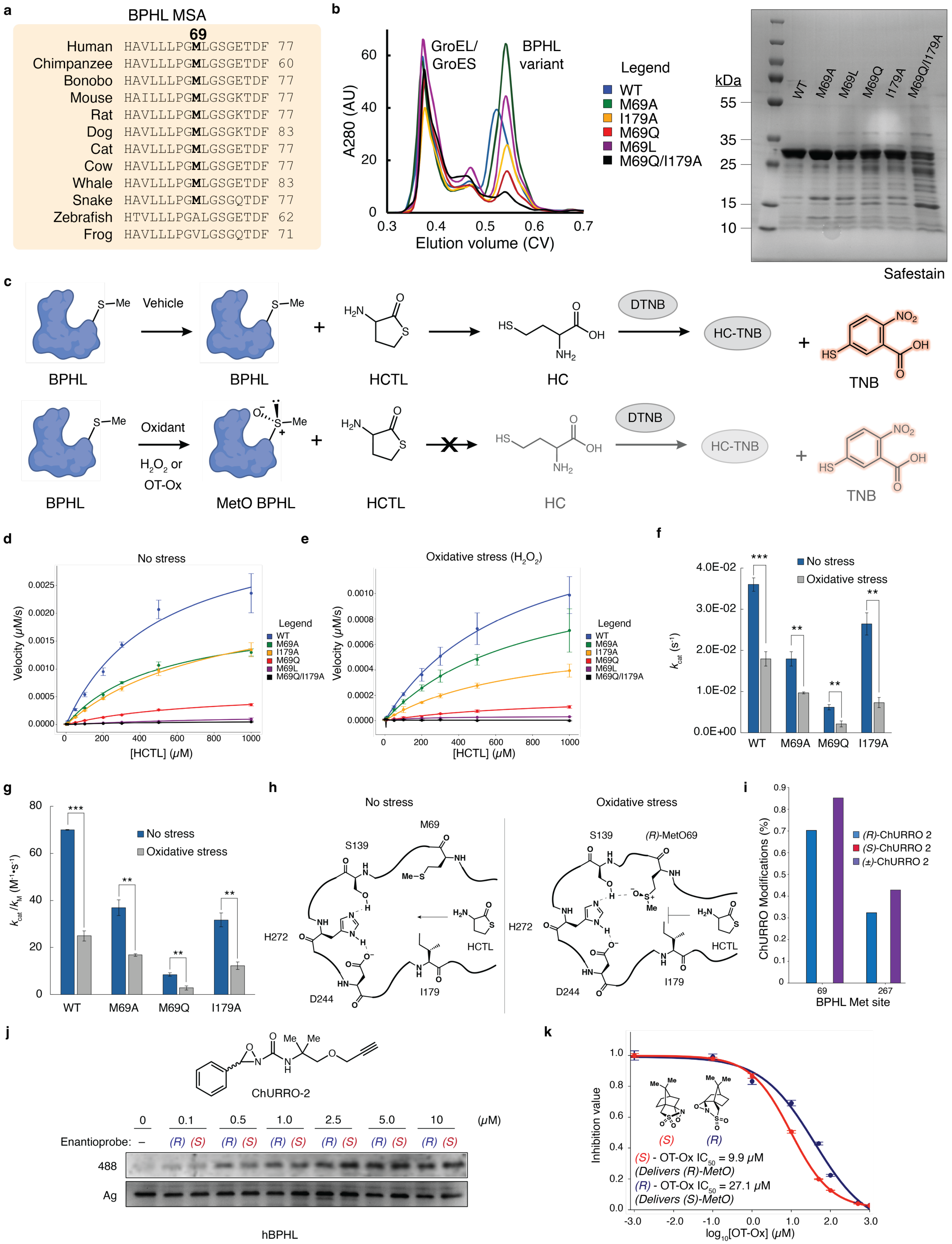

Supplementary Fig. 6: BPHL contains a regulatory M69 site that is stereospecifically oxidized and reduced. a, Clustal omega multiple sequence alignment of BPHL across multiple organisms. **b**, (Left) SEC chromatograms of all BPHL variants generated with aid of protein chaperones GroEL/ES. (Right) SDS-PAGE gel

bioRxiv preprint doi: https://doi.org/10.64898/2026.03.24.713977; this version posted March 26, 2026. The copyright holder for this preprint (which was not certified by peer review) is the author/funder, who has granted bioRxiv a license to display the preprint in perpetuity. It is made available under aCC-BY 4.0 International license.

analysis of purified BPHL variants. **c**, Schematic of *in vitro* BPHL activity assay using native HCTL substrate and Ellman's reagent (DTNB) to oxidize HC and form TNB as a colorimetric readout. **d-e**, HCTLase activity curves of all 6 BPHL variants under conditions of no stress (0 μM H_2O_2) and oxidative stress (500 μM H_2O_2) with determined Michaelis-Menten parameters (n = 3 technical replicates, error bars represent standard deviation). **f-g**, Additional Michaelis-Menten parameters of 4 BPHL variants under conditions of no stress and oxidative stress (n = 3 technical replicates, error bars represent standard deviation). **h**, Proposed mechanism of pro-*(R)*-MetO inhibition at 69 site. **i**, ChURRO-2 shotgun proteomic peptide spectrum matches for different BPHL methionine sites. **j**, Gel-ABPP of ChURRO-2 enantiomers on purified human BPHL. **k**, Dose-response curves of BPHL treated with camphor-derived Davis oxaziridines for asymmetric MetO formation (n = 3 technical replicates, error bars represent standard deviation).

bioRxiv preprint doi: https://doi.org/10.64898/2026.03.24.713977; this version posted March 26, 2026. The copyright holder for this preprint (which was not certified by peer review) is the author/funder, who has granted bioRxiv a license to display the preprint in perpetuity. It is made available under aCC-BY 4.0 International license.

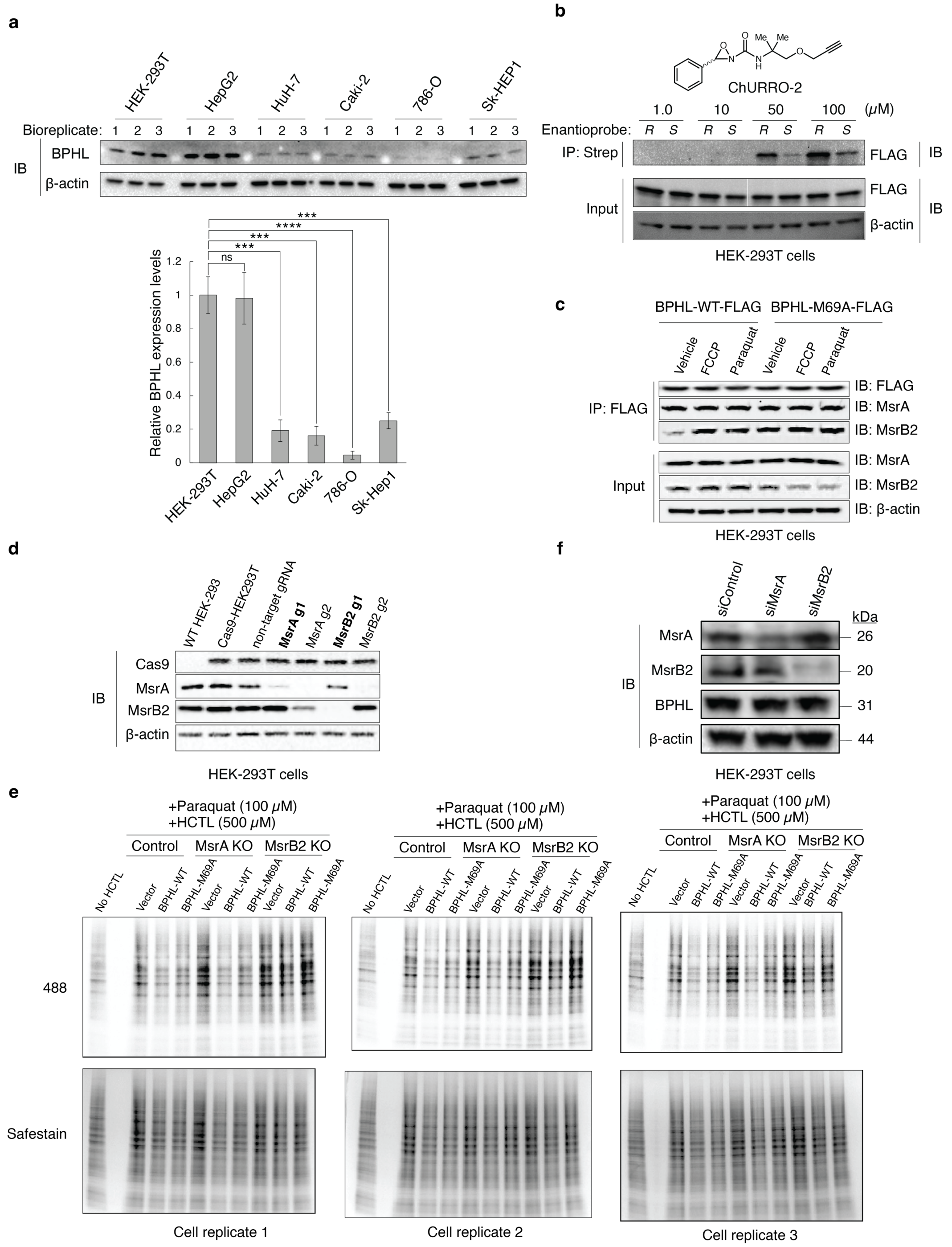

Supplementary Fig. 7: Assessing the impact of BPHL prochiral methionine oxidation on cellular *N*-homocysteinylation status. a, BPHL expression screen in renal and hepatic cell lines assessed by immunoblotting (n = 3 technical cell replicates, error bars represent standard deviation). **b**, Immunoprecipitation studies of FLAG-BPHL expressed in HEK-293T cells using (*R*)- and (*S*)-ChURRO-2. **c**, Representative immunoblot of BPHL Co-IP with MsrA and MsrB2 (n = 3 cell technical replicates). **d**, Immunoblot analysis of MsrA and MsrB2 KO efficiency. **e**, In-gel fluorescence data of labeled *N*-homocysteinylated proteins in HEK-293T cells with MsrA or MsrB2 KO (n = 3 technical cell replicates). **f**, Assessment of Msr knock-down efficiency in HEK-293T cells.
